# Robotic-assisted total knee arthroplasty does not increase procedure duration or adverse event incidence: a retrospective comparative cohort study in a secondary public hospital

**DOI:** 10.1007/s00590-026-04860-5

**Published:** 2026-07-06

**Authors:** Michael Singh, Fraser Labrom, Jonathan Bigwood, Corey Scholes, Lorenzo Calabro

**Affiliations:** 1 University of Queensland, Brisbane, Australia; 2 Queen Elizabeth II Jubilee Hospital, Brisbane, Australia; 3 EBM Analytics, Crows Nest, NSW Australia

**Keywords:** Arthroplasty, Total Knee Arthroplasty, Robotic arthroplasty, Operative Time, Adverse events, public hospital, 90 day outcomes, knee, infection

## Abstract

**Background:**

Total knee arthroplasty (TKA) with various guidance methods for bone cuts and soft-tissue balancing yields excellent outcomes for end-stage knee osteoarthritis, offering durable pain relief and functional restoration. However, there is a growing interest in further enhancing intraoperative accuracy and consistency, with the aim of reducing complications and reducing recovery times through the introduction of robotic-assisted surgery (RAS). As with any new technology, concerns remain regarding the surgeon learning curve, operative efficiency, and safety profile of RAS compared to conventional techniques. This study evaluates the clinical utility of a RAS system (ROSA, Zimmer Biomet) in a public hospital setting, assessing its impact on procedure duration and adverse event incidence at 90 days postoperatively.

**Methods:**

A retrospective comparative cohort study was conducted at a secondary public hospital (Queen Elizabeth II Jubilee Hospital) in, Brisbane, Australia. Data was extracted from a departmental registry, electronic medical records, and intraoperative reports from September 2017 to February 2023. The study included 568 TKA cases: 173 instrumented or navigated TKAs performed before RAS introduction (Pre-RAS), 258 robotic-assisted TKAs after RAS adoption (RAS group), and 137 TKAs performed by other department surgeons who did not use RAS (non-RAS), serving as a benchmark for department-wide outcomes. The primary outcomes were procedure duration and adverse event incidence including surgical site infection (SSI), venous thromboembolism (VTE), knee stiffness, and all-cause readmission within 90 days postoperatively.

**Results:**

Among 67 recorded adverse events, no significant differences in total adverse event incidence were observed between the pre-RAS and RAS groups (12.1% vs. 11.6%, *p* = 0.78). A non-significant increase in superficial infections in males undergoing RAS-TKA was observed (*p* = 0.062). The pre-RAS group had significantly longer operative times than the RAS group (128 ± 21.6 min vs. 121.4 ± 19.5 min, *p* < 0.01). The non-RAS department group had shorter procedure durations than the pre-RAS group (118.3 ± 20.1 min, *p* < 0.01).

**Conclusion:**

RAS-TKA was associated with shorter operative times and a comparable safety profile to non-robotic TKA in this cohort. Further investigation is warranted to determine whether these findings are maintained across different settings and over longer follow-up periods.

**Supplementary Information:**

The online version contains supplementary material available at 10.1007/s00590-026-04860-5.

## Introduction

Total knee arthroplasty (TKA) remains the gold-standard treatment modality for end-stage knee osteoarthritis [1]. Despite excellent short- and long-term clinical outcomes [2, 3], there remains great interest in pursuing optimisation of TKA delivery to maximise clinical outcomes, while minimising adverse events and resource usage. As such, robotic-assisted surgery (RAS) is rapidly developing as a variation upon the traditional instrumented TKA which has shown promise in achieving these goals [4].

RAS TKA purported benefits include improved pre-operative planning resources and contemporaneous intra-operative assessment of dynamic ligamentous and osseous balancing [5, 6]. Recent meta-analysis has shown that RAS enables a more precise implantation of prosthesis components and reduction in overall mean blood loss [7]. However, there is a lack of data which shows this consistently translates into improved efficiency and reduction of patient adverse outcomes incidence.

The hesitancy surrounding RAS TKA uptake has largely been attributed to assumed increased operative time, costs/resource usage, potential complications in addition to the expected learning curve with adopting a new operative protocol [8]. Earlier Markov decision modelling studies suggested computer-navigated TKA as initially more costly at the time of index surgery compared to instrumented methods [9, 10], with the later cost benefits of decreased revision rates only being realised in higher volume tertiary centres [11]. In a recent study, RAS was shown to be comparable to computer-navigated TKA in total in-hospital costs despite a reduced in-hospital rehabilitation timeframe [12]. However, this study was again performed in a high-volume tertiary centre.

Although benefits have been shown in tertiary centres, there remains a clear gap in the literature for an investigation which details the implementation of these robotic-assisted arthroplasty systems in a smaller, secondary public hospital setting. This study aims to assess the use of a RAS system (ROSA, Zimmer Biomet) for TKA on procedure duration, readmission and reoperation rates (and other adverse events) at 90 days followup, in a public hospital setting. It was hypothesised that RAS for TKA would provide equivalent or reduced procedure duration and comparable adverse event rates compared with non-robotic delivery systems within the department.

## Methods

### Study design

This retrospective comparative cohort study sourced data from a quality departmental registry, electronic medical records and case reports from the intraoperative systems of interest. The case-control retrospective analysis provides the most robust comparison between the conditions of having the RAS present versus absent in the same surgeon group, as well as benchmarking against the outcomes of the hospital department.

### Setting

Queen Elizabeth II Jubilee Hospital, a secondary public hospital in Australia. The hospital is part of a local health network spanning a region of the city and performs the majority of total knee arthroplasty cases within the network. The department registry was implemented in July 2017. The registry utilises prospectively collected data to aid in the construction of observational cohorts for case-control analyses. The implementation and quality assessment of this particular department registry has been previously published [13]. The robotic assisted surgical system (RAS) was introduced in January 2021 and the first case for the department performed 19-Feb-2021.

### Registration and ethics

Ethical approval was obtained from the Metro South Research Office (HREC No: HREC/2023/QMS/95442), with a waiver of consent approved for retrospective chart review.

### Funding

This research received no specific grant from any funding agency in the public, commercial, or not-for-profit sectors. No funding sources had any role in the design, conduct, analysis, or reporting of this study.

### Participants and grouping

This study included a total of 568 patients undergoing primary total knee arthroplasty at the study institution between September 2017 and February 2023. This included 173 computer assisted navigation or intramedullary jig based TKAs using Zimmer Persona (Zimmer Biomet) TKA implants (pre-RAS group) performed prior to the introduction of robotic-assisted knee arthroplasty systems at the study site by surgeons who now routinely employ robotic-assisted TKA (Surgeons A, B, C). The surgeons contributing to the Pre-RAS cohort had established experience with Zimmer Persona total knee arthroplasty prior to implementation of robotic-assisted surgery, each having performed more than 30 cases using navigation-assisted or conventional instrumentation techniques. The RAS Group included 258 primary robotic TKAs using Zimmer Persona (Zimmer Biomet) implants, irrespective of surgeon (contributions from Surgeons A, B, C, E, F, G). Surgeons contributing exclusively to the RAS group (Surgeons E, F, G) either transitioned to the ROSA system from a different implant system or joined the department following RAS implementation in 2021. The remaining 137 instrumented or navigated TKAs performed by four other departmental surgeons (Surgeons D, E, F, G) were assigned to a non-RAS group, using a variety of implants.

Patients were included if they underwent primary total knee arthroplasty between September 2017 and February 2023, with degenerative knee joint disease as the primary indication for surgery. All cases were performed under the care of a consultant orthopaedic surgeon. Records for patients receiving a total knee arthroplasty with or without the involvement of the robotic-assisted surgical (RAS) system (ROSA, Zimmer-Biomet, USA) were included for review and analysis.

Exclusion criteria for the RAS system of interest included;

- Hip pathology with significant bone loss (e.g. avascular necrosis of the femoral head with collapse, severe dysplasia of the femoral head or the acetabulum).
- Hip pathology severely limiting range of motion (e.g. arthrodesis, severe contractures, chronic severe dislocation).
- Active infections of the knee joint area.
- Revision TKA surgery.

### Outcomes

All demographic data and patient outcomes were retrospectively collected through chart review by three researchers. Demographic data included age, sex, BMI, comorbidities and side of surgery.

The primary outcomes of this study are adverse events within 90 days post-surgery and procedure duration (time from skin incision to final skin closure). Adverse events (up to 3 months) is defined as any deviation from the normal clinical course of treatment or recovery including: in-hospital complication event, post-discharge representation, post-discharge readmission, and/or reoperation. Adverse events have been subcategorised into the following categories [14]: *Bleed*,* Thromboembolisms*,* Neural injury*,* Vascular injury*,* Stiffness*,* Infection (Superficial*,* Deep*,* Systemic)*,* Periprosthetic fracture*,* Patellofemoral pain*,* Delivery*,* Readmission*,* Reoperation*,* Revision*.

### Data sources and measurement

#### Chart review

All relevant registry encounters between July 1 2017 and March 1 2023 were reviewed. All Individual patient charts were accessed through the integrated electronic medial record installed statewide (Cerner, USA). The record of encounters was reviewed by three reviewers and descriptions of any deviation from the normal trajectory of recovery (adverse events) were documented in an electronic form for linkage to the study master database. Details regarding procedure duration, intraoperative surgical technique (including robotic/navigation/ instrumented), implant components, patient demographics and comorbidities were also recorded from the electronic medical record. Intraoperative surgical technique was also cross referenced from the departmental registry as well as case records from the ROSA system records to ensure accuracy. Any conflicting information was physically reviewed to ensure data collection was accurate to the procedure.

#### Study size

As this was a retrospective cohort study, all consecutive primary total knee arthroplasties meeting the inclusion criteria and performed at the study institution between September 2017 and February 2023 were included in the analysis. Sample size was therefore determined by the number of eligible cases available within the study period. Post hoc power analysis of the available sample demonstrated sufficient power for the primary study outcomes (Supplementary material 6.8). The non-inferiority margin was set a priori at a 17% relative increase over the baseline 90-day superficial surgical site infection rate. The baseline rate was taken from the pre-RAS cohort (2.3% at 90 days). This corresponds to an absolute increase threshold of 0.39% points (2.3% × 0.17), i.e., an upper limit of ~ 2.7% for the RAS group. This margin was chosen as a clinically small increase in minor SSI risk that would be acceptable given the anticipated workflow and alignment benefits of RAS.

### Arthroplasty intervention

#### Patient selection

Patients undergoing primary TKA at Queen Elizabeth II Jubilee Hospital between September 2017 and February 2023 were included in the study. They were excluded if there was active infection, underwent revision TKA, had hip pathology with bony loss or had hip pathology significantly reducing ROM. Complex primary (e.g. those requiring varus/valgus constraint or hinged options, post osteotomy and post traumatic arthritis) arthroplasty cases were excluded.

#### Hospital setting

The procedures were performed at Queen Elizabeth II Jubilee Hospital, a secondary public teaching hospital in Australia. All cases were performed either by a consultant orthopaedic surgeon (71% of all cases) or by a training surgeon under the supervision of a consultant orthopaedic surgeon (29% cases overall).

#### General surgical technique considerations

General surgical technique, including approach, alignment philosophy, decision for patella resurfacing/release, use of tourniquet, and choice of implants were up to the discretion of the lead surgeon. However, on balance, the medial parapatellar approach, patellar resurfacing and tourniquet use were the predominant practice across the surgeon cohort (see supplementary material for full breakdown), and implant selection was standardised within the Pre-RAS and RAS groups, both of which exclusively utilised the Zimmer Persona system. Variation in these technique parameters was most pronounced within the Non-RAS group, consistent with its role as a contemporaneous departmental benchmark rather than a direct experimental control.

#### Instrumented surgical technique

Instrumented technique was performed with intramedullary femoral alignment and extramedullary tibial alignment as per the surgical technique from Zimmer-Biomet (Persona, Zimmer-Biomet, USA), Stryker (Triathlon, Stryker, Germany), Depuy (Attune, Depuy Synthes, USA) or Smith and Nephew (Genesis II, Smith and Nephew, USA).

#### Computer assisted navigation surgical technique

Computer assisted navigated technique was performed as per the surgical technique from Zimmer-Biomet (Orthosoft, Zimmer-Biomet, USA) or Stryker (Orthomap Precision, Stryker, Germany).

#### Robot assisted surgical technique

RAS surgical technique was performed as per the surgical technique from Zimmer-Biomet (ROSA, Zimmer-Biomet, USA) using Zimmer Persona implants.

### Data and statistical analysis

All data for the primary outcome and procedure duration (secondary outcome) were present.

To assess the effect of *Group* on the total incidence of adverse events at up to 90 days follow up, a gamma regression (see supplementary material, Table 22) was generated to assess the effect of *group* on proportions of events, with adjustment for covariates. Gamma regression was selected because both procedure duration and adverse event proportion distributions are right-skewed and strictly positive, violating the assumptions of ordinary least squares regression; the gamma family accommodates these properties without requiring data transformation. The DAG framework was used to define the minimal covariate adjustment set based on a pre-specified causal model, rather than statistical significance, thereby reducing the risk of collider bias and over-adjustment. Model-predicted marginal estimates with 95% confidence intervals are reported to provide clinically interpretable effect sizes. Full model specification, diagnostic plots and code are documented in the supplementary report (Sect.  5.14.3 and 5.14.4). Odds ratios were calculated and retrieved for the model variables with standard errors and p-values. Model-predicted margins with 95% confidence intervals were used to assess the difference between the RAS group and the non-inferiority margin. Non-inferiority was declared if the upper limit of the one-sided 95% confidence interval for the marginal estimate of adverse event incidence in the RAS group did not exceed a relative difference of 17% from the baseline incidence (as per sample size justification).

Procedure duration was fitted to a mixed-effects gamma regression model utilizing the minimal adjustment set identified in the DAG (see supplementary material, Figs. 18 and 19). Residual fits and leverage points were plotted to assess the fit of the procedure duration model.

Sensitivity analyses were performed to assess the impact of early learning curve cases on operative duration and complication incidence. These analyses confirmed that inclusion of initial robotic-assisted cases did not bias the findings. Temporal analysis of adverse event development was conducted using Cox proportional hazards models to generate adjusted survival curves stratified by surgical group and adjusted for relevant covariates. Missing data patterns were assessed and visualised using naniar, and relevant demographic data were supplemented using cross-linked registry records where available. Full data preparation and modelling procedures are documented in the supplementary report. Subgroup interaction effects, including those by sex, were evaluated by incorporating interaction terms into the regression models; significance was assessed using the p-value of the interaction term, with *p* < 0.05 considered statistically significant.

## Results

### Patient characteristics and group comparison

Registry data collated over the study course included the record of 588 total surgical encounters across the three groups. Encounters were subsequently examined for eligibility, with 568 surgical encounters ultimately included for comparison at the primary outcome timepoint of 3-months post-operatively. 20 records were excluded: four due to pre-operative cancellation, four due to unavailable medical records, one due to duplication, and eleven due to incorrectly recorded surgery type. The RAS group of surgeons recorded 258 surgical encounters, 3 of the 6 surgeons, who contributed cases to the RAS group had routinely performed non robotic assisted surgery using the same implant from 2017 to 2021 prior to introduction of the RAS. These ‘pre robotic’ cases numbered 173 and were assigned to pre-RAS group. All encounters in the pre-RAS and RAS groups used the same TKA implants (Zimmer Biomet Persona). The non-RAS group recorded 137 surgical encounters. The Pre-RAS surgeons contributed 59% of RAS cases with surgeons EFG contributing 0% to Pre-RAS and 42% RAS. There were no statistically significant differences between groups in respect to patient demographics, time-frame of recruitment, and surgeon (Table 1).

**Table 1:** Surgeon breakdown, cohort demographics and alignment referencing modality as stratified by surgeon TKA delivery groups

Characteristic	Overall *N* = 568^a^	RAS *N* = 258^a^	Pre-RAS *N* = 173^a^	Non-RAS *N* = 137^a^
Surgeon				
A	55 (9.7%)	10 (3.9%)	45 (26%)	0 (0%)
B	98 (17%)	44 (17%)	54 (31%)	0 (0%)
C	170 (30%)	96 (37%)	74 (43%)	0 (0%)
D	46 (8.1%)	0 (0%)	0 (0%)	46 (34%)
E	38 (6.7%)	31 (12%)	0 (0%)	7 (5.1%)
F	26 (4.6%)	25 (9.7%)	0 (0%)	1 (0.7%)
G	135 (24%)	52 (20%)	0 (0%)	83 (61%)
Surgery date	2017-09-15–2023-02-07	2021-02-19–2023-01-19	2017-09-15–2023-02-07	2021-02-02–2023-01-30
Surgery side				
Left	278 (49%)	131 (51%)	88 (51%)	59 (43%)
Right	290 (51%)	127 (49%)	85 (49%)	78 (57%)
Alignment referencing				
Im Femur/Em Tibia	116 (21%)	0 (0%)	23 (14%)	93 (68%)
Navigation	183 (33%)	0 (0%)	140 (86%)	43 (32%)
Robotic	254 (46%)	254 (100%)	0 (0%)	0 (0%)
Unknown	15	4	10	1
Age at surgery	69 (62, 76)	69 (64, 76)	68 (61, 75)	70 (63, 77)
Unknown	3	3	0	0
Sex				
Female	322 (57%)	141 (55%)	93 (54%)	88 (64%)
Male	246 (43%)	117 (45%)	80 (46%)	49 (36%)

n (%); Min - Max; Median (Q1, Q3)

### Adverse events

From the 568 encounters, a total of 67 adverse events were recorded within the primary outcome variable timeframe of 90 days. Of these, 38 were re-admissions, 24 were re-operation procedures and two revision arthroplasty procedures. Adverse event incidence and adjusted likelihood of adverse event rate was calculated by multi-state models. Re-admission (16 (6.2%) vs. 8 (4.6%) vs. 14 (10%) (*p* = 0.13)), re-operation (12 (4.7%) vs. 4 (2.3%) vs. 8 (5.8%) (*p* = 0.3)) and revision rates (0 vs. 0 vs. 2 (1.5%) *p* = 0.58) were comparable between groups. A complete breakdown is tabulated in Table 2.

**Table 2:** Adverse event and subsequent intervention incidence rates separated by Group

Characteristic	Overall *N* = 568^a^	RAS *N* = 258^a^	Pre-RAS *N* = 173^a^	Non-RAS *N* = 137^a^	*p*-value^b^	q-value^c^
Bleed	7 (1.2%)	3 (1.2%)	1 (0.6%)	3 (2.2%)	0.4	0.6
Thromboembolism	15 (2.6%)	5 (1.9%)	6 (3.5%)	4 (2.9%)	0.6	0.7
Neural	4 (0.7%)	1 (0.4%)	2 (1.2%)	1 (0.7%)	0.8	0.9
Vascular	1 (0.2%)	1 (0.4%)	0 (0%)	0 (0%)	> 0.9	> 0.9
Stiffness	9 (1.6%)	7 (2.7%)	0 (0%)	2 (1.5%)	0.061	0.4
Infection Superficial	18 (3.2%)	10 (3.9%)	4 (2.3%)	4 (2.9%)	0.7	0.8
Infection Deep	1 (0.2%)	0 (0%)	0 (0%)	1 (0.7%)	0.2	0.5
Infection Systemic	2 (0.4%)	0 (0%)	1 (0.6%)	1 (0.7%)	0.3	0.5
Periprosthetic Fracture	3 (0.5%)	0 (0%)	1 (0.6%)	2 (1.5%)	0.095	0.4
Patellofemoral Pain	3 (0.5%)	1 (0.4%)	2 (1.2%)	0 (0%)	0.5	0.6
Delivery System failure	4 (0.7%)	4 (1.6%)	0 (0%)	0 (0%)	0.2	0.4
Readmission	38 (6.7%)	16 (6.2%)	8 (4.6%)	14 (10%)	0.13	0.4
Reoperation	24 (4.2%)	12 (4.7%)	4 (2.3%)	8 (5.8%)	0.3	0.5
Revision	2 (0.4%)	0 (0%)	0 (0%)	2 (1.5%)	0.058	0.4

^a^n (%)

^b^Fisher’s exact test; Pearson’s Chi-squared test

^c^False discovery rate correction for multiple testing

**Fig. 1:**
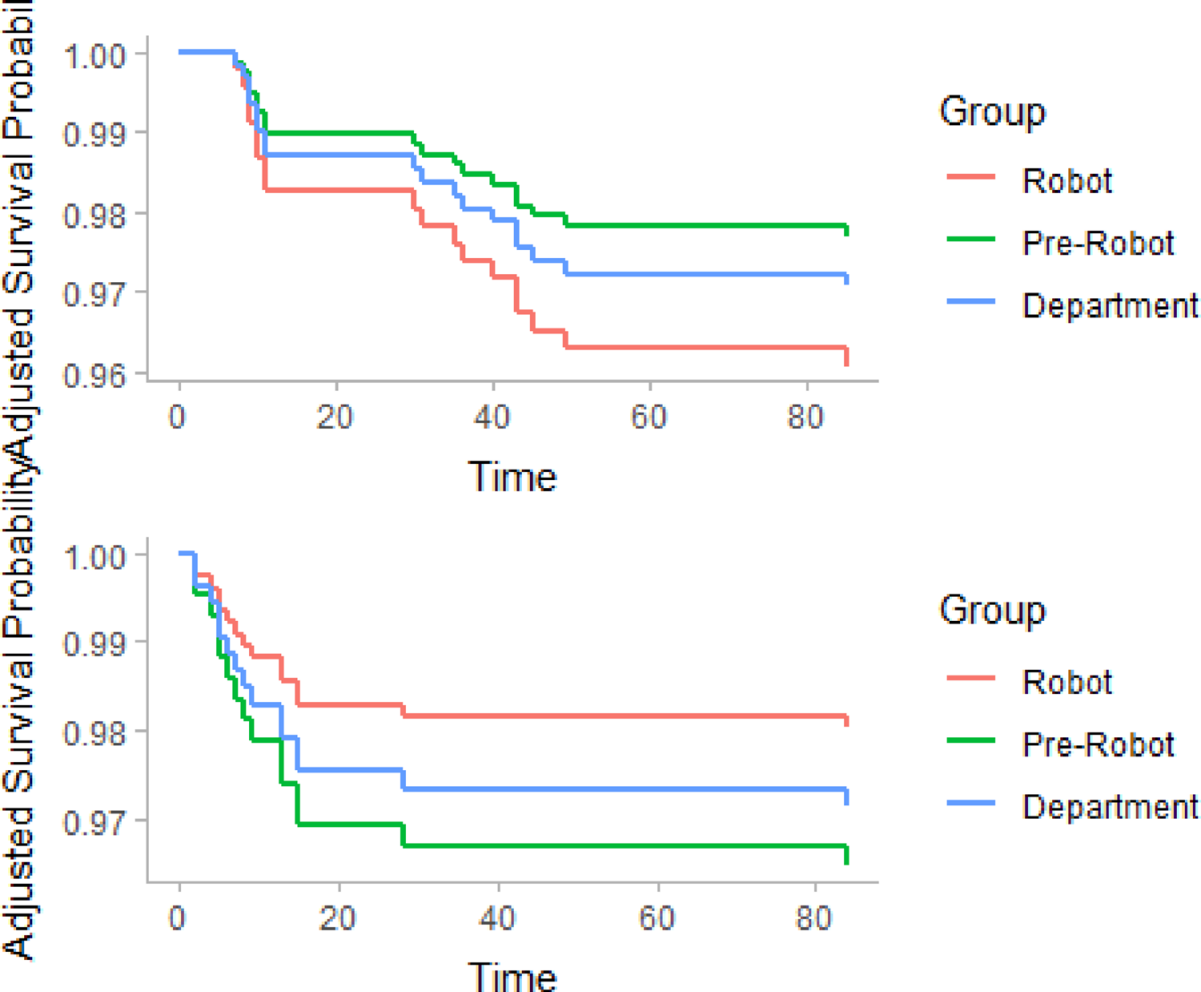
Survival curve of primary adverse event outcome variable of superficial infection (% incidence) (top) and VTE (bottom) over the postoperative follow up period (days). Survival rates are stratified by Group (RAS, Pre-RAS, Non-RAS)

**Fig. 2:**
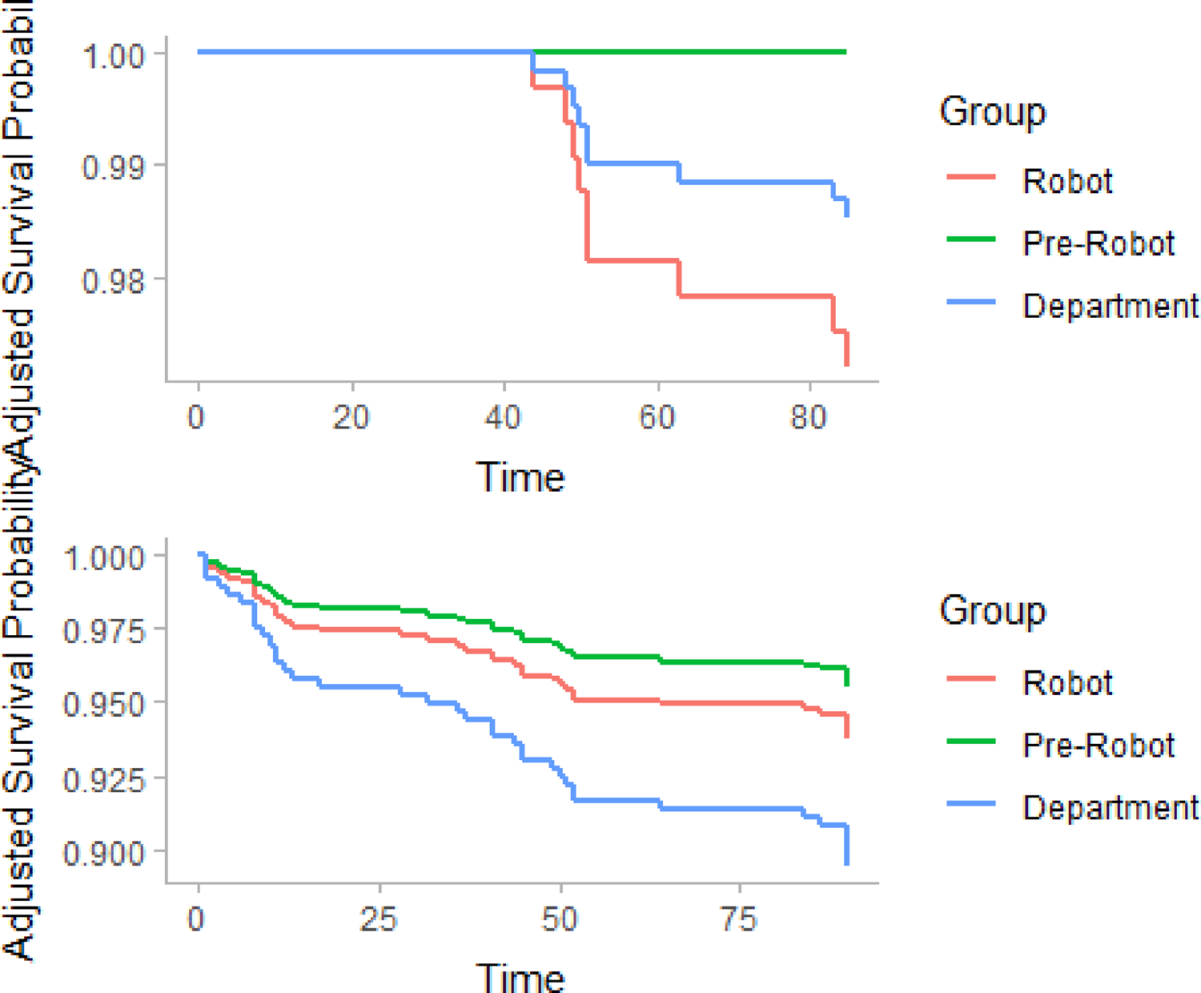
Incidence model curve of primary adverse event outcome variable of stiffness (% incidence) (top) and all-cause readmission (bottom) over the postoperative follow up period (days). Incidence rates are stratified by TKA mode of delivery surgeon groups (RAS, pre-RAS, non-RAS)

TKA delivery modality had no significant effect upon incidence of SSI, VTE, stiffness or all-cause readmission after TKA in the first 90 days after surgery (Table 3). Temporal development of these adverse events were examined utilising adjusted survival curves (Figs. 1 and 2). When examined via sub-group interaction effect analysis, the cumulative incidence of superficial infection at 90-day follow up for male sex was 6% [95% CI 2–10%], but this did not reach significance when compared to the male population within other groups (*p* = 0.062). Further statistical analysis data outputs are available within supplementary material.

**Table 3:** Summary of adjusted Cox proportional hazards models for surgical site infection, venous thromboembolism, stiffness and all-cause readmission

Characteristic	Superficial Infection			Thromboembolism			Stiffness			Readmission		
	HR	95% CI	*p*-value	HR	95% CI	*p*-value	HR	95% CI	*p*-value	HR	95% CI	*p*-value
Group1												
Non-RAS	0.75	0.24–2.27	0.6	1.44	0.37–5.63	0.6	0.53	0.11–2.53	0.4	1.70	0.83–3.52	0.15
Pre-RAS	0.58	0.18, 1.92	0.4	1.80	0.54, 6.04	0.3	0.00	0.00, Inf	> 0.9	0.66	0.28, 1.55	0.3
RAS	1.34	0.44, 4.10	0.6	0.69	0.18, 2.70	0.6	1.90	0.39, 9.17	0.4	0.59	0.29, 1.20	0.14
Sex (Male)	1.05	0.42, 2.64	> 0.9	0.64	0.21, 2.00	0.4	2.95	0.74, 11.8	0.13	1.81	0.95, 3.44	0.072
Smoking History	0.98	0.13, 7.67	> 0.9	1.01	0.12, 8.29	> 0.9				0.55	0.08, 3.81	0.5
Diabetes				1.49	0.39, 5.66	0.6	0.00	0.00, Inf	> 0.9			
Cardiovascular Disease										0.00	0.00, 0.00	> 0.9

CI = Confidence Interval, HR = Hazard Ratio

### Procedure duration

**Fig. 3:**
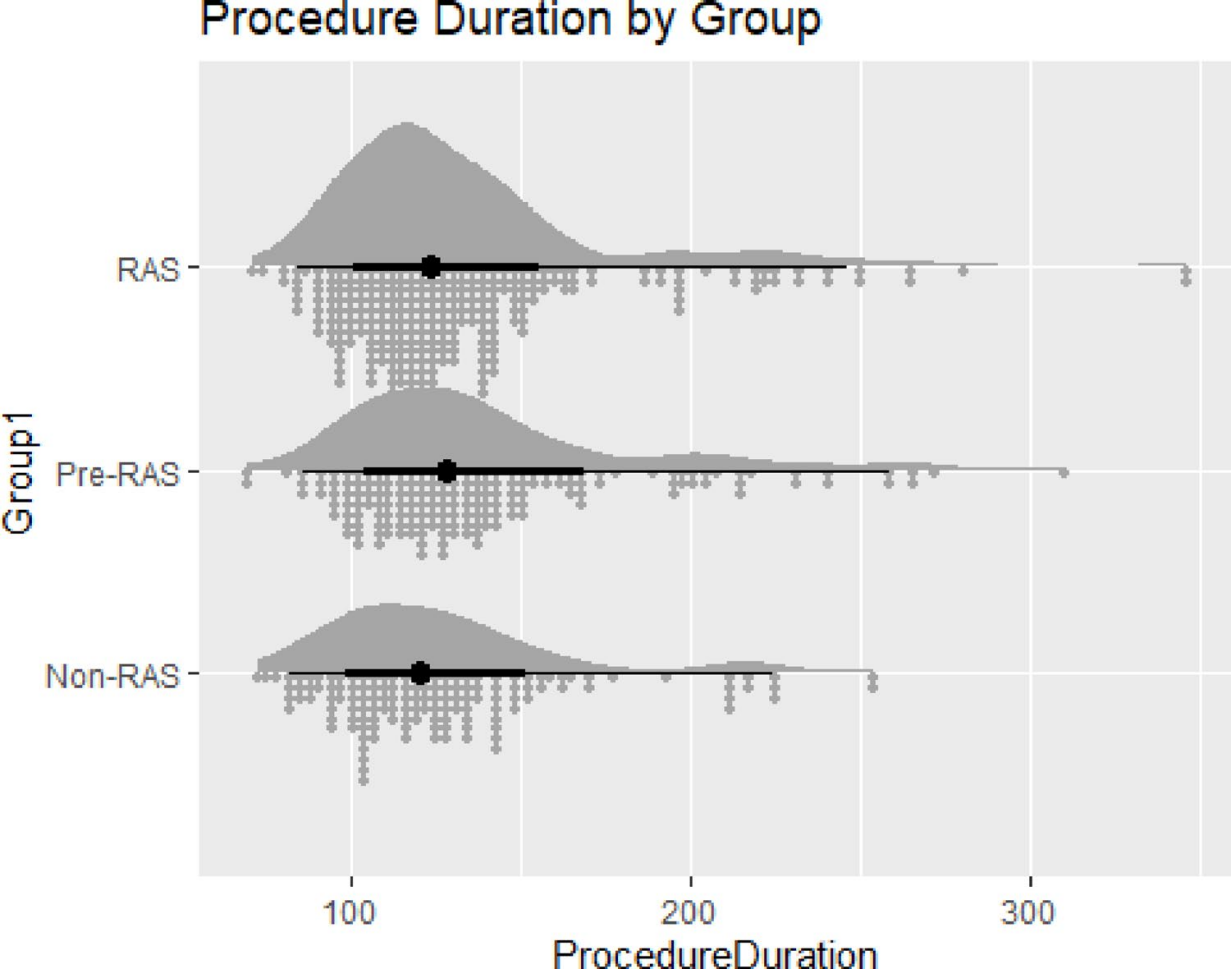
Procedure duration (minutes) for individual study groups with depicted median, inter-quartile ranges and minimum and maximum values shown. Median (IQR1–IQR3) procedure times were 120 (103–142) minutes (mins) for the non-RAS department group; 128 (110–151) mins for the pre-RAS group; and 123 (108–143) mins for the RAS group

A statistically significant difference in operative procedure time was observed between the RAS and pre-RAS groups (∆6.55(2.27) mins; *p* < 0.01). Mean ± standard deviation (SD) procedure durations were: 128 ± 21.6 min (Pre-RAS), 121.4 ± 19.5 min (RAS), and 118.3 ± 20.1 min (non-RAS). The Pre-RAS group had a procedure duration of 128 (110–151) minutes, RAS group 123 (108–143), and non-RAS group of 120 (103–142). When comparing procedure duration, the non-RAS group was statistically significantly shorter compared to the pre-RAS group (∆-9.68(3.24) (mean(standard error) minutes (mins); *p* < 0.01). The procedure duration was statistically comparable between non-RAS and RAS groups (∆ − 3.13(3.13)mins; *p* = 0.32). This data is graphically represented in Fig. 3.

## Discussion

Our key findings indicate that robotic-assisted surgery can be delivered in a public hospital setting without increasing the incidence of adverse events in the 90-day post-operative period. We also observed a modest reduction in procedure duration in the RAS group when compared with the pre-RAS group. Importantly, this study design included a pragmatic comparison group of procedures performed by the same surgeons in the same setting, prior to and during adoption of RAS.

This corroborates with current knowledge that RAS or non-RAS technique implementation does not affect the rates of overall adverse event occurrence [6, 15]. The strength of our study is that we were able to directly compare non-robotic arthroplasty with RAS through a pragmatic study with largely the same surgeons and implants in a teaching public hospital. We were able to track this through the temporal transition to RAS from CAS in the public hospital department. Of the 568 total cases included in this study, there were 67 overall adverse events with no significant difference observed between groups for total adverse event incidence. The rates of readmission and reoperation (non-revision) were comparable between RAS and non-RAS groups. Whilst post-operative stiffness was observed at a higher rate in the RAS group (2.7%) than the non-robotic groups (0%, 1.5%), this was not a statistically significant result when corrected for multiple testing (q = 0.4). These findings are further supported by the adjusted survival curves presented in Figs. 1 and 2, which illustrate no significant temporal differences in the incidence of superficial infection, venous thromboembolism, postoperative stiffness, or all-cause readmission across the RAS, pre-RAS, and non-RAS groups during the 90-day follow-up period. Current literature suggests that RAS TKA yields lower rates of post-operative stiffness as evidenced by lower rates of manipulation under anaesthesia in robotic cases [15,16, 17]. Of note, sex-stratified subgroup analysis revealed that males who underwent RAS TKA in our cohort had a higher cumulative incidence of superficial infection at 90 days (survival rate: 94% [95% CI 90–98%]; *p* = 0.062), though this did not reach statistical significance. Low numbers of event occurrence limit capacity to make sound conclusions, this difference approaches our statistical significance threshold and certainly represents an area for further investigation.

Current literature reliably demonstrates that increased surgical duration is associated with higher risk of subsequent prosthetic joint infection [24]. Since introduction of RAS TKA at the study site, there was a small but statistically significant decrease in operative time. There was no significant difference in procedure duration between the RAS group and non-RAS control cohort. Current trends in literature tend to suggest that conventional TKA techniques yield shorter operative times than RAS TKA [25]. Of note, our pre-RAS cohort was composed of primarily navigated TKA cases (86%) in contrast to our non-RAS group which predominantly utilized instrumented techniques (68%). Navigated TKA has previously been demonstrated to produce longer procedure durations than instrumented TKA [26] and there is emerging evidence to suggest navigated TKA may in fact yield longer procedure durations than RAS TKA [27]. It is acknowledged that the difference in alignment referencing modality between the Pre-RAS group (86% navigated) and Non-RAS group (68% instrumented) represents a potential source of confounding when comparing operative times across these groups. However, we would note that this difference in platform composition was not incidental—it accurately reflects the technology transition that occurred at our institution and was the subject of this pragmatic evaluation. Furthermore, the primary comparison of interest, Pre-RAS versus RAS, is the least susceptible to this confound, as both groups involved the same surgeons performing procedures with the same implant system. These findings are corroborated by our study. An additional point of consideration when interpreting operative times trends is surgeon experience with RAS. Chen et al. [28] have described the significant learning curve associated with RAS TKA. They have also shown that a given surgeons’ operative time tends to decrease for approximately the first twenty RAS TKA cases and approximately two-thirds of RAS-utilizing surgeons will achieve the same operative times as instrumented technique following this initial learning curve. Given the study design, the RAS group included each surgeon’s early cases following implementation of the robotic platform. However, the surgeons contributing to the pre-RAS cohort were not in their learning curve for the implant system itself, having accumulated substantial experience with Zimmer Persona arthroplasty using navigated and/or conventional instrumentation techniques prior to robotic adoption. Consequently, the observed differences in operative time should be interpreted within the context of both technology platform and surgeon experience.

There is a paucity of high-level evidence on the incidence of surgical site infections in robotic-assisted TKA in comparison to instrumented and navigated methods. Meta-analysis [15] of studies investigating surgical site infection in robotic knee arthroplasty has reported superficial surgical site infection incidence as low as 0.57% (CI = 0.209–0.927). Our findings demonstrate no change in superficial infection incidence since the introduction of RAS at the study site. Whilst higher incidence of prosthetic joint infections amongst males undergoing arthroplasty has been well-documented [18], Keemu et al. [19] attribute this to largely to the increased rates of smoking and alcohol consumption in the male population - both independent risk factors for prosthetic joint infections [20]. The interaction between gender and TKA delivery system on post-operative infection has not been previously reported to the authors’ knowledge.

The clinical significance of early superficial infection post-TKA is poorly documented in current literature. Galat et al. [21] has previously described an increased occurrence of deep tissue infection (6.0% vs. 0.8%) and re-operation (5.3% vs. 0.6%) within two years amongst those requiring incision and drainage for superficial infections post-TKA. Previous studies have identified infection depth as a key prognostic factor in post-operative infections following TKA. Extrafascial infections treated with irrigation and debridement have been demonstrated to have higher long-term prosthesis retention rates than deeper (subfascial) infections treated identically [22, 23]. Long-term outcomes for TKAs complicated by superficial infections treated non-surgically are currently not well reported in literature.

## Limitations

This study has several limitations. A key limitation of this study is the variability within groups, which reflects the real-world way robotic-assisted surgery was introduced at our institution. Surgeons adopted the technology at different times, and the case-mix naturally shifted as new consultants joined the department. This means the groups are not perfectly homogenous in terms of surgeon experience, technique history or patient selection. While this captures actual clinical practice, it also introduces within-group variability that may dilute subtle differences between delivery methods. The inclusion of low-volume surgeons may also influence short-term adverse event rates, given the recognised relationship between surgical volume and early complications. These factors should be considered when interpreting comparisons across groups. To directly quantify the impact of surgeon composition on the primary outcome, a sensitivity analysis extending the procedure duration model to include surgeon as a crossed random effect demonstrated that surgeon identity accounted for less than 4% of total outcome variance (ICC = 0.040), providing empirical support for retaining the full pragmatic cohort as the primary analysis.

The retrospective cohort design introduces inherent risk of information bias, as it relies on the accuracy and completeness of routinely collected clinical and registry data. While three investigators independently reviewed charts and cross-checked registry and intraoperative system data, inter-rater reliability was not formally assessed, and misclassification of variables may have occurred.

The grouping of patients based on surgeon use of robotic-assisted surgery (RAS) may introduce selection bias. Surgeons may have selected cases based on patient complexity, anatomical variation, or familiarity with technology, which were not fully captured in the dataset. Although statistical adjustment was undertaken using DAG-informed models, residual confounding from unmeasured variables remains possible.

While there was no missing data for primary outcomes, demographic and procedural variables had variable completeness. These were addressed through cross-linking with the departmental registry and hospital records. Despite thorough sensitivity analyses, residual confounding from unmeasured factors remains possible. Residual diagnostic plots for the procedure duration gamma regression indicated some departure from model assumptions, including non-linear residual patterns and influential observations; findings should therefore be interpreted with appropriate caution.

Generalisability is limited by the single-centre design and the exclusive use of one robotic platform (ROSA, Zimmer Biomet) as well as a single implant (Persona, Zimmer Biomet). These results may not apply to institutions with different patient populations, surgical workflows, or alternative robotic systems.

Whilst the total sample size was sufficient for the primary outcomes, the study was underpowered to detect differences in rare events such as deep infections, periprosthetic fractures, or revisions. Findings related to these outcomes should be interpreted with caution.

Finally, the study did not assess long-term outcomes such as implant survivorship or patient-reported functional scores. These measures are critical for evaluating the broader clinical impact of robotic-assisted TKA and should be the focus of future prospective studies.

## Conclusion

RAS-TKA was associated with shorter operative times and a comparable safety profile to non-robotic TKA in this cohort. While these findings support the feasibility of implementing robotic-assisted TKA in a public hospital setting, further studies are required to evaluate long-term clinical, functional and economic outcomes.

## Supplementary Information

Below is the link to the electronic supplementary material.

Supplementary Material 1

## Data Availability

The data supporting the findings of this study were generated from a prospectively maintained departmental orthopaedic registry and linked hospital electronic medical records. These data contain potentially identifiable patient information and are subject to institutional and ethics approval restrictions. As such, the datasets are not publicly available. De-identified data may be made available from the corresponding author upon reasonable request and with appropriate institutional and ethics approvals.
