# Supplementary material for: Robotic-assisted total knee arthroplasty does not increase procedure duration or adverse event incidence: a retrospective comparative cohort study in a secondary public hospital
Source: Eur J Orthop Surg Traumatol. 2026 Jul 6;36(1):276. doi: 10.1007/s00590-026-04860-5 (PMC13337853; doi:10.1007/s00590-026-04860-5)
Supplement: Supplementary file 1 — Supplementary Material 1 [file 590_2026_4860_MOESM1_ESM.docx]

Robotic-Assisted Total Knee Arthroplasty Does Not Increase Procedure Duration or Adverse Event Incidence: A Retrospective Comparative Cohort Study in a Secondary Public Teaching Hospital

2023-05-06

Table of contents

1. Preamble 1

2. STROBE [1] Title: 1

3. STROBE [1] Abstract: 1

4. Introduction 1

4.1 STROBE [2] Background 1

4.2 STROBE [3] Objectives 1

4.2.1 IDEAL Stage2b [1]: Definite evaluation 1

5. Methods: 1

5.1 STROBE [4] Study Design: 1

5.2 IDEAL Stage2b [2]: Protocol 1

5.3 STROBE [5] Setting: 1

5.4 Ready libraries 1

5.5 Authorisations 1

5.6 Processing functions 1

5.7 STROBE [8] Data Sources/Measurement: 1

5.8 Mastersheet Preparation 1

5.8.1 Hospital exports - Arthroplasty Activity 1

5.8.2 DEPARTMENTAL REGISTRY Snapshot and Export 1

5.8.3 Vendor report - RAS Activity 1

5.8.4 Chart Review 1 1

5.8.5 Chart Review 2 1

5.8.6 Analysis - Inputs 1

5.8.7 Prepare dataset 1

5.9 STROBE [6] Participants: 1

5.9.1 IDEAL Stage2b [2]: Inclusion-criteria 1

5.10 STROBE [7] Variables: 1

5.10.1 Exposure variables 1

5.10.2 IDEAL Stage2b [3] Outcomes 1

5.11 STROBE [9] Bias: 1

5.12 Missing Data 1

5.13 STROBE [11] Quantitative variables: 1

5.14 STROBE [12] Statistical Methods: 1

5.14.1 Descriptives 1

5.14.2 Regression preparation 1

5.14.3 Procedure Duration 1

5.14.4 Adverse events 1

5.15 STROBE [10] Study size: 1

5.16 IDEAL Stage2b [5]: Learning effect 1

6. Results: 1

6.1 STROBE [13] Participant Flow: 1

6.2 STROBE [14] Descriptive Data: 1

6.3 STROBE [15] Outcome Data: 1

6.4 STROBE [16] Main Results: 1

6.4.1 Procedure Duration 1

6.4.2 Adverse Events 1

6.5 STROBE [17] Other Analyses: 1

6.5.1 Model sensitivity - Procedure duration 1

6.5.2 Model sensitivity - Surgeon 1

7. Discussion: 1

7.1 STROBE [18] Key Results: 1

7.2 STROBE [19] Limitations: 1

7.3 STROBE [20] Interpretation: 1

7.4 STROBE [21] Generalizability: 1

7.5 Comparison with Existing Literature: 1

8. Ethical Considerations: 1

8.1 Informed Consent: 1

8.2 Ethical Approval: 1

8.3 Privacy and Confidentiality: 1

9. Conflicts of Interest: 1

9.1 STROBE [22] Funding: 1

9.2 Disclosure: 1

9.3 Potential for Biases: 1

10. References: 1

11. Analysis References 1

# 1. Preamble

**Author Affiliation:**

**Client | Sponsor:**

**EBMAReference**: Pub_Early_Outcomes_Robotic_Arthroplasty_CB048

**Version:** 4

This analysis is a companion piece to the manuscript of the project. The analysis report has been organised as per the STROBE guidelines and checklist (Vandenbroucke et al. 2007), as well as the IDEAL framework for surgical innovation (Ergina et al. 2013). The contents of the sections has been derived from the manuscript (partly completed at the time of drafting), the protocol approved by the local health district HREC 13-Jun-2023, as well as an earlier study viability report, and the results of the analysis, where appropriate. The analysis was generated using R version 4.4.2 (2024-10-31) in RStudio (v2024.04.0 Build 735) with appropriate specialised packages as required. Code and text were combined within the report using the epoxy package.

# 2. STROBE [1] Title:

A suggested title is the following;

*Introduction of a robotic system does not lead to increased procedure duration or adverse events incidence in total knee arthroplasty at up to 90 days followup. A retrospective comparative cohort analysis of a public hospital.*

# 3. STROBE [1] Abstract:

Included in the manuscript.

# 4. Introduction

Included in the manuscript.

## 4.1 STROBE [2] Background

Included in the manuscript.

## 4.2 STROBE [3] Objectives

The clarity and significance of the objectives have evolved over time. The original study question as per the study viability report was the following;

| Table 1: Study question as per study plan. Comparator updated **8-May-2024**   \| Component \| Study-Specific \| \| --- \| --- \| \| Population \| In patients presenting with end-stage knee degeneration electing to undergo lower limb total joint arthroplasty \| \| Intervention \| **What is the effect** of total knee replacement (posterior-stabilised \| medially-stabilised design, mechanical alignment) with robot-assisted guidance \| \| Comparator \| Compared to imageless navigated (Zimmer, Stryker) TKA *or manual instrumentation* for guidance of bone resection and component placement \| \| Outcomes \| On the  i) rate of treatment success/failure, (as defined by appropriate recovery in mechanical integrity of the implant and its placement, pain, function, satisfaction) at 3 months follow-up,  ii) length of stay, theatre time and CSD* as well as  iii) incidence/nature of complications at up to 3 months follow-up, when controlling for patient (demographic, anatomic), pathology and management (technique, approach, alignment guidance, balancing technique) factors. \| |
| --- | --- | --- | --- | --- | --- | --- | --- | --- | --- | --- |

This was summarised to the following aim in the HREC protocol;

- To assess the clinical utility and cost metrics (where available) before and after the introduction of the robotic-assisted surgery (RAS) system (ROSA, Zimmer Biomet) into total knee arthroplasty in a public hospital setting.
- Assess the effect of group on the total incidence of adverse events at up to 90 days follow up
- Assess the effect of group on procedure duration

No hypotheses were explicitly stated in the protocol. It is recommended that the following be considered for inclusion in the manuscript (in an appropriate form);

**Clinical hypotheses**

- Robotic assisted surgery (RAS) for total knee replacement provides equivalent or superior complications and patient-reported outcomes compared to non-robotic cases within the department
- RAS provides superior inputs for cost-benefit analysis relative to high-volume TKA in a public hospital setting
  - Length of stay (needs to be lower)
  - Adverse events (needs to be equivalent or lower)
  - Operative/theatre time (needs to be lower)
  - Tray usage (sterilisation) (needs to be lower)

**Statistical hypothesis**

- When adjusted for covariates, Group is significantly associated with theatre duration
- When adjusted for covariates, is Group significantly associated with complication (incidence/survival); discharge readiness (LoS, destination) and/or PROMs at 90-day follow-up?

### 4.2.1 IDEAL Stage2b [1]: Definite evaluation

The IDEAL framework recommends that a definitive evaluation be presented as a part of an observational study - and this has been achieved in this present analysis.

# 5. Methods:

## 5.1 STROBE [4] Study Design:

The case-control retrospective analysis designed for this analysis provides the most robust comparison between the conditions of having the RAS present versus absent in the same surgeon group, as well as benchmarking against the outcomes of the hospital department.

| 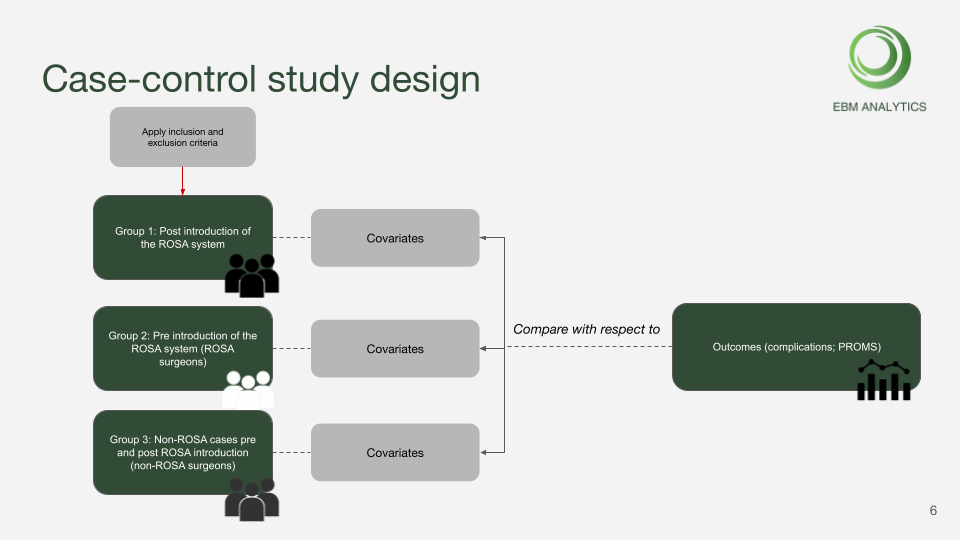  Figure 1: Proposed study design |
| --- |

## 5.2 IDEAL Stage2b [2]: Protocol

The author group will need to decide whether they wish to release the protocol (in full or in part) that was developed prior to data collection.

## 5.3 STROBE [5] Setting:

The study setting is an orthopaedic department within a medium-sized metropolitan public hospital in a capital city of Australia. The analysis and data collection have captured all eligible arthroplasty cases from the commencement date of the department clinical quality registry to the time of initial analysis.

## 5.4 Ready libraries

The environment was prepared by loading the required packages in advance; confirming that the necessary packages were already installed and if not, installing them. Where appropriate, citations to packages used appear at first use in the text.

if (!require("pacman")) install.packages("pacman")
pacman::p_load(# Load required packages
 "googledrive",
 "mets",
 "adjustedCurves",
 "googlesheets4",
 "readxl",
 "tidyverse",
 "tidymodels",
 "tidytext",
 "tidycmprsk",
 "tictoc",
 "stringr",
 "lubridate",
 "gt",
 "gtsummary",
 "consort",
 "survival",
 "survminer",
 "ggdag",
 "ggplot2",
 "ggdist",
 "ggsurvfit",
 "ggfortify",
 "mice",
 "marginaleffects",
 "patchwork",
 "naniar",
 "quantreg",
 "broom",
 "broom.helpers",
 "broom.mixed",
 "epoxy",
 "flextable",
 "wordcloud",
 "npsurvSS",
 "adjustedCurves",
 "riskRegression",
 "stopwords"

 )

| Table 2: Summary of package usage and citations   \| Package \| Version \| Citation \| \| --- \| --- \| --- \| \| adjustedCurves \| 0.11.4 \| Denz et al. (2023) \| \| base \| 4.4.2 \| R Core Team (2024) \| \| broom.helpers \| 1.22.0 \| Larmarange and Sjoberg (2025) \| \| broom.mixed \| 0.2.9.7 \| Bolker and Robinson (2026) \| \| consort \| 1.2.3 \| Dayim (2026) \| \| epoxy \| 1.0.0 \| Aden-Buie (2023) \| \| flextable \| 0.9.12 \| Gohel and Skintzos (2026) \| \| ggdag \| 0.2.13 \| Barrett (2024) \| \| ggdist \| 3.3.3 \| Kay (2024); Kay (2025) \| \| ggfortify \| 0.4.19 \| Tang et al. (2016); Horikoshi and Tang (2018) \| \| ggsurvfit \| 1.2.0 \| Sjoberg et al. (2025) \| \| gt \| 1.3.0 \| Iannone et al. (2026) \| \| gtsummary \| 2.5.1 \| Sjoberg et al. (2021) \| \| knitr \| 1.51 \| Xie (2014); Xie (2015); Xie (2025) \| \| lme4 \| 2.0.1 \| Bates et al. (2015) \| \| marginaleffects \| 0.32.0 \| Arel-Bundock et al. (2024) \| \| mets \| 1.3.10 \| Scheike and Holst (2014); Scheike et al. (2014); Holst et al. (2016) \| \| mice \| 3.19.0 \| van Buuren and Groothuis-Oudshoorn (2011) \| \| naniar \| 1.1.0 \| Tierney and Cook (2023) \| \| npsurvSS \| 1.1.0 \| Yung and Liu (2024) \| \| openxlsx2 \| 1.27 \| Barbone and Garbuszus (2026) \| \| pacman \| 0.5.1 \| Rinker and Kurkiewicz (2018) \| \| patchwork \| 1.3.2 \| Pedersen (2025) \| \| quantreg \| 6.1 \| Koenker (2025) \| \| riskRegression \| 2026.3.11 \| Gerds and Kattan (2021); Gerds et al. (2026) \| \| rmarkdown \| 2.31 \| Xie et al. (2018); Xie et al. (2020); Allaire et al. (2026) \| \| scales \| 1.4.0 \| Wickham et al. (2025) \| \| stopwords \| 2.3 \| Benoit et al. (2021) \| \| survival \| 3.8.6 \| Terry M. Therneau and Patricia M. Grambsch (2000); Therneau (2026) \| \| survminer \| 0.5.2 \| Kassambara et al. (2026) \| \| tictoc \| 1.2.1 \| Izrailev (2024) \| \| tidycmprsk \| 1.1.2 \| Sjoberg and Fei (2026) \| \| tidymodels \| 1.5.0 \| Kuhn and Wickham (2020) \| \| tidytext \| 0.4.3 \| Silge and Robinson (2016) \| \| tidyverse \| 2.0.0 \| Wickham et al. (2019) \| \| wordcloud \| 2.6 \| Fellows (2018) \| |
| --- | --- | --- | --- | --- | --- | --- | --- | --- | --- | --- | --- | --- | --- | --- | --- | --- | --- | --- | --- | --- | --- | --- | --- | --- | --- | --- | --- | --- | --- | --- | --- | --- | --- | --- | --- | --- | --- | --- | --- | --- | --- | --- | --- | --- | --- | --- | --- | --- | --- | --- | --- | --- | --- | --- | --- | --- | --- | --- | --- | --- | --- | --- | --- | --- | --- | --- | --- | --- | --- | --- | --- | --- | --- | --- | --- | --- | --- | --- | --- | --- | --- | --- | --- | --- | --- | --- | --- | --- | --- | --- | --- | --- | --- | --- | --- | --- | --- | --- | --- | --- | --- | --- | --- | --- | --- | --- | --- | --- | --- | --- | --- |

## 5.5 Authorisations

Access to input datasets was pre-authorised using the *gargle* package and *googledrive*.

## 5.6 Processing functions

get_file_from_folder <- function(folder_name,
 file_pattern,
 base_folder_id) {
 tryCatch({
 # Check if the folder exists in the base directory
 folder <- googledrive::drive_ls(
 googledrive::as_id(base_folder_id),
 pattern = paste0("^", folder_name, "$")
 )

 if(nrow(folder) == 0) {
 stop(paste("Folder", folder_name, "not found"))
 }

 # Find the file in the specified folder
 target_file <- googledrive::drive_ls(
 folder$id,
 pattern = file_pattern
 )

 if(nrow(target_file) == 0) {
 stop(paste("No file matching pattern", file_pattern, "found in", folder_name))
 }

 if(nrow(target_file) > 1) {
 warning(paste("Multiple files found matching pattern. Returning first match."))
 }

 # Return file info and folder name
 return(list(
 file = target_file[1, ],
 folder_name = folder$name
 ))

 }, error = function(e) {
 stop(paste("Error finding file:", e$message))
 })
}

process_gdrive_hospital_files <- function(folder_id) {
 # Create temp directory
 temp_dir <- tempdir()
 download_dir <- file.path(temp_dir, "hospital_files")
 dir.create(download_dir, showWarnings = FALSE, recursive = TRUE)

 tryCatch({
 # Get list of .xls files from folder
 cat("Fetching file list from Drive...\n")
 drive_files <- googledrive::drive_ls(googledrive::as_id(folder_id)) |>
 dplyr::filter(stringr::str_detect(name, "\\.xls$"))

 if (nrow(drive_files) == 0) {
 warning("No .xls files found in Google Drive folder")
 return(data.frame())
 }

 cat("Found", nrow(drive_files), ".xls files. Downloading...\n")

 # Download files with progress
 purrr::iwalk(drive_files$id, ~ {
 file_name <- drive_files$name[drive_files$id == .x]
 cat("Downloading:", file_name, "\n")
 googledrive::drive_download(
 googledrive::as_id(.x),
 path = file.path(download_dir, file_name),
 overwrite = TRUE
 )
 })

 # Process the downloaded files
 cat("Processing files...\n")
 result <- list.files(
 path = download_dir,
 pattern = "\\.xls$",
 full.names = TRUE
 ) |>
 purrr::map(~ {
 tryCatch({
 readxl::read_xls(
 .,
 sheet = "Sheet1",
 range = "D1:L100",
 col_names = TRUE,
 col_types = "text",
 trim_ws = TRUE
 )
 }, error = function(e) {
 warning("Failed to read file: ", basename(.), " Error: ", e$message)
 return(NULL)
 })
 }) |>
 purrr::compact() |> # Remove NULL results
 purrr::map2(
 .y = basename(list.files(
 path = download_dir,
 pattern = "\\.xls$",
 full.names = TRUE
 )),
 ~ dplyr::mutate(.x, DataFrameName = .y)
 ) |>
 purrr::list_rbind(names_to = "DataFrameID")

 return(result)

 }, finally = {
 # Clean up temp files
 unlink(download_dir, recursive = TRUE)
 })
}

## 5.7 STROBE [8] Data Sources/Measurement:

Prior to data collection, a data dictionary was compiled and agreed by the co-authors to establish the key variables for the dataset.

## 5.8 Mastersheet Preparation

An initial mastersheet was compiled by combining the following inputs;

### 5.8.1 Hospital exports - Arthroplasty Activity

- A report was requested from the hospital which was provided in a series of xls exports.
- Filenames were extracted using *rbase* (v4.4.2) (R Core Team 2024).
- The *purrr* package (v1.2.2) (Wickham and Henry 2023) was used to loop over each file of the folder list, the data within each file were imported using the *readxl* package (v1.5.0) (Wickham and Bryan 2023).
- The dataframes were combined into one using *tidyverse* syntax (v2.0.0) (Wickham et al. 2019).

Hospital1 <- process_gdrive_hospital_files(FolderIDs$Folder1)

Fetching file list from Drive...

Found 23 .xls files. Downloading...
Downloading: April 2021.xls

Downloading: June 2022.xls

Downloading: April 2022.xls

Downloading: July 2021.xls

Downloading: June 2021.xls

Downloading: December 2021.xls

Downloading: November 2021.xls

Downloading: May 2021.xls

Downloading: February 2021.xls

Downloading: October 2021.xls

Downloading: December 2022.xls

Downloading: November 2022.xls

Downloading: October 2022.xls

Downloading: September 2022.xls

Downloading: September 2021.xls

Downloading: August 2022.xls

Downloading: July 2022.xls

Downloading: May 2022.xls

Downloading: March 2022.xls

Downloading: February 2022.xls

Downloading: January 2023.xls

Downloading: January 2022.xls

Downloading: March 2021.xls

Processing files...

The combined dataframe was reorganised with columns renamed, additional columns added and filtered based on available Unique Record Number. Date columns were reformatted using the *lubridate* package (v1.9.5) (Grolemund and Wickham 2011). Temporary identifier columns were added to provide a consistent linkage key across tables from different sources by combining URN, surgery date and surgery side.

# Tidy up combined dataframe

Hospital2 <- Hospital1 |>
 mutate(
 URN = case_when(
 !is.na(`UR NUMBER`) & is.na(`UR #`) ~ `UR NUMBER`,
 is.na(`UR NUMBER`) & !is.na(`UR #`) ~ `UR #`)
 ) |> mutate(
 Type = case_when(
 !is.na(`Prim/Rev/Bilateral`) & is.na(`PRIM/REV/PART`) & is.na(`PRIM/REV/PARTIAL`) ~ `Prim/Rev/Bilateral`,
 is.na(`Prim/Rev/Bilateral`) & !is.na(`PRIM/REV/PARTIAL`) & is.na(`PRIM/REV/PART`) ~ `PRIM/REV/PARTIAL`,
 is.na(`Prim/Rev/Bilateral`) & is.na(`PRIM/REV/PARTIAL`) & !is.na(`PRIM/REV/PART`) ~ `PRIM/REV/PART`)
 ) |> rename(
 DateSurgery = `OP DATE`
 ) |> mutate(
 DateSurgery2 = str_replace_all(DateSurgery,"\\.","-")
 ) |> mutate(
 URN2 = gsub("'","",URN),
 DateSurgery3 = lubridate::dmy(DateSurgery2)
 ) |> filter(
 !is.na(URN2)
 ) |> dplyr::select(
 -(c(
 `UR NUMBER`,
 `UR #`,
 `Prim/Rev/Bilateral`,
 `PRIM/REV/PARTIAL`,
 `PRIM/REV/PART`,
 URN,
 DateSurgery,
 DateSurgery2,
 DataFrameName))
 ) |> rename(
 DateSurgery = "DateSurgery3",
 URN = "URN2",
 Joint = "KNEE/HIP",
 Side = "SIDE",
 Brand = "COMPANY",
 Device = "SYSTEM"
 )

The surgeon-consultant identifier was recoded to a re-identifiable format and the surgery date converted to a numeric format. Temporary identifiers were added as described above.

# Recode Surgeon
Surgeon <- unique(Hospital2$CONSULTANT)

Hospital3 <- Hospital2 |> filter(
 Joint == "Knee"
 ) |> rename(
 SurgeryDate = "DateSurgery",
 PatientID = "URN",
 SurgerySide = "Side",
 SurgeryType = "Type"
 ) |> dplyr::mutate(
 SurgerySide = stringr::str_to_title(SurgerySide),
 SerialDate = round(
 as.numeric(difftime(SurgeryDate,as.Date("1899-12-30"),
 units = "days"))
 ),
 TempIDFull = paste0(PatientID,SurgerySide,SerialDate,sep = ""),
 TempIDPart = paste0(PatientID,SurgerySide,sep = ""),
 ) |> dplyr::select(
 -(c(
 Joint
 ))
 )

An initial grouping was constructed to organise the mastersheet for manual verification. Updated groupings as per description in section 4c of Protocol.

- Surgeons confirmed to be contributing to the DEPARTMENTAL REGISTRY registry and using the implant associated with the RAS - “RAS”;
- Surgeons confirmed to be contributing to the DEPARTMENTAL REGISTRY registry and *not* using the implant associated with the RAS - “Pre-RAS”;
- Surgeons confirmed *not* to be contributors to the DEPARTMENTAL REGISTRY registry, but using the implant associated with the RAS system - Group 1b (potential RAS cases)
- All other cases - “Non-RAS”

Hospital4 <- Hospital3a |> mutate(
 Group = case_when(
 (ConsultantRecode == "A" | ConsultantRecode == "B" | ConsultantRecode == "C") & Device == "Persona" ~ "RAS",
 (ConsultantRecode == "A" | ConsultantRecode == "B" | ConsultantRecode == "C") & Device != "Persona" ~ "Pre-RAS",
 (ConsultantRecode == "D" | ConsultantRecode == "E" | ConsultantRecode == "F" | ConsultantRecode == "G") & Brand == "Zimmer" & Device == "Persona" ~ "Possible-RAS",
 .default = "Non-RAS"
)
)

### 5.8.2 DEPARTMENTAL REGISTRY Snapshot and Export

A registry snapshot from DEPARTMENTAL REGISTRY was retrieved (date 16-Nov-2023) and imported as described above. The DEPARTMENTAL REGISTRY registry is a clinical quality registry embedded in the department of orthopaedics, with contributions from the physiotherapy department (Lee et al. 2020), registered on the ANZ clinical trials register ([registry trial number]) and with appropriate HREC approval (institutional HREC approval number) for the collection of patient-reported outcomes and contextual information (pathology, patient characteristics, treatment) for knee arthroplasty within the hospital. The DEPARTMENTAL REGISTRY registry snapshot was read in the method described above.

A function was generated to retrieve files using the *googledrive* package, to call on later in the analysis for processing data imports.

The snapshot for the DEPARTMENTAL REGISTRY registry was retrieved from 16 November 2023.

registry_data <- get_file_from_folder(
 folder_name = CurrentDate,
 file_pattern = "Registry data snapshot\\.xlsx$",
 base_folder_id = FolderIDs$Folder2
)

temp_file1 <- tempfile(fileext = ".xlsx")
googledrive::drive_download(
 file = registry_data$file$id,
 path = temp_file1,
 overwrite = TRUE
)

DEPARTMENTAL REGISTRYSnapshot1 <- openxlsx2::wb_to_df(
 temp_file1,
 sheet = "KneeArthritis",
 colNames = TRUE,
 detectDates = TRUE
 ) |> mutate(
 SurgerySide = stringr::str_to_title(SurgerySide),
 SerialDate = round(
 as.numeric(difftime(SurgeryDate,as.Date("1899-12-30"),
 units = "days"))
 )
) |> dplyr::select(
 !(
 c(
 Lastname,
 Firstname,
 Email
 )
 )
 ) |> unite(
 "TempIDPart",
 c(
 "PatientID",
 "SurgerySide"
 ),
 remove = FALSE,
 na.rm = TRUE,
 sep = ""
) |> unite(
 "TempIDFull",
 c(
 "PatientID",
 "SurgerySide",
 "SerialDate"
 ),
 remove = FALSE,
 na.rm = TRUE,
 sep = ""
) |> relocate(
 c(TempIDFull,TempIDPart),
 .before = PatientID
)

DEPARTMENTAL REGISTRYSoc2 <- readr::read_delim(
 file = temp_file2,
 col_names = TRUE,
 guess_max = 25,
 col_select = (-c(11,23)),
 name_repair = ~stringr::str_replace_all(str_to_title(.)," |\\\\",""),
 show_col_types = FALSE
 )

### 5.8.3 Vendor report - RAS Activity

A report from the RAS vendor was extracted and stored in an xlsx file and imported into the analysis as described above. The vendor report was read in as an input into the analysis and temporary identifiers were created as described.

VendorReport <- openxlsx2::wb_to_df(
 temp_file4,
 sheet = "Sheet1",
 colNames = TRUE,
 detectDates = TRUE
 ) |> rename(
 SurgeryDate = "Surgery Date",
 CONSULTANT = "Surgeon name",
 Description = "Surgery Description",
 PatientID = "Patient",
 SurgerySide = "Left/Right desc."
 ) |> mutate(
 SerialDate = round(
 as.numeric(difftime(SurgeryDate,as.Date("1899-12-30"),
 units = "days"))
 ),
 SurgeryType = case_when(
 grepl("revision",Description,ignore.case = TRUE) ~ "Revision",
 .default = "Primary"
 ),
 URN = purrr::map_chr(stringr::str_extract_all(PatientID, "\\d+"),~ str_c(.x[1])),
 SurgerySide = stringr::str_to_title(SurgerySide)
) |> mutate(
 URN2 = case_when(
 str_count(str_extract(URN,"\\d+")) == 5 ~ paste0("0",URN),
 str_count(str_extract(URN,"\\d+")) == 4 ~ paste0("00",URN),
 .default = URN
 )
) |> unite(
 "TempIDPart1",
 c(
 "SurgerySide",
 "SerialDate"
 ),
 remove = FALSE,
 na.rm = TRUE,
 sep = ""
) |> unite(
 "TempIDPart",
 c(
 "URN2",
 "SurgerySide"
 ),
 remove = FALSE,
 na.rm = TRUE,
 sep = ""
) |> unite(
 "TempIDFull",
 c(
 "URN2",
 "SurgerySide",
 "SerialDate"
 ),
 remove = FALSE,
 na.rm = TRUE,
 sep = ""
)

Surgeon names were recoded

VendorExclusions <- VendorReport1 |> filter(
 is.na(URN)
) |> mutate(
 Exclusion = case_when(
 str_detect(str_to_lower(Description),"canc*|revis*|demo|washout") ~ "Possible",
 str_detect(PatientID,"TBC") ~ "Possible",
 .default = "Unlikely"
 )
)

Data sources were crossmatched using one of the temporary identifier columns with partial information (URN and surgery side) as surgery date differed between sources for a proportion of cases.

Snapshot1 <- DEPARTMENTAL REGISTRYSnapshot |> dplyr::select(
 TempIDFull,
 TempIDPart,
 SerialDate,
 PatientID,
 SurgeryType,
 SurgerySide,
 ConsultantRecode,
 SurgeryDate,
 SurgeryStatus
)

CombinedInputs = bind_rows(
 list(Hospital = Hospital3a,
 DEPARTMENTAL REGISTRY = Snapshot1,
 Vendor = VendorReport1 |> anti_join(
 VendorExclusions,
 by = "TempIDPart")
 ),
 .id = "Source"
 )

MasterList <- CombinedInputs |> dplyr::select(
 PatientID,
 URN,
 TempIDFull,
 TempIDPart,
 Source,
 ConsultantRecode,
 SurgeryDate,
 SerialDate,
 SurgerySide,
 SurgeryType
 ) |> mutate(
 PatientID2 = ifelse(is.na(URN),PatientID,URN)
 ) |> group_by(
 TempIDFull
 ) |> mutate(
 Entry = row_number()
 ) |> arrange(
 Source,
 .by_group = TRUE
 )

MasterList2 <- slice_min(
 MasterList,
 Entry,
 n = 1
 ) |> filter(
 !is.na(PatientID2)
 )

### 5.8.4 Chart Review 1

A consolidated list of cases were written to a web-based file using the *googlesheets4* package (v1.1.2) (Bryan 2023) for manual verification of analysis inclusion, as well as grouping inputs (see STROBE 6). Chart review of an integrated electronic medical records system (Cerner, USA) was performed by two of the authors (JB; FL) with assistance by a third (MS). The first round of chart review confirmed inclusion criteria and grouping (defined below) for each record.

# Authenticate for sheets using the same token
gs4_auth(token = googledrive::drive_token())

googlesheets4::sheet_write(
 ss = SheetIDs$MasterList,
 data = Hospital4,
 sheet = "ConsolidList")

### 5.8.5 Chart Review 2

The initial consolidated case list was re-imported back into R and reprocessed and a new MasterSheet (v3) was re-written back into the web-based master file. Within this table additional columns were added and formulae were written to apply certain criteria to establish the inclusion and grouping columns (see below) with the following criteria;

- Revision arthroplasty | Reoperation | Not a total knee replacement | unconfirmed surgery type
- Not eligible for 3month followup (Surgery Date >= 1-Mar-2023)
- Case existence could be verified across at least two sources

The mastersheetv3 was split into separate tables for further chart review by the authors.

| Table 3: Chart Review Variables Group Pre-RAS and Non-RAS   \| **Patient Details** \| **Surgical 1** \| **Surgical 2** \| \| --- \| --- \| --- \| \| ProgressIndicator \| Surgeon \| Pathology \| \| ProgressComment \| SurgerySide \| AlignmentReferencing \| \| ComplicationForm \| Group \| SoftTissueRelease \| \| Height \| BilateralStatus \| FemurBrand \| \| Weight \| SimultaneousBilateralSurgery \| FemurFixation \| \| Comorbidities \| Surgery_ProcedureName \| FemoralSize \| \| DateOfInitialExam \| Operator \| TibiaBrand \| \| PatientID \| Approach \| TibiaFixation \| \| MockUpID \| Anaesthetic \| TibialSize \| \| SurgeryDate \| TourniquetType \| InsertThickness \| \| Sex \| Operation Start Time \| PatellaIntervention \| \| DateofBirth \| Operation Finish Time \| PatellaType \| \|  \|  \| PatellaSize \| \|  \|  \| Constraint \| |
| --- | --- | --- | --- | --- | --- | --- | --- | --- | --- | --- | --- | --- | --- | --- | --- | --- | --- | --- | --- | --- | --- | --- | --- | --- | --- | --- | --- | --- | --- | --- | --- | --- | --- | --- | --- | --- | --- | --- | --- | --- | --- | --- | --- | --- | --- |

A web-based form (QuestionPro, USA) was generated to capture key details of adverse events and complications occurring within the surgery (intraoperative) and up to 90 days postoperatively. The patient URN was used to link the information captured in the forms to the mastersheet.

### 5.8.6 Analysis - Inputs

Master tables were read in with preliminary group labelling.

#The original mastersheet should be kicking around the code blocks further up

# Authenticate for sheets using the same token
gs4_auth(token = googledrive::drive_token())

#Groups RAS (1) and Pre-RAS (2) (Preliminary labelling)
G1and2 <- read_sheet(
ss = SheetIDs$MasterList,
sheet = "ChartReviewG1-2v2",
col_names = TRUE,
col_types = "ccccnncDccDccccccccccccccccccccccccctt",
trim_ws = TRUE
)


#Groups Potential-RAS (1b) and Non-RAS (3) (Preliminary labelling)
G1band3 <- read_sheet(
ss = SheetIDs$MasterList,
sheet = "ChartReviewG1b-3",
col_names = TRUE,
col_types = "cccnncDccDccccccccccccccccccccccccttcD",
trim_ws = TRUE
)

Complication form entries were read in as one table.

# Authenticate for sheets using the same token
gs4_auth(token = googledrive::drive_token())

ComplicResponse <- read_sheet(
ss = SheetIDs$ComplicEntry,
 sheet = "ComplicROSARaw",
 col_names = TRUE,
 col_types = "cccTliicccDccccccccccccccccDccD",
 trim_ws = TRUE)

The mastersheet tables returned a combined 589 records, with 588 complication responses filed. The snapshot contained 771 records for crossmatching to import patient-reported outcomes.

### 5.8.7 Prepare dataset

The dataset was prepared for further analysis through creating new variables, recoding existing variables and merging between tables as required.

G1and2_1A <- G1and2 |> select(!(ComplicationForm)) |>
 mutate(
 GroupUpdated = case_when(
 Group == 1 ~ "RAS",
 Group == 2 ~ "Pre-RAS"
 ),
 SurgerySide = stringr::str_to_title(SurgerySide),
 BilateralStatus2 = case_when(
 BilateralStatus == "Bilateral" & SimultaneousBilateralSurgery == "Yes" ~ "Simultaneous",
 BilateralStatus == "Bilateral" & is.na(SimultaneousBilateralSurgery) ~ "Staged",
 .default = "Unilateral"
 ),
 BMI = Weight / ((Height/100)*(Height/100)),
 PotentialExclus = case_when(
 stringr::str_detect(str_to_lower(ProgressComment),"unicomp*|done|duplicate|hip|exclude|iemr" ) ~ "Yes",
 .default = "No"
 ),
 PotentialComp = case_when(
 stringr::str_detect(str_to_lower(ProgressComment),"convert*|track*|align*|rosa" ) & (Group == "1"|Group == "1b") ~ "Yes",
 .default = "No"
 ),,
 GroupCheck = case_when(
 (GroupUpdated == "RAS" & AlignmentReferencing == "Robotic")|(Group != "RAS" & AlignmentReferencing != "Robotic") ~ "Correct",
 .default = "Incorrect"
 )
 )

Surgeon names were recoded and duplicate records removed.

G1band3_1 <- G1band3 |> select(!(ComplicationForm)) |> mutate(
 GroupUpdated = case_when(
 Group == "3" ~ "Non-RAS",
 Group == "1b" ~ "Potential-RAS"
 ),
 SurgerySide = stringr::str_to_title(SurgerySide),
 BilateralStatus2 = case_when(
 BilateralStatus == "Bilateral" & SimultaneousBilateralSurgery == "Yes" ~ "Simultaneous",
 BilateralStatus == "Bilateral" & is.na(SimultaneousBilateralSurgery) ~ "Staged",
 .default = "Unilateral"),
 BMI = Weight / ((Height/100)*(Height/100)),
 PotentialExclus = case_when(
 stringr::str_detect(str_to_lower(ProgressComment),"unicomp*|done|duplicate|hip|exclude|iemr" ) ~ "Yes",
 stringr::str_detect(str_to_lower(ProgressComment),"lcck|depuy|lcs|rhk" ) & (Group == "1"|Group == "1b") ~ "Yes",
 .default = "No"
 ),
 PotentialComp = case_when(
 stringr::str_detect(str_to_lower(ProgressComment),"convert*|track*|align*|rosa" ) & (Group == "1"|Group == "1b") ~ "Yes",
 .default = "No"
 ),
 GroupCheck = case_when(
 (GroupUpdated == "Potential-RAS" & AlignmentReferencing == "Robotic")|(Group != "Potential-RAS" & (AlignmentReferencing != "Robotic")|is.na(AlignmentReferencing)) ~ "Correct",
 .default = "Incorrect"
 ),
 AgeAtSurgery = as.numeric(as.duration(interval(ymd(DOB), ymd(SurgeryDate))),"years"),
 Sex = case_when(
 Sex == "M" ~ "Male",
 Sex == "F" ~ "Female",
 .default = NA_character_
 )
 )

Individual input tables were joined into a MasterSheet and additional variables created as needed.

MasterSheet1 <- bind_rows(
 G1band3_1,
 G1and2_1B
) |> dplyr::select(
 -Process
) |> mutate(
 SerialDate = round(
 as.numeric(difftime(SurgeryDate,as.Date("1899-12-30"),
 units = "days"))),
 across(where(is.character) & !c(MockUpID,GroupUpdated), ~str_to_title(.)), #this line turns all side labels in "MockUpID" into lower case; no longer matches to TempIDFull in MasterList
 PatientID2 = case_when(
 str_count(PatientID) == 5 ~ paste0("0",PatientID),
 str_count(PatientID) == 4 ~ paste0("00",PatientID),
 .default = PatientID
 )
 ) |> unite(
 "TempIDFull",
 c("PatientID2",
 "SurgerySide",
 "SerialDate"
 ),
 remove = FALSE,
 na.rm = TRUE,
 sep = ""
 ) |> unite(
 "TempIDPart",
 c("PatientID2",
 "SurgerySide"
 ),
 remove = FALSE,
 na.rm = TRUE,
 sep = ""
 ) |> relocate(
 PotentialExclus
 )

Identifier columns were reviewed for coherence.

# Step 1: Create a new column that flags mismatches
MasterSheetCheck <- MasterSheet1 |>
 mutate(id_mismatch = MockUpID != TempIDFull)

# Step 2: View all rows with mismatches
mismatched_rows <- MasterSheetCheck |>
 filter(id_mismatch == TRUE)

# Step 3: Print the number of mismatches
print(paste("Number of mismatches:", nrow(mismatched_rows)))

[1] "Number of mismatches: 65"

# Step 4: View the mismatched rows
print(mismatched_rows)

# A tibble: 65 × 50
 PotentialExclus ProgressIndicator ProgressComment Height Weight Comorbidities
 <chr> <chr> <chr> <dbl> <dbl> <chr>
 1 No Done Ms <NA> 165 63.8 Nil
 2 No Done Ms <NA> 172 88.6 Ihd
 3 No Done Ms <NA> 178 95 Nil
 4 No Done Ms <NA> 156 70 Nil
 5 No Done Ms <NA> 174 98 Previous Ca
 6 No Done Ms <NA> 172 72 Nil
 7 No Done Ms <NA> 169 97 Previous Ca
 8 No Done Ms <NA> 158 62.5 Nil
 9 No Done Jb ?St Release - … 160 86 Nil
10 No Done Ms <NA> 168 95 Nil
# ℹ 55 more rows
# ℹ 44 more variables: DateOfInitialExam <date>, PatientID <chr>,
# MockUpID <chr>, SurgeryDate <date>, Surgeon <chr>, TempIDFull <chr>,
# TempIDPart <chr>, SurgerySide <chr>, Group <chr>, BilateralStatus <chr>,
# SimultaneousBilateralSurgery <chr>, Surgery_ProcedureName <chr>,
# Operator <chr>, Approach <chr>, Anaesthetic <chr>, TourniquetType <chr>,
# Pathology <chr>, AlignmentReferencing <chr>, SoftTissueRelease <chr>, …

# Optional: If you want to see only the identifier columns and a few others
mismatched_ids <- mismatched_rows |>
 select(MockUpID, TempIDFull)
print(mismatched_ids)

# A tibble: 65 × 2
 MockUpID TempIDFull
 <chr> <chr>
 1 12358Left44273 012358Left44273
 2 12235Right44279 012235Right44279
 3 74223Left44447 074223Left44447
 4 2045743Left44293 2045743Left44658
 5 70840Left44727 070840Left44727
 6 7029336LEFT44784 7029336Left44784
 7 29992Right44784 029992Right44784
 8 13906Right44805 013906Right44805
 9 38179Right44811 038179Right44811
10 85725Right44833 085725Right44833
# ℹ 55 more rows

The mastersheet was checked for duplicate records.

# Check for duplicates

MasterDuplicates <-MasterSheet1 |>
 summarise(
 n = n(),
 .by = "TempIDPart"
 ) |> filter(
 n > 1
 )

Table3 <- tbl_summary(
 MasterSheet1 |> dplyr::select(
 GroupUpdated,
 Surgeon,
 SurgeryDate,
 SurgerySide,
 AlignmentReferencing
 ),
 by = GroupUpdated,
 statistic = list(
 SurgeryDate = "{min} - {max}"
 )
) |> add_overall()

as_flex_table(Table3) |> flextable::set_table_properties(
 layout = "autofit",
 width = 0.15,
 align = "center",
 opts_word = list(
 split = TRUE
 )
 )

| Table 4: Summary of input variables used to determine group labelling.   \| **Characteristic** \| **Overall**  N = 588^1^ \| **Non-RAS**  N = 125^1^ \| **Potential-RAS**  N = 127^1^ \| **Pre-RAS**  N = 164^1^ \| **RAS**  N = 172^1^ \| \| --- \| --- \| --- \| --- \| --- \| --- \| \| Surgeon \|  \|  \|  \|  \|  \| \| A \| 58 (9.9%) \| 0 (0%) \| 0 (0%) \| 48 (29%) \| 10 (5.8%) \| \| B \| 106 (18%) \| 0 (0%) \| 0 (0%) \| 49 (30%) \| 57 (33%) \| \| C \| 172 (29%) \| 0 (0%) \| 0 (0%) \| 67 (41%) \| 105 (61%) \| \| D \| 46 (7.8%) \| 45 (36%) \| 1 (0.8%) \| 0 (0%) \| 0 (0%) \| \| E \| 40 (6.8%) \| 7 (5.6%) \| 33 (26%) \| 0 (0%) \| 0 (0%) \| \| F \| 29 (4.9%) \| 3 (2.4%) \| 26 (20%) \| 0 (0%) \| 0 (0%) \| \| G \| 137 (23%) \| 70 (56%) \| 67 (53%) \| 0 (0%) \| 0 (0%) \| \| SurgeryDate \| 2017-09-15 - 2023-02-07 \| 2021-02-02 - 2023-01-18 \| 2021-02-08 - 2023-01-30 \| 2017-09-15 - 2021-02-17 \| 2021-02-19 - 2023-02-07 \| \| SurgerySide \|  \|  \|  \|  \|  \| \| Left \| 291 (49%) \| 54 (43%) \| 62 (49%) \| 81 (49%) \| 94 (55%) \| \| Right \| 297 (51%) \| 71 (57%) \| 65 (51%) \| 83 (51%) \| 78 (45%) \| \| AlignmentReferencing \|  \|  \|  \|  \|  \| \| Im Femur/Em Tibia \| 116 (21%) \| 92 (76%) \| 1 (0.8%) \| 22 (14%) \| 1 (0.6%) \| \| Navigation \| 187 (33%) \| 29 (24%) \| 14 (11%) \| 131 (86%) \| 13 (8.0%) \| \| Patient Specific Guides \| 1 (0.2%) \| 0 (0%) \| 0 (0%) \| 0 (0%) \| 1 (0.6%) \| \| Robotic \| 255 (46%) \| 0 (0%) \| 108 (88%) \| 0 (0%) \| 147 (91%) \| \| Unknown \| 29 \| 4 \| 4 \| 11 \| 10 \| \| ^1^n (%); Min - Max \| \| \| \| \| \| |
| --- | --- | --- | --- | --- | --- | --- | --- | --- | --- | --- | --- | --- | --- | --- | --- | --- | --- | --- | --- | --- | --- | --- | --- | --- | --- | --- | --- | --- | --- | --- | --- | --- | --- | --- | --- | --- | --- | --- | --- | --- | --- | --- | --- | --- | --- | --- | --- | --- | --- | --- | --- | --- | --- | --- | --- | --- | --- | --- | --- | --- | --- | --- | --- | --- | --- | --- | --- | --- | --- | --- | --- | --- | --- | --- | --- | --- | --- | --- | --- | --- | --- | --- | --- | --- | --- | --- | --- | --- | --- | --- | --- | --- | --- | --- | --- | --- | --- | --- | --- | --- | --- | --- | --- | --- | --- | --- | --- | --- | --- | --- | --- | --- | --- | --- | --- | --- | --- | --- | --- | --- |

Following chart review, inconsistencies in group labels were resolved by retrieving the alignment reference method and comparing the presence of the record in the vendor report.

SerialG1Start <- as.numeric(difftime(as.Date("2021-02-19"),as.Date("1899-12-30")))

VendorExclusions2 <- VendorExclusions |> filter(
 Exclusion == "Possible"
)

MasterSheet2 <- MasterSheet1 |> filter(
 PotentialExclus != "Yes"
 ) |> mutate(# fix a couple of uncertain groupings
 AlignmentReferencing = case_when(
 MockUpID == "2045744Left44895" ~ "Robotic",
 MockUpID == "267722left44743" ~ "Robotic",
 .default = AlignmentReferencing
 )
 ) |> mutate(
 GroupCorrected = case_when(
 (AlignmentReferencing == "Robotic") | (is.na(AlignmentReferencing) & (TempIDPart %in% VendorReport$TempIDPart)) ~ "RAS",
GroupUpdated == "Pre-RAS" | (GroupUpdated == "RAS" & !is.na(AlignmentReferencing) & AlignmentReferencing != "Robotic") ~ "Pre-RAS",
GroupUpdated == "RAS" & is.na(AlignmentReferencing) & !(TempIDPart %in% VendorReport$TempIDPart) ~ "Pre-RAS",
GroupUpdated == "Non-RAS" | (GroupUpdated == "Potential-RAS" & !is.na(AlignmentReferencing) & AlignmentReferencing != "Robotic") ~ "Non-RAS",
 .default = "Non-RAS"
 )
) |> arrange(
 SurgeryDate
 ) |> relocate(
 GroupCorrected,
 .after = Group
 )

MasterSheetExclusions <- MasterSheet1 |> filter(
 PotentialExclus == "Yes"
)

Unsure <- MasterSheet2 |> filter(
 is.na(AlignmentReferencing)
)

The complication response table was filtered for entries missing a patient record identifier.

#Had to add a filter on missing PatientIDs (URNs) in the ComplicResponse table as the mutate function str_count would not work with NAs passed as input.
ComplicResponse2 <- ComplicResponse |> filter(
 !is.na(URN)
 )

A list of records in the MasterSheet missing at least one complication form entry were extracted for manual review.

MissingComplic <- MasterSheet2 |> filter(
 !(PatientID %in% ComplicResponse2$URN)
)

There are currently 8 treatment records that have not got any complication entries against them in the mastersheet

The DEPARTMENTAL REGISTRY snapshot was used to update demographic data (age, sex, body mass index) for cases in the MasterSheet that were not retrieved during chart review.

# Create MockUpID; concatenate PatientID, Side and SurgeryDate

DEPARTMENTAL REGISTRYSnapshot1 <- DEPARTMENTAL REGISTRYSnapshot |> mutate(
 SurgerySide = stringr::str_to_title(SurgerySide)
) |> unite(
 "TempIDFull",
 c(PatientID,SurgerySide,SerialDate),
 remove = FALSE,
 na.rm = FALSE,
 sep = ""
 ) |> unite(
 "TempIDPart",
 c(PatientID,SurgerySide),
 remove = FALSE,
 na.rm = FALSE,
 sep = ""
 )

MasterSheet3 <- MasterSheet2 |> left_join(
 DEPARTMENTAL REGISTRYSnapshot1 |> filter(
 SurgeryType == "Primary"
 ) |> dplyr::select(
 TempIDPart,
 AgeAtSurgery,
 Gender,
 BMI
 ) |> rename(
 Sex = "Gender"
 ),
 join_by("TempIDPart" == "TempIDPart")
 ) |> mutate(
 Sex = ifelse(
 is.na(Sex.x),Sex.y,Sex.x
 ),
 AgeAtSurgery = ifelse(
 is.na(AgeAtSurgery.x),
 AgeAtSurgery.y,
 AgeAtSurgery.x
 ),
 BMI = ifelse(
 is.na(BMI.x),BMI.y,BMI.x
 )
 ) |> dplyr::select(
 -(ends_with(".x")
 )
 ) |> dplyr::select(
 -(ends_with(".y")
 )
 )

The reduction in missing data was confirmed before and after the table merge to verify the successful table join using the *naniar* package (v1.1.0) (Tierney and Cook 2023).

FigureMiss1 <- gg_miss_var(MasterSheet2 |> dplyr::select(
 AgeAtSurgery,
 Sex
),
show_pct = TRUE
) + theme_bw()

FigureMiss2 <- gg_miss_var(MasterSheet3 |> dplyr::select(
 AgeAtSurgery,
 Sex
),
show_pct = TRUE
) + theme_bw()

FigureMiss1 / FigureMiss2

| 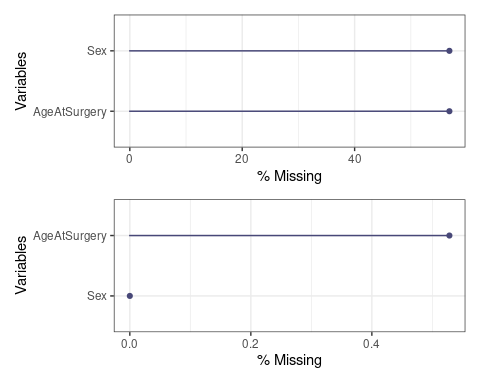  Figure 2: Missingness in AgeatSurgery and Sex before (top) and after (bottom) merge between MasterSheet and DEPARTMENTAL REGISTRY Snapshot. |
| --- |

## 5.9 STROBE [6] Participants:

*Critique the definition of the study population, including inclusion and exclusion criteria.*

### 5.9.1 IDEAL Stage2b [2]: Inclusion-criteria

*Observational studies should be diagnosis-based, rather than procedure-based whenever possible*

The criteria for this study was procedure based - examining only cases that received a total knee replacement within the department. Inclusion criteria was confirmed by establishing the following during *chart review* (rounds 1 and 2);

- Date of Surgery < **1-Mar-2023**
- Surgery type (arthroplasty)

Exclusion criteria included;

- Revision | Reoperation | Procedure other than knee arthroplasty | Arthroplasty type (revision vs primary) could not be confirmed

## 5.10 STROBE [7] Variables:

*Evaluate the choice of exposure, outcome, and confounding variables.*

### 5.10.1 Exposure variables

Group labels

Grouping information was confirmed by chart review for the following additional variables;

- Consultant surgeon
- Date of Surgery
- Alignment delivery (robotic, navigation, manual instrumentation)
- Prosthesis system implanted

Following chart review 1, further processing was performed to confirm the grouping for each record included in the analysis.

- Create corrected group label (1, 2, 3)
- Identify DEPARTMENTAL REGISTRY and non-DEPARTMENTAL REGISTRY cases (establish expectation of PROMs availability)
- Assess availabilty of complications data

The initial state of the grouping variables was tabled using gtsummary to ensure that the patterns were as expected.

Table4 <- tbl_summary(
 MasterSheet3 |> dplyr::select(
 GroupCorrected,
 Surgeon,
 SurgeryDate,
 SurgerySide,
 AlignmentReferencing,
 AgeAtSurgery,
 Sex,
 SurgerySide
 ),
 by = GroupCorrected,
 statistic = list(SurgeryDate ~ "{min} - {max}")
) |> add_overall()

knitr::knit_print(Table4)

| Table 5: Summary of grouping inputs separated by estimated grouping.   \| **Characteristic** \| **Overall** N = 568*^1^* \| **Non-RAS** N = 137*^1^* \| **Pre-RAS** N = 173*^1^* \| **RAS** N = 258*^1^* \| \| --- \| --- \| --- \| --- \| --- \| \| Surgeon \|  \|  \|  \|  \| \| A \| 55 (9.7%) \| 0 (0%) \| 45 (26%) \| 10 (3.9%) \| \| B \| 98 (17%) \| 0 (0%) \| 54 (31%) \| 44 (17%) \| \| C \| 170 (30%) \| 0 (0%) \| 74 (43%) \| 96 (37%) \| \| D \| 46 (8.1%) \| 46 (34%) \| 0 (0%) \| 0 (0%) \| \| E \| 38 (6.7%) \| 7 (5.1%) \| 0 (0%) \| 31 (12%) \| \| F \| 26 (4.6%) \| 1 (0.7%) \| 0 (0%) \| 25 (9.7%) \| \| G \| 135 (24%) \| 83 (61%) \| 0 (0%) \| 52 (20%) \| \| SurgeryDate \| 2017-09-15 - 2023-02-07 \| 2021-02-02 - 2023-01-30 \| 2017-09-15 - 2023-02-07 \| 2021-02-19 - 2023-01-19 \| \| SurgerySide \|  \|  \|  \|  \| \| Left \| 278 (49%) \| 59 (43%) \| 88 (51%) \| 131 (51%) \| \| Right \| 290 (51%) \| 78 (57%) \| 85 (49%) \| 127 (49%) \| \| AlignmentReferencing \|  \|  \|  \|  \| \| Im Femur/Em Tibia \| 116 (21%) \| 93 (68%) \| 23 (14%) \| 0 (0%) \| \| Navigation \| 183 (33%) \| 43 (32%) \| 140 (86%) \| 0 (0%) \| \| Robotic \| 254 (46%) \| 0 (0%) \| 0 (0%) \| 254 (100%) \| \| Unknown \| 15 \| 1 \| 10 \| 4 \| \| AgeAtSurgery \| 69 (62, 76) \| 70 (63, 77) \| 68 (61, 75) \| 69 (64, 76) \| \| Unknown \| 3 \| 0 \| 0 \| 3 \| \| Sex \|  \|  \|  \|  \| \| Female \| 322 (57%) \| 88 (64%) \| 93 (54%) \| 141 (55%) \| \| Male \| 246 (43%) \| 49 (36%) \| 80 (46%) \| 117 (45%) \| \| *^1^*n (%); Min - Max; Median (Q1, Q3) \| \| \| \| \| |
| --- | --- | --- | --- | --- | --- | --- | --- | --- | --- | --- | --- | --- | --- | --- | --- | --- | --- | --- | --- | --- | --- | --- | --- | --- | --- | --- | --- | --- | --- | --- | --- | --- | --- | --- | --- | --- | --- | --- | --- | --- | --- | --- | --- | --- | --- | --- | --- | --- | --- | --- | --- | --- | --- | --- | --- | --- | --- | --- | --- | --- | --- | --- | --- | --- | --- | --- | --- | --- | --- | --- | --- | --- | --- | --- | --- | --- | --- | --- | --- | --- | --- | --- | --- | --- | --- | --- | --- | --- | --- | --- | --- | --- | --- | --- | --- | --- | --- | --- | --- | --- | --- | --- | --- | --- | --- | --- | --- | --- | --- | --- | --- | --- | --- | --- | --- | --- | --- | --- | --- | --- |

The surgical fields were adjusted one at a time using a combination of *tidyverse* (v2.0.0) (Wickham et al. 2019) and *stringr* (v1.6.0) (Wickham 2023) packages. The initial state of the surgical fields was tabled using *gtsummary.*

Table5 <- tbl_summary(
 MasterSheet3 |> dplyr::select(
 GroupCorrected,
 BilateralStatus2,
 Surgery_ProcedureName:FemurFixation,
 TibiaFixation,
 PatellaIntervention,
 Constraint
 ),
 by = GroupCorrected
) |> add_overall()

as_flex_table(Table5) |> flextable::set_table_properties(
 layout = "autofit",
 width = 0.15,
 align = "center",
 opts_word = list(
 split = TRUE
 )
 )

| Table 6: Summary of unadjusted surgical fields separated by estimated grouping.   \| **Characteristic** \| **Overall**  N = 568^1^ \| **Non-RAS**  N = 137^1^ \| **Pre-RAS**  N = 173^1^ \| **RAS**  N = 258^1^ \| \| --- \| --- \| --- \| --- \| --- \| \| BilateralStatus2 \|  \|  \|  \|  \| \| Simultaneous \| 53 (9.3%) \| 8 (5.8%) \| 20 (12%) \| 25 (9.7%) \| \| Staged \| 149 (26%) \| 46 (34%) \| 49 (28%) \| 54 (21%) \| \| Unilateral \| 366 (64%) \| 83 (61%) \| 104 (60%) \| 179 (69%) \| \| Surgery_ProcedureName \|  \|  \|  \|  \| \| Kap1 - Primary Total Arthroplasty (Unilateral) \| 376 (68%) \| 84 (61%) \| 115 (68%) \| 177 (72%) \| \| Kap2 - Primary Total Arthroplasty (Bilateral) \| 63 (11%) \| 12 (8.8%) \| 20 (12%) \| 31 (13%) \| \| Kap2.2 - Primary Total Arthroplasty (Subsequent Bilateral) \| 113 (20%) \| 41 (30%) \| 33 (20%) \| 39 (16%) \| \| Unknown \| 16 \| 0 \| 5 \| 11 \| \| Operator \|  \|  \|  \|  \| \| Fellow \| 3 (0.5%) \| 0 (0%) \| 3 (1.8%) \| 0 (0%) \| \| Junior Resident/Registrar \| 2 (0.4%) \| 0 (0%) \| 1 (0.6%) \| 1 (0.4%) \| \| Senior Resident/Registrar \| 156 (28%) \| 58 (42%) \| 43 (25%) \| 55 (21%) \| \| Surgeon \| 402 (71%) \| 79 (58%) \| 122 (72%) \| 201 (78%) \| \| Unknown \| 5 \| 0 \| 4 \| 1 \| \| Approach \|  \|  \|  \|  \| \| Lat Parapatellar \| 28 (5.0%) \| 0 (0%) \| 12 (7.1%) \| 16 (6.2%) \| \| Lateral Parapatellar \| 2 (0.4%) \| 0 (0%) \| 0 (0%) \| 2 (0.8%) \| \| Med Parapatellar \| 521 (93%) \| 127 (93%) \| 155 (92%) \| 239 (93%) \| \| Med Parapatellar, Quads Snip \| 1 (0.2%) \| 0 (0%) \| 1 (0.6%) \| 0 (0%) \| \| Medial Parapatellar \| 10 (1.8%) \| 10 (7.3%) \| 0 (0%) \| 0 (0%) \| \| Other \| 1 (0.2%) \| 0 (0%) \| 1 (0.6%) \| 0 (0%) \| \| Unknown \| 5 \| 0 \| 4 \| 1 \| \| Anaesthetic \|  \|  \|  \|  \| \| Epidural \| 5 (0.9%) \| 0 (0%) \| 4 (2.4%) \| 1 (0.4%) \| \| Epidural, Sedation \| 1 (0.2%) \| 0 (0%) \| 1 (0.6%) \| 0 (0%) \| \| Ga \| 9 (1.6%) \| 8 (5.8%) \| 0 (0%) \| 1 (0.4%) \| \| General \| 91 (16%) \| 20 (15%) \| 27 (16%) \| 44 (17%) \| \| General, Epidural \| 6 (1.1%) \| 4 (2.9%) \| 0 (0%) \| 2 (0.8%) \| \| General, Regional Block \| 5 (0.9%) \| 2 (1.5%) \| 3 (1.8%) \| 0 (0%) \| \| General, Regional Block, Spinal \| 2 (0.4%) \| 2 (1.5%) \| 0 (0%) \| 0 (0%) \| \| General, Spinal \| 74 (13%) \| 5 (3.6%) \| 27 (16%) \| 42 (16%) \| \| General, Spinal, Epidural \| 1 (0.2%) \| 0 (0%) \| 0 (0%) \| 1 (0.4%) \| \| General, Spinal, Sedation \| 1 (0.2%) \| 0 (0%) \| 1 (0.6%) \| 0 (0%) \| \| Regional Block, Epidural \| 1 (0.2%) \| 0 (0%) \| 1 (0.6%) \| 0 (0%) \| \| Regional Block, Spinal \| 5 (0.9%) \| 1 (0.7%) \| 4 (2.4%) \| 0 (0%) \| \| Regional Block, Spinal, Sedation \| 3 (0.5%) \| 0 (0%) \| 3 (1.8%) \| 0 (0%) \| \| Spinal \| 230 (41%) \| 57 (42%) \| 59 (35%) \| 114 (44%) \| \| Spinal + Sedation \| 1 (0.2%) \| 1 (0.7%) \| 0 (0%) \| 0 (0%) \| \| Spinal, Epidural \| 7 (1.2%) \| 0 (0%) \| 2 (1.2%) \| 5 (1.9%) \| \| Spinal, Ga \| 2 (0.4%) \| 2 (1.5%) \| 0 (0%) \| 0 (0%) \| \| Spinal, General \| 5 (0.9%) \| 2 (1.5%) \| 0 (0%) \| 3 (1.2%) \| \| Spinal, Regional, General \| 1 (0.2%) \| 1 (0.7%) \| 0 (0%) \| 0 (0%) \| \| Spinal, Sedation \| 112 (20%) \| 31 (23%) \| 37 (22%) \| 44 (17%) \| \| Spnial, General \| 1 (0.2%) \| 1 (0.7%) \| 0 (0%) \| 0 (0%) \| \| Unknown \| 5 \| 0 \| 4 \| 1 \| \| TourniquetType \|  \|  \|  \|  \| \| Intemittent \| 2 (0.4%) \| 2 (1.5%) \| 0 (0%) \| 0 (0%) \| \| Intermittent \| 43 (7.8%) \| 11 (8.0%) \| 6 (3.8%) \| 26 (10%) \| \| Not Used \| 88 (16%) \| 9 (6.6%) \| 43 (27%) \| 36 (14%) \| \| Released After Closure \| 46 (8.4%) \| 1 (0.7%) \| 26 (17%) \| 19 (7.5%) \| \| Released Before Closure \| 369 (67%) \| 114 (83%) \| 82 (52%) \| 173 (68%) \| \| Unknown \| 20 \| 0 \| 16 \| 4 \| \| Pathology \|  \|  \|  \|  \| \| Inflammatory Arthritis \| 2 (0.4%) \| 0 (0%) \| 1 (0.6%) \| 1 (0.4%) \| \| Osteoarthritis \| 555 (99%) \| 136 (99%) \| 165 (98%) \| 254 (99%) \| \| Osteonecrosis \| 1 (0.2%) \| 0 (0%) \| 1 (0.6%) \| 0 (0%) \| \| Trauma \| 4 (0.7%) \| 1 (0.7%) \| 2 (1.2%) \| 1 (0.4%) \| \| Unknown \| 6 \| 0 \| 4 \| 2 \| \| AlignmentReferencing \|  \|  \|  \|  \| \| Im Femur/Em Tibia \| 116 (21%) \| 93 (68%) \| 23 (14%) \| 0 (0%) \| \| Navigation \| 183 (33%) \| 43 (32%) \| 140 (86%) \| 0 (0%) \| \| Robotic \| 254 (46%) \| 0 (0%) \| 0 (0%) \| 254 (100%) \| \| Unknown \| 15 \| 1 \| 10 \| 4 \| \| SoftTissueRelease \|  \|  \|  \|  \| \| Combined Release/Tighten \| 1 (0.2%) \| 1 (0.7%) \| 0 (0%) \| 0 (0%) \| \| Extensive Lateral \| 10 (1.8%) \| 0 (0%) \| 4 (2.4%) \| 6 (2.4%) \| \| Extensive Medial \| 27 (4.9%) \| 1 (0.7%) \| 12 (7.3%) \| 14 (5.5%) \| \| Minimal Lateral \| 29 (5.2%) \| 0 (0%) \| 21 (13%) \| 8 (3.2%) \| \| Minimal Medial \| 403 (73%) \| 124 (91%) \| 74 (45%) \| 205 (81%) \| \| Minimal Medial, Itb Pie Crust \| 4 (0.7%) \| 4 (2.9%) \| 0 (0%) \| 0 (0%) \| \| None \| 75 (14%) \| 6 (4.4%) \| 49 (30%) \| 20 (7.9%) \| \| Other \| 5 (0.9%) \| 0 (0%) \| 5 (3.0%) \| 0 (0%) \| \| Unknown \| 14 \| 1 \| 8 \| 5 \| \| FemurBrand \|  \|  \|  \|  \| \| Attune Cr \| 50 (9.1%) \| 50 (36%) \| 0 (0%) \| 0 (0%) \| \| Attune Ps \| 1 (0.2%) \| 1 (0.7%) \| 0 (0%) \| 0 (0%) \| \| Nexgen Cr Porous \| 3 (0.5%) \| 0 (0%) \| 3 (1.9%) \| 0 (0%) \| \| Nexgen Cr-Flex Gs Porous Left \| 8 (1.5%) \| 0 (0%) \| 8 (5.1%) \| 0 (0%) \| \| Nexgen Cr-Flex Gs Porous Right \| 13 (2.4%) \| 0 (0%) \| 13 (8.3%) \| 0 (0%) \| \| Nexgen Cr-Flex Gs Precoat Left \| 5 (0.9%) \| 0 (0%) \| 5 (3.2%) \| 0 (0%) \| \| Nexgen Cr-Flex Hatcp Por. Left \| 1 (0.2%) \| 0 (0%) \| 1 (0.6%) \| 0 (0%) \| \| Nexgen Cr-Flex Hatcp Por. Right \| 2 (0.4%) \| 0 (0%) \| 2 (1.3%) \| 0 (0%) \| \| Nexgen Cr-Flex Precoat Left \| 1 (0.2%) \| 0 (0%) \| 1 (0.6%) \| 0 (0%) \| \| Nexgen Cr-Flex Precoat Right \| 3 (0.5%) \| 0 (0%) \| 3 (1.9%) \| 0 (0%) \| \| Persona Cemented Ps \| 1 (0.2%) \| 0 (0%) \| 0 (0%) \| 1 (0.4%) \| \| Persona Cr Narrow \| 3 (0.5%) \| 1 (0.7%) \| 0 (0%) \| 2 (0.8%) \| \| Persona Cr Narrow Cemented \| 70 (13%) \| 1 (0.7%) \| 25 (16%) \| 44 (17%) \| \| Persona Cr Standard \| 15 (2.7%) \| 4 (2.9%) \| 0 (0%) \| 11 (4.3%) \| \| Persona Cr Standard Cemented \| 276 (50%) \| 8 (5.8%) \| 96 (61%) \| 172 (67%) \| \| Persona Cr Standard Uncemented \| 20 (3.6%) \| 0 (0%) \| 0 (0%) \| 20 (7.8%) \| \| Persona Mc Narrow \| 1 (0.2%) \| 0 (0%) \| 0 (0%) \| 1 (0.4%) \| \| Persona Mc Standard \| 1 (0.2%) \| 0 (0%) \| 0 (0%) \| 1 (0.4%) \| \| Persona Ps Narrow Cemented \| 1 (0.2%) \| 0 (0%) \| 0 (0%) \| 1 (0.4%) \| \| Persona Ps Standard \| 2 (0.4%) \| 0 (0%) \| 0 (0%) \| 2 (0.8%) \| \| Persona Revision \| 1 (0.2%) \| 0 (0%) \| 0 (0%) \| 1 (0.4%) \| \| Persona Trabecular Metal Porous \| 1 (0.2%) \| 0 (0%) \| 0 (0%) \| 1 (0.4%) \| \| S&N Genesis 2 \| 18 (3.3%) \| 18 (13%) \| 0 (0%) \| 0 (0%) \| \| Triathlon Cr Beaded \| 1 (0.2%) \| 1 (0.7%) \| 0 (0%) \| 0 (0%) \| \| Triathlon Cr Cemented \| 47 (8.5%) \| 47 (34%) \| 0 (0%) \| 0 (0%) \| \| Triathlon Ps Cemented \| 1 (0.2%) \| 1 (0.7%) \| 0 (0%) \| 0 (0%) \| \| Triathlon Ts \| 1 (0.2%) \| 1 (0.7%) \| 0 (0%) \| 0 (0%) \| \| Zimmer Biomet Nexgen \| 2 (0.4%) \| 2 (1.5%) \| 0 (0%) \| 0 (0%) \| \| Zimmer Rhk \| 2 (0.4%) \| 2 (1.5%) \| 0 (0%) \| 0 (0%) \| \| Unknown \| 17 \| 0 \| 16 \| 1 \| \| FemurFixation \|  \|  \|  \|  \| \| Cement \| 336 (61%) \| 83 (61%) \| 109 (69%) \| 144 (56%) \| \| Cemented \| 11 (2.0%) \| 1 (0.7%) \| 0 (0%) \| 10 (3.9%) \| \| Cementless \| 182 (33%) \| 52 (38%) \| 49 (31%) \| 81 (32%) \| \| Uncemented \| 21 (3.8%) \| 0 (0%) \| 0 (0%) \| 21 (8.2%) \| \| Unknown \| 18 \| 1 \| 15 \| 2 \| \| TibiaFixation \|  \|  \|  \|  \| \| Cement \| 437 (79%) \| 87 (64%) \| 144 (89%) \| 206 (81%) \| \| Cemented \| 22 (4.0%) \| 5 (3.6%) \| 0 (0%) \| 17 (6.7%) \| \| Cementless \| 83 (15%) \| 45 (33%) \| 17 (11%) \| 21 (8.3%) \| \| Uncemented \| 9 (1.6%) \| 0 (0%) \| 0 (0%) \| 9 (3.6%) \| \| Unknown \| 17 \| 0 \| 12 \| 5 \| \| PatellaIntervention \|  \|  \|  \|  \| \| Not Resurfaced \| 133 (24%) \| 57 (42%) \| 44 (26%) \| 32 (12%) \| \| Other \| 1 (0.2%) \| 0 (0%) \| 1 (0.6%) \| 0 (0%) \| \| Resurfaced \| 429 (76%) \| 80 (58%) \| 124 (73%) \| 225 (88%) \| \| Unknown \| 5 \| 0 \| 4 \| 1 \| \| Constraint \|  \|  \|  \|  \| \| Constrained Condylar \| 3 (0.5%) \| 0 (0%) \| 2 (1.3%) \| 1 (0.4%) \| \| Cruciate Retaining \| 465 (84%) \| 118 (86%) \| 126 (79%) \| 221 (86%) \| \| Cruciate Sacrificing \| 1 (0.2%) \| 0 (0%) \| 1 (0.6%) \| 0 (0%) \| \| Cruciate Stabilised \| 5 (0.9%) \| 5 (3.6%) \| 0 (0%) \| 0 (0%) \| \| Cruciate Substituing \| 1 (0.2%) \| 0 (0%) \| 0 (0%) \| 1 (0.4%) \| \| Cruciate Substituting \| 2 (0.4%) \| 2 (1.5%) \| 0 (0%) \| 0 (0%) \| \| Crutiate Retaining \| 1 (0.2%) \| 0 (0%) \| 0 (0%) \| 1 (0.4%) \| \| Deep Dish \| 1 (0.2%) \| 0 (0%) \| 1 (0.6%) \| 0 (0%) \| \| Lcck \| 2 (0.4%) \| 2 (1.5%) \| 0 (0%) \| 0 (0%) \| \| Mc \| 2 (0.4%) \| 0 (0%) \| 0 (0%) \| 2 (0.8%) \| \| Medial Pivot \| 34 (6.2%) \| 0 (0%) \| 14 (8.8%) \| 20 (7.8%) \| \| Mobile Bearing \| 5 (0.9%) \| 5 (3.6%) \| 0 (0%) \| 0 (0%) \| \| Other \| 8 (1.4%) \| 0 (0%) \| 5 (3.1%) \| 3 (1.2%) \| \| Posterior Stabilised \| 20 (3.6%) \| 3 (2.2%) \| 10 (6.3%) \| 7 (2.7%) \| \| Rhk Hinge \| 1 (0.2%) \| 1 (0.7%) \| 0 (0%) \| 0 (0%) \| \| Ts Poly \| 1 (0.2%) \| 1 (0.7%) \| 0 (0%) \| 0 (0%) \| \| Unknown \| 16 \| 0 \| 14 \| 2 \| \| ^1^n (%) \| \| \| \| \| |
| --- | --- | --- | --- | --- | --- | --- | --- | --- | --- | --- | --- | --- | --- | --- | --- | --- | --- | --- | --- | --- | --- | --- | --- | --- | --- | --- | --- | --- | --- | --- | --- | --- | --- | --- | --- | --- | --- | --- | --- | --- | --- | --- | --- | --- | --- | --- | --- | --- | --- | --- | --- | --- | --- | --- | --- | --- | --- | --- | --- | --- | --- | --- | --- | --- | --- | --- | --- | --- | --- | --- | --- | --- | --- | --- | --- | --- | --- | --- | --- | --- | --- | --- | --- | --- | --- | --- | --- | --- | --- | --- | --- | --- | --- | --- | --- | --- | --- | --- | --- | --- | --- | --- | --- | --- | --- | --- | --- | --- | --- | --- | --- | --- | --- | --- | --- | --- | --- | --- | --- | --- | --- | --- | --- | --- | --- | --- | --- | --- | --- | --- | --- | --- | --- | --- | --- | --- | --- | --- | --- | --- | --- | --- | --- | --- | --- | --- | --- | --- | --- | --- | --- | --- | --- | --- | --- | --- | --- | --- | --- | --- | --- | --- | --- | --- | --- | --- | --- | --- | --- | --- | --- | --- | --- | --- | --- | --- | --- | --- | --- | --- | --- | --- | --- | --- | --- | --- | --- | --- | --- | --- | --- | --- | --- | --- | --- | --- | --- | --- | --- | --- | --- | --- | --- | --- | --- | --- | --- | --- | --- | --- | --- | --- | --- | --- | --- | --- | --- | --- | --- | --- | --- | --- | --- | --- | --- | --- | --- | --- | --- | --- | --- | --- | --- | --- | --- | --- | --- | --- | --- | --- | --- | --- | --- | --- | --- | --- | --- | --- | --- | --- | --- | --- | --- | --- | --- | --- | --- | --- | --- | --- | --- | --- | --- | --- | --- | --- | --- | --- | --- | --- | --- | --- | --- | --- | --- | --- | --- | --- | --- | --- | --- | --- | --- | --- | --- | --- | --- | --- | --- | --- | --- | --- | --- | --- | --- | --- | --- | --- | --- | --- | --- | --- | --- | --- | --- | --- | --- | --- | --- | --- | --- | --- | --- | --- | --- | --- | --- | --- | --- | --- | --- | --- | --- | --- | --- | --- | --- | --- | --- | --- | --- | --- | --- | --- | --- | --- | --- | --- | --- | --- | --- | --- | --- | --- | --- | --- | --- | --- | --- | --- | --- | --- | --- | --- | --- | --- | --- | --- | --- | --- | --- | --- | --- | --- | --- | --- | --- | --- | --- | --- | --- | --- | --- | --- | --- | --- | --- | --- | --- | --- | --- | --- | --- | --- | --- | --- | --- | --- | --- | --- | --- | --- | --- | --- | --- | --- | --- | --- | --- | --- | --- | --- | --- | --- | --- | --- | --- | --- | --- | --- | --- | --- | --- | --- | --- | --- | --- | --- | --- | --- | --- | --- | --- | --- | --- | --- | --- | --- | --- | --- | --- | --- | --- | --- | --- | --- | --- | --- | --- | --- | --- | --- | --- | --- | --- | --- | --- | --- | --- | --- | --- | --- | --- | --- | --- | --- | --- | --- | --- | --- | --- | --- | --- | --- | --- | --- | --- | --- | --- | --- | --- | --- | --- | --- | --- | --- | --- | --- | --- | --- | --- | --- | --- | --- | --- | --- | --- | --- | --- | --- | --- | --- | --- | --- | --- | --- | --- | --- | --- | --- | --- | --- | --- | --- | --- | --- | --- | --- | --- | --- | --- | --- | --- | --- | --- | --- | --- | --- | --- | --- | --- | --- | --- | --- | --- | --- | --- | --- | --- | --- | --- | --- | --- | --- | --- | --- | --- | --- | --- | --- | --- | --- | --- | --- | --- | --- | --- | --- | --- | --- | --- | --- | --- | --- | --- | --- | --- | --- | --- | --- | --- | --- | --- | --- | --- | --- | --- | --- | --- | --- | --- | --- | --- | --- | --- | --- | --- | --- | --- | --- | --- | --- | --- | --- | --- | --- | --- | --- | --- | --- | --- | --- | --- | --- | --- | --- | --- | --- | --- | --- | --- | --- | --- | --- | --- | --- | --- | --- | --- | --- | --- | --- | --- | --- | --- | --- | --- | --- | --- | --- | --- | --- | --- | --- | --- | --- | --- | --- | --- | --- | --- | --- | --- | --- | --- | --- | --- | --- | --- | --- | --- | --- | --- | --- | --- | --- | --- | --- | --- | --- | --- | --- | --- | --- | --- | --- | --- | --- | --- | --- | --- | --- | --- | --- | --- | --- | --- | --- | --- | --- | --- | --- | --- | --- | --- | --- | --- | --- | --- | --- | --- | --- | --- | --- | --- | --- | --- | --- | --- | --- | --- | --- | --- | --- | --- | --- | --- | --- | --- | --- | --- | --- | --- | --- | --- | --- | --- | --- | --- | --- |

MasterSheet4 <- MasterSheet3 |> mutate(
 Approach2 = case_when(
 str_detect(Approach,"Lat") ~ str_replace(Approach,"\\bLat\\b","Lateral"),
 str_detect(Approach,"Med") ~ str_replace(Approach,"\\bMed\\b","Medial"),
 str_detect(Approach,"Snip") ~ "Other",
 .default = Approach
),
FemurBrand2 = case_when(
 str_detect(str_to_lower(FemurBrand),"attune") ~ "Attune",
 str_detect(str_to_lower(FemurBrand),"nexgen") ~ "Nexgen",
 str_detect(str_to_lower(FemurBrand),"persona") ~ "Persona",
 str_detect(str_to_lower(FemurBrand),"genesis") ~ "Genesis",
 str_detect(str_to_lower(FemurBrand),"triathlon") ~ "Triathlon",
 str_detect(str_to_lower(FemurBrand),"rhk") ~ "RHK",
 .default = NA_character_
),
Operator2 = case_when(
 Operator == "Surgeon" ~ "Consultant",
 str_detect(Operator,"Registrar") ~ "Resident",
 .default = NA_character_
),
Anaesthetic2 = case_when(
 str_detect(Anaesthetic, "Spinal \\+ Sedation") ~ str_replace(Anaesthetic, "Spinal \\+ Sedation", "Spinal, Sedation"),
 str_detect(Anaesthetic, "Spnial") ~ str_replace(Anaesthetic, "Spnial", "Spinal"),
 str_detect(Anaesthetic, "\\bGa\\b") ~ str_replace(Anaesthetic, "\\bGa\\b", "General"),
 .default = Anaesthetic
),
Anaesthetic3 = case_when(
 str_detect(Anaesthetic2, "Spinal, General") ~ str_replace(Anaesthetic2, "Spinal, General", "General, Spinal"),
 str_detect(Anaesthetic2, "Spinal, Regional, General") ~ str_replace(Anaesthetic2, "Spinal, Regional, General", "General, Regional Block, Spinal"),
 .default = Anaesthetic2
),
TourniquetType2 = case_when(
 str_detect(TourniquetType,"Intemittent") ~ str_replace(TourniquetType,"\\bIntemittent\\b","Intermittent"),
 .default = TourniquetType
),
FemurFixation2 = case_when(
 str_detect(FemurFixation,"Cemented") ~ str_replace(FemurFixation,"\\bCemented\\b","Cement"),
 str_detect(FemurFixation,"Uncemented") ~ str_replace(FemurFixation,"\\bUncemented\\b","Cementless"),
 .default = FemurFixation
),
TibiaFixation2 = case_when(
 str_detect(TibiaFixation,"Cemented") ~ str_replace(TibiaFixation,"\\bCemented\\b","Cement"),
 str_detect(TibiaFixation,"Uncemented") ~ str_replace(TibiaFixation,"\\bUncemented\\b","Cementless"),
 .default = TibiaFixation
),
Constraint2 = case_when(
 str_detect(Constraint,"Crutiate") ~ str_replace(Constraint,"\\bCrutiate\\b","Cruciate"),
 str_detect(Constraint,"Substituting|Substituing|Sacrificing|(Cruciate Stabilised)") ~ "Posterior Stabilised",
 Constraint == "Lcck" ~ "Constrained Condylar",
 str_detect(Constraint,"Dish|Mc|Rhk|Mobile|Ts") ~ "Other",
 .default = Constraint
),
ComorbidSmoke = case_when(
 Comorbidities == "Nil" ~ "No",
 is.na(Comorbidities) ~ NA_character_,
 grepl("smoke*|active.smoke*",Comorbidities,ignore.case = TRUE) ~ "Yes",
 .default = "No"
 ),
ComorbidDiabetes = case_when(
 Comorbidities == "Nil" ~ "No",
 is.na(Comorbidities) ~ NA_character_,
 grepl("\\bt2dm\\b",Comorbidities,ignore.case = TRUE) ~ "Yes",
 .default = "No"
 ),
 ComorbidBone = case_when(
 Comorbidities == "Nil" ~ "No",
 is.na(Comorbidities) ~ NA_character_,
 grepl("osteoporosis|osteomyelitis|paget*",Comorbidities,ignore.case = TRUE) ~ "Yes",
 .default = "No"
 ),
 ComorbidAutoImmune = case_when(
 Comorbidities == "Nil" ~ "No",
 is.na(Comorbidities) ~ NA_character_,
 grepl("psoria.*arthrit*|inflamm*.*arthrit*|\\bra\\b",Comorbidities,ignore.case = TRUE) ~ "Yes",
 grepl("psoriasis*|sjogren",Comorbidities,ignore.case = TRUE) ~ "Yes",
 grepl("polymyalgia rheumatica*|sarcoidosis",Comorbidities,ignore.case = TRUE) ~ "Yes",
 .default = "No"
 ),
 ComorbidCancer = case_when(
 Comorbidities == "Nil" ~ "No",
 is.na(Comorbidities) ~ NA_character_,
 grepl("\\bca\\b|lymphom*",Comorbidities,ignore.case = TRUE) ~ "Yes",
 .default = "No"
 ),
 ComorbidCardiovascular = case_when(
 Comorbidities == "Nil" ~ "No",
 is.na(Comorbidities) ~ NA_character_,
 grepl("stroke|\\bihd\\b|\\btia\\b|\\baf\\b|cardiac|cardio*",Comorbidities,ignore.case = TRUE) ~ "Yes",
 .default = "No"
 ),
 ComorbidNeuro = case_when(
 Comorbidities == "Nil" ~ "No",
 is.na(Comorbidities) ~ NA_character_,
 grepl("palsy",Comorbidities,ignore.case = TRUE) ~ "Yes",
 .default = "No"
 ),
 OpDelay = as.numeric(difftime(SurgeryDate,DateOfInitialExam,units = "weeks"))
 ) |>
 dplyr::select(!(c("Height","Weight","Comorbidities"))
) |> dplyr::select(
 -Approach
) |> relocate(
 Approach2,
 .after = Operator
)

Table6 <- tbl_summary(
 MasterSheet4 |> dplyr::select(
 SurgerySide,
 GroupCorrected,
 BilateralStatus2,
 Surgery_ProcedureName,
 Operator2,
 Approach2,
 Anaesthetic3,
 TourniquetType2,
 Pathology,
 AlignmentReferencing,
 SoftTissueRelease,
 FemurBrand2,
 FemurFixation2,
 TibiaFixation2,
 PatellaIntervention,
 Constraint2
 ),
 label = list(
 BilateralStatus2 ~ "Bilateral Status",
 Operator2 ~ "Operator",
 Approach2 ~ "Approach",
 Anaesthetic3 ~ "Anaesthetic",
 TourniquetType2 ~ "Tourniquet Use",
 FemurBrand2 ~ "Femur Brand",
 FemurFixation2 ~ "Femur Fixation",
 TibiaFixation2 ~ "Tibia Fixation",
 Constraint2 ~ "Prosthesis Constraint",
 PatellaIntervention ~ "Patella Resurfaced"
 ),
 type = list(
 PatellaIntervention ~ "dichotomous"
 ),
 value = list(
 PatellaIntervention ~ "Resurfaced"
 ),
 by = GroupCorrected
) |> add_overall()

as_flex_table(Table6) |> flextable::set_table_properties(
 layout = "autofit",
 width = 0.15,
 align = "center",
 opts_word = list(
 split = TRUE
 )
 )

| Table 7: Summary of surgical fields by group after initial adjustment.   \| **Characteristic** \| **Overall**  N = 568^1^ \| **Non-RAS**  N = 137^1^ \| **Pre-RAS**  N = 173^1^ \| **RAS**  N = 258^1^ \| \| --- \| --- \| --- \| --- \| --- \| \| SurgerySide \|  \|  \|  \|  \| \| Left \| 278 (49%) \| 59 (43%) \| 88 (51%) \| 131 (51%) \| \| Right \| 290 (51%) \| 78 (57%) \| 85 (49%) \| 127 (49%) \| \| Bilateral Status \|  \|  \|  \|  \| \| Simultaneous \| 53 (9.3%) \| 8 (5.8%) \| 20 (12%) \| 25 (9.7%) \| \| Staged \| 149 (26%) \| 46 (34%) \| 49 (28%) \| 54 (21%) \| \| Unilateral \| 366 (64%) \| 83 (61%) \| 104 (60%) \| 179 (69%) \| \| Surgery_ProcedureName \|  \|  \|  \|  \| \| Kap1 - Primary Total Arthroplasty (Unilateral) \| 376 (68%) \| 84 (61%) \| 115 (68%) \| 177 (72%) \| \| Kap2 - Primary Total Arthroplasty (Bilateral) \| 63 (11%) \| 12 (8.8%) \| 20 (12%) \| 31 (13%) \| \| Kap2.2 - Primary Total Arthroplasty (Subsequent Bilateral) \| 113 (20%) \| 41 (30%) \| 33 (20%) \| 39 (16%) \| \| Unknown \| 16 \| 0 \| 5 \| 11 \| \| Operator \|  \|  \|  \|  \| \| Consultant \| 402 (72%) \| 79 (58%) \| 122 (73%) \| 201 (78%) \| \| Resident \| 158 (28%) \| 58 (42%) \| 44 (27%) \| 56 (22%) \| \| Unknown \| 8 \| 0 \| 7 \| 1 \| \| Approach \|  \|  \|  \|  \| \| Lateral Parapatellar \| 30 (5.3%) \| 0 (0%) \| 12 (7.1%) \| 18 (7.0%) \| \| Medial Parapatellar \| 531 (94%) \| 137 (100%) \| 155 (92%) \| 239 (93%) \| \| Medial Parapatellar, Quads Snip \| 1 (0.2%) \| 0 (0%) \| 1 (0.6%) \| 0 (0%) \| \| Other \| 1 (0.2%) \| 0 (0%) \| 1 (0.6%) \| 0 (0%) \| \| Unknown \| 5 \| 0 \| 4 \| 1 \| \| Anaesthetic \|  \|  \|  \|  \| \| Epidural \| 5 (0.9%) \| 0 (0%) \| 4 (2.4%) \| 1 (0.4%) \| \| Epidural, Sedation \| 1 (0.2%) \| 0 (0%) \| 1 (0.6%) \| 0 (0%) \| \| General \| 100 (18%) \| 28 (20%) \| 27 (16%) \| 45 (18%) \| \| General, Epidural \| 6 (1.1%) \| 4 (2.9%) \| 0 (0%) \| 2 (0.8%) \| \| General, Regional Block \| 5 (0.9%) \| 2 (1.5%) \| 3 (1.8%) \| 0 (0%) \| \| General, Regional Block, Spinal \| 3 (0.5%) \| 3 (2.2%) \| 0 (0%) \| 0 (0%) \| \| General, Spinal \| 82 (15%) \| 10 (7.3%) \| 27 (16%) \| 45 (18%) \| \| General, Spinal, Epidural \| 1 (0.2%) \| 0 (0%) \| 0 (0%) \| 1 (0.4%) \| \| General, Spinal, Sedation \| 1 (0.2%) \| 0 (0%) \| 1 (0.6%) \| 0 (0%) \| \| Regional Block, Epidural \| 1 (0.2%) \| 0 (0%) \| 1 (0.6%) \| 0 (0%) \| \| Regional Block, Spinal \| 5 (0.9%) \| 1 (0.7%) \| 4 (2.4%) \| 0 (0%) \| \| Regional Block, Spinal, Sedation \| 3 (0.5%) \| 0 (0%) \| 3 (1.8%) \| 0 (0%) \| \| Spinal \| 230 (41%) \| 57 (42%) \| 59 (35%) \| 114 (44%) \| \| Spinal, Epidural \| 7 (1.2%) \| 0 (0%) \| 2 (1.2%) \| 5 (1.9%) \| \| Spinal, Sedation \| 113 (20%) \| 32 (23%) \| 37 (22%) \| 44 (17%) \| \| Unknown \| 5 \| 0 \| 4 \| 1 \| \| Tourniquet Use \|  \|  \|  \|  \| \| Intermittent \| 45 (8.2%) \| 13 (9.5%) \| 6 (3.8%) \| 26 (10%) \| \| Not Used \| 88 (16%) \| 9 (6.6%) \| 43 (27%) \| 36 (14%) \| \| Released After Closure \| 46 (8.4%) \| 1 (0.7%) \| 26 (17%) \| 19 (7.5%) \| \| Released Before Closure \| 369 (67%) \| 114 (83%) \| 82 (52%) \| 173 (68%) \| \| Unknown \| 20 \| 0 \| 16 \| 4 \| \| Pathology \|  \|  \|  \|  \| \| Inflammatory Arthritis \| 2 (0.4%) \| 0 (0%) \| 1 (0.6%) \| 1 (0.4%) \| \| Osteoarthritis \| 555 (99%) \| 136 (99%) \| 165 (98%) \| 254 (99%) \| \| Osteonecrosis \| 1 (0.2%) \| 0 (0%) \| 1 (0.6%) \| 0 (0%) \| \| Trauma \| 4 (0.7%) \| 1 (0.7%) \| 2 (1.2%) \| 1 (0.4%) \| \| Unknown \| 6 \| 0 \| 4 \| 2 \| \| AlignmentReferencing \|  \|  \|  \|  \| \| Im Femur/Em Tibia \| 116 (21%) \| 93 (68%) \| 23 (14%) \| 0 (0%) \| \| Navigation \| 183 (33%) \| 43 (32%) \| 140 (86%) \| 0 (0%) \| \| Robotic \| 254 (46%) \| 0 (0%) \| 0 (0%) \| 254 (100%) \| \| Unknown \| 15 \| 1 \| 10 \| 4 \| \| SoftTissueRelease \|  \|  \|  \|  \| \| Combined Release/Tighten \| 1 (0.2%) \| 1 (0.7%) \| 0 (0%) \| 0 (0%) \| \| Extensive Lateral \| 10 (1.8%) \| 0 (0%) \| 4 (2.4%) \| 6 (2.4%) \| \| Extensive Medial \| 27 (4.9%) \| 1 (0.7%) \| 12 (7.3%) \| 14 (5.5%) \| \| Minimal Lateral \| 29 (5.2%) \| 0 (0%) \| 21 (13%) \| 8 (3.2%) \| \| Minimal Medial \| 403 (73%) \| 124 (91%) \| 74 (45%) \| 205 (81%) \| \| Minimal Medial, Itb Pie Crust \| 4 (0.7%) \| 4 (2.9%) \| 0 (0%) \| 0 (0%) \| \| None \| 75 (14%) \| 6 (4.4%) \| 49 (30%) \| 20 (7.9%) \| \| Other \| 5 (0.9%) \| 0 (0%) \| 5 (3.0%) \| 0 (0%) \| \| Unknown \| 14 \| 1 \| 8 \| 5 \| \| Femur Brand \|  \|  \|  \|  \| \| Attune \| 51 (9.3%) \| 51 (37%) \| 0 (0%) \| 0 (0%) \| \| Genesis \| 18 (3.3%) \| 18 (13%) \| 0 (0%) \| 0 (0%) \| \| Nexgen \| 38 (6.9%) \| 2 (1.5%) \| 36 (23%) \| 0 (0%) \| \| Persona \| 392 (71%) \| 14 (10%) \| 121 (77%) \| 257 (100%) \| \| RHK \| 2 (0.4%) \| 2 (1.5%) \| 0 (0%) \| 0 (0%) \| \| Triathlon \| 50 (9.1%) \| 50 (36%) \| 0 (0%) \| 0 (0%) \| \| Unknown \| 17 \| 0 \| 16 \| 1 \| \| Femur Fixation \|  \|  \|  \|  \| \| Cement \| 347 (63%) \| 84 (62%) \| 109 (69%) \| 154 (60%) \| \| Cementless \| 203 (37%) \| 52 (38%) \| 49 (31%) \| 102 (40%) \| \| Unknown \| 18 \| 1 \| 15 \| 2 \| \| Tibia Fixation \|  \|  \|  \|  \| \| Cement \| 459 (83%) \| 92 (67%) \| 144 (89%) \| 223 (88%) \| \| Cementless \| 92 (17%) \| 45 (33%) \| 17 (11%) \| 30 (12%) \| \| Unknown \| 17 \| 0 \| 12 \| 5 \| \| Patella Resurfaced \| 429 (76%) \| 80 (58%) \| 124 (73%) \| 225 (88%) \| \| Unknown \| 5 \| 0 \| 4 \| 1 \| \| Prosthesis Constraint \|  \|  \|  \|  \| \| Constrained Condylar \| 5 (0.9%) \| 2 (1.5%) \| 2 (1.3%) \| 1 (0.4%) \| \| Cruciate Retaining \| 466 (84%) \| 118 (86%) \| 126 (79%) \| 222 (87%) \| \| Medial Pivot \| 34 (6.2%) \| 0 (0%) \| 14 (8.8%) \| 20 (7.8%) \| \| Other \| 18 (3.3%) \| 7 (5.1%) \| 6 (3.8%) \| 5 (2.0%) \| \| Posterior Stabilised \| 29 (5.3%) \| 10 (7.3%) \| 11 (6.9%) \| 8 (3.1%) \| \| Unknown \| 16 \| 0 \| 14 \| 2 \| \| ^1^n (%) \| \| \| \| \| |
| --- | --- | --- | --- | --- | --- | --- | --- | --- | --- | --- | --- | --- | --- | --- | --- | --- | --- | --- | --- | --- | --- | --- | --- | --- | --- | --- | --- | --- | --- | --- | --- | --- | --- | --- | --- | --- | --- | --- | --- | --- | --- | --- | --- | --- | --- | --- | --- | --- | --- | --- | --- | --- | --- | --- | --- | --- | --- | --- | --- | --- | --- | --- | --- | --- | --- | --- | --- | --- | --- | --- | --- | --- | --- | --- | --- | --- | --- | --- | --- | --- | --- | --- | --- | --- | --- | --- | --- | --- | --- | --- | --- | --- | --- | --- | --- | --- | --- | --- | --- | --- | --- | --- | --- | --- | --- | --- | --- | --- | --- | --- | --- | --- | --- | --- | --- | --- | --- | --- | --- | --- | --- | --- | --- | --- | --- | --- | --- | --- | --- | --- | --- | --- | --- | --- | --- | --- | --- | --- | --- | --- | --- | --- | --- | --- | --- | --- | --- | --- | --- | --- | --- | --- | --- | --- | --- | --- | --- | --- | --- | --- | --- | --- | --- | --- | --- | --- | --- | --- | --- | --- | --- | --- | --- | --- | --- | --- | --- | --- | --- | --- | --- | --- | --- | --- | --- | --- | --- | --- | --- | --- | --- | --- | --- | --- | --- | --- | --- | --- | --- | --- | --- | --- | --- | --- | --- | --- | --- | --- | --- | --- | --- | --- | --- | --- | --- | --- | --- | --- | --- | --- | --- | --- | --- | --- | --- | --- | --- | --- | --- | --- | --- | --- | --- | --- | --- | --- | --- | --- | --- | --- | --- | --- | --- | --- | --- | --- | --- | --- | --- | --- | --- | --- | --- | --- | --- | --- | --- | --- | --- | --- | --- | --- | --- | --- | --- | --- | --- | --- | --- | --- | --- | --- | --- | --- | --- | --- | --- | --- | --- | --- | --- | --- | --- | --- | --- | --- | --- | --- | --- | --- | --- | --- | --- | --- | --- | --- | --- | --- | --- | --- | --- | --- | --- | --- | --- | --- | --- | --- | --- | --- | --- | --- | --- | --- | --- | --- | --- | --- | --- | --- | --- | --- | --- | --- | --- | --- | --- | --- | --- | --- | --- | --- | --- | --- | --- | --- | --- | --- | --- | --- | --- | --- | --- | --- | --- | --- | --- | --- | --- | --- | --- | --- | --- | --- | --- | --- | --- | --- | --- | --- | --- | --- | --- | --- | --- | --- | --- | --- | --- | --- | --- | --- | --- | --- | --- | --- | --- | --- | --- | --- | --- | --- | --- | --- | --- | --- | --- | --- | --- | --- | --- | --- | --- | --- | --- | --- | --- | --- | --- | --- | --- | --- | --- | --- | --- | --- | --- | --- | --- | --- | --- | --- | --- | --- | --- | --- | --- | --- | --- | --- | --- | --- | --- | --- | --- | --- | --- | --- | --- | --- | --- | --- | --- | --- | --- | --- | --- | --- | --- | --- | --- | --- | --- | --- | --- | --- | --- | --- | --- | --- | --- | --- | --- | --- | --- | --- | --- | --- | --- | --- | --- | --- | --- | --- | --- |

### 5.10.2 IDEAL Stage2b [3] Outcomes

*A range of outcomes should be collected with standardised definitions*

#### Procedure Duration

Procedure start and end times were captured during Chart Review (2). Durations were calculated using *lubridate*.

MasterSheet5 <- MasterSheet4 |> rename(
 OperationStart = `Operation Start Time`,
 OperationFinish = `Operation Finish Time`
 ) |> mutate(
 OperationStart = as.POSIXct(OperationStart, tz = "", format = "%Y-%m-%d %H:%M:%OS"),
 OperationFinish = as.POSIXct(OperationFinish, tz = "", format = "%Y-%m-%d %H:%M:%OS")) |>
 mutate(ProcedureDuration = as.numeric(difftime(OperationFinish,OperationStart, units = "mins")))

Cases over 4 hours were retrieved for manual review.

knitr::kable(MasterSheet5 |>
 filter(ProcedureDuration >239) |>
 dplyr::select(ProgressIndicator,
 ProgressComment,
 BilateralStatus,
 ProcedureDuration,
 PatientID,
 SurgeryDate,
 SurgerySide,
 Surgeon,
 Operator,
 GroupCorrected) |>
 arrange(GroupCorrected,Surgeon),
 format = "html")

| Table 8: Individual case data for instances of procedure duration over 4 hours.   \| ProgressIndicator \| ProgressComment \| BilateralStatus \| ProcedureDuration \| PatientID \| SurgeryDate \| SurgerySide \| Surgeon \| Operator \| GroupCorrected \| \| --- \| --- \| --- \| --- \| --- \| --- \| --- \| --- \| --- \| --- \| \| Done Jb \| NA \| Bilateral \| 254 \| 404306 \| 2021-03-10 \| Right \| G \| Surgeon \| Non-RAS \| \| Done Jb \| NA \| Bilateral \| 254 \| 404306 \| 2021-03-10 \| Left \| G \| Surgeon \| Non-RAS \| \| Done Fl \| Ng Lcck Femoral/Tibia/Poly, Persona Patella. Jb: Long Op Time Due To Difficulty Trialling Cr Components --> Intraop Decision To Convert To Lcck \| Unilateral \| 272 \| 613542 \| 2019-11-05 \| Left \| B \| Surgeon \| Pre-RAS \| \| R/V Jb \| Jb: Long Procedure - Combined Procedure W Replacement Of Femoral Nail. Ps Femoral Component. \| Unilateral \| 310 \| 788847 \| 2020-02-11 \| Right \| B \| Surgeon \| Pre-RAS \| \| Done Jb \| NA \| Bilateral \| 259 \| 703510 \| 2020-08-12 \| Left \| C \| Senior Resident/Registrar \| Pre-RAS \| \| Done Jb \| NA \| Bilateral \| 259 \| 703510 \| 2020-08-12 \| Right \| C \| Surgeon \| Pre-RAS \| \| Done Fl \| Pji W/ Dair 19/Sep/2020 Not 2021 \| Bilateral \| 266 \| 2545 \| 2020-08-19 \| Left \| C \| Senior Resident/Registrar \| Pre-RAS \| \| Done Fl \| NA \| Bilateral \| 266 \| 2545 \| 2020-08-19 \| Right \| C \| Surgeon \| Pre-RAS \| \| Done Fl \| Mc Poly \| Bilateral \| 241 \| 2011326 \| 2021-07-21 \| Left \| C \| Senior Resident/Registrar \| Pre-RAS \| \| Done Fl \| Mc Poly \| Bilateral \| 241 \| 2011326 \| 2021-07-21 \| Right \| C \| Surgeon \| Pre-RAS \| \| Done Jb \| NA \| Bilateral \| 250 \| 2013648 \| 2022-06-08 \| Left \| C \| Surgeon \| RAS \| \| Done Jb \| NA \| Bilateral \| 250 \| 2013648 \| 2022-06-08 \| Right \| C \| Senior Resident/Registrar \| RAS \| \| Done Fl \| NA \| Bilateral \| 265 \| 2011689 \| 2022-08-17 \| Left \| C \| Surgeon \| RAS \| \| Done Fl \| NA \| Bilateral \| 265 \| 2011689 \| 2022-08-17 \| Right \| C \| Senior Resident/Registrar \| RAS \| \| Done Fl \| Jb: Extensive Op Time As Diffculty Achieving Adequate Extension Gap W Rosa. Decision Made To Increase Constraint --> Cck Prosthesis \| Unilateral \| 281 \| 699814 \| 2022-09-14 \| Left \| C \| Surgeon \| RAS \| \| Done Jb \| NA \| Bilateral \| 241 \| 2045777 \| 2022-11-29 \| Left \| C \| Surgeon \| RAS \| \| Done Jb \| NA \| Bilateral \| 241 \| 2045777 \| 2022-11-29 \| Right \| C \| Senior Resident/Registrar \| RAS \| \| Done Jb (R/V!) --> \| Nb: Alignment Referencing Converted From Rosa To Instrumental During Procedure- See Op Notes \| Bilateral \| 346 \| 2045744 \| 2022-11-30 \| Left \| C \| Surgeon \| RAS \| \| Done Jb \| ^Jb: See Above For Long Op Time + R Knee Re-Opened To Inspect For Bleeding \| Bilateral \| 346 \| 2045744 \| 2022-11-30 \| Right \| C \| Senior Resident/Registrar \| RAS \| |
| --- | --- | --- | --- | --- | --- | --- | --- | --- | --- | --- | --- | --- | --- | --- | --- | --- | --- | --- | --- | --- | --- | --- | --- | --- | --- | --- | --- | --- | --- | --- | --- | --- | --- | --- | --- | --- | --- | --- | --- | --- | --- | --- | --- | --- | --- | --- | --- | --- | --- | --- | --- | --- | --- | --- | --- | --- | --- | --- | --- | --- | --- | --- | --- | --- | --- | --- | --- | --- | --- | --- | --- | --- | --- | --- | --- | --- | --- | --- | --- | --- | --- | --- | --- | --- | --- | --- | --- | --- | --- | --- | --- | --- | --- | --- | --- | --- | --- | --- | --- | --- | --- | --- | --- | --- | --- | --- | --- | --- | --- | --- | --- | --- | --- | --- | --- | --- | --- | --- | --- | --- | --- | --- | --- | --- | --- | --- | --- | --- | --- | --- | --- | --- | --- | --- | --- | --- | --- | --- | --- | --- | --- | --- | --- | --- | --- | --- | --- | --- | --- | --- | --- | --- | --- | --- | --- | --- | --- | --- | --- | --- | --- | --- | --- | --- | --- | --- | --- | --- | --- | --- | --- | --- | --- | --- | --- | --- | --- | --- | --- | --- | --- | --- | --- | --- | --- | --- | --- | --- | --- | --- | --- | --- | --- | --- | --- | --- | --- | --- | --- | --- |

After manual review, it was concluded that Increased operative time reflected bilateral cases, technical issues and intraoperative complications.

#### Adverse events

ComplicResponse2a <- ComplicResponse2 |> rename(
 PatientID = "URN"
)

MasterSheet5a <- MasterSheet5 |>
 mutate(ComplicCount = sapply(seq_along(unlist(PatientID)), function(i) {
 sum(str_count(unlist(ComplicResponse2a$PatientID), unlist(PatientID)[i]))
 }))

Manually adjust data entry errors (dates). Calculate occurrence delay (DateSurgery -> Date of Occurrence) and reoperation delay (DateSurgery -> Reoperation Date). Flag any cases where the differences are extreme (negative, extreme positive).

ComplicResponse3 <- ComplicResponse2a |> rename_with(
 ~ str_replace_all(., " ", "")
) |> filter(
 !(ComplicationOccurrence == "No")
) |> mutate(
 ComplicationNature = str_replace(ComplicationNature,"#","fracture"),
 extracted_date = str_extract(ComplicationNature, "\\d{2}-(?:[A-Za-z]{3}|\\d{2})-\\d{4}"),
 parsed_date = parse_date_time(extracted_date, "d-b-Y")
) |> mutate( #fix a couple of individual responses
 parsed_date = case_when(
 ResponseID == "5258200" ~ ymd("2019-09-27"),
 ResponseID == "5333794" ~ NA_POSIXct_,
 .default = parsed_date
 ),
 DateofOccurrence2 = if_else(
 !is.na(parsed_date),
 parsed_date,
 ymd(DateofOccurrence),
 NA_POSIXct_
 )
)

The free-text describing the nature of the complication or adverse event was pre-processed using *tidytext* (0.4.3) (Silge and Robinson 2016) to split into word tokens and remove stop words.

Stop <- tibble(get_stopwords())

ComplicResponse4 <- tidytext::unnest_tokens(
 ComplicResponse3,
 output = Term,
 input = ComplicationNature,
 token = "words",
 format = "text",
 to_lower = TRUE,
 drop = FALSE
) |> anti_join(
 Stop,
 by = c("Term" = "word")
) |> mutate(
 TermLength = stringr::str_length(Term)
)

Terms with less than four characters were extracted and reproduced in an external file for manual spelling of abbreviations. Terms with digits (e.g. L5) were removed.

# Retrieve terms less than 4 characters that likely need to be recast into full words

TargetTerms <- ComplicResponse4 |> dplyr::select(
 ResponseID,
 Term
 ) |> distinct(
 Term,
 .keep_all = TRUE
 ) |> mutate(
 TermLength = stringr::str_length(Term)
 ) |> filter(
 TermLength < 5 & stringr::str_detect(Term,"\\d",negate = TRUE)
 ) |> arrange(
 Term
 )

# Commented out after first use
googlesheets4::range_write(
ss= "[data dictionary URL removed for anonymisation]",
data = TargetTerms |> dplyr::select(
 ResponseID,
 Term
),
sheet = "ComplicTerm",
range = paste0("A1:","B",nrow(TargetTerms)+1),
col_names = TRUE

)

The abbreviated terms with expanded definitions were read back into the workspace for replacement in the complication descriptions.

# read in new terms

TargetTerms2 <- googlesheets4::range_read(
 ss= "[data dictionary URL removed for anonymisation]",
 sheet = "ComplicTerm",
 range = "A1:C",
 col_names = TRUE,
 trim_ws = TRUE
) |> mutate(
 TargetTerm = paste0("\\b",Term,"\\b")
)

The terms were replaced and added to the dataframe containing complication data.

TargetTermsList <- str_c(TargetTerms2$TargetTerm, collapse = "|")

ReplaceTermFun <- function(String) {
 # Find the matched term in TargetTerms3
 match <- filter(TargetTerms2,
 String == Term)

 # Check if a match is found
 if (nrow(match) ==1) {
 return(match$ReplaceTerm)
 } else {
 # Return the original string if no match is found
 return(String)
 }
}

# ComplicResponse5 <- ComplicResponse4 |> mutate(
# Term1 = case_when(
# TermLength < 5 & str_detect(Term,"\\d", negate = TRUE) ~ str_replace_all(Term, TargetTermsList, ReplaceTermFun),
# .default = Term
# )
# ) |> filter(
# str_detect(Term1,"\\d", negate = TRUE)
# )

ComplicResponse5 <- ComplicResponse4 |>
 rowwise() |>
 mutate(
 Term1 = case_when(
 TermLength < 5 & str_detect(Term, "\\d", negate = TRUE) ~ str_replace_all(Term, TargetTermsList, ReplaceTermFun),
 .default = Term
 )
 ) |>
 ungroup() |>
 filter(
 str_detect(Term1, "\\d", negate = TRUE)
 )

A figure displaying term frequency was generated using *ggplot2* and formatted for reporting using *knitr*.

Figure3 <- ComplicResponse5 |> filter(!Term1 == "treatment") |>
 count(Term1, sort = TRUE) |>
 filter(n > 3) |>
 mutate(Term1 = reorder(Term1, n)) |>
 ggplot(aes(n, Term1)) +
 geom_col() +
 labs(y = NULL)


knitr::knit_print(Figure3)

| 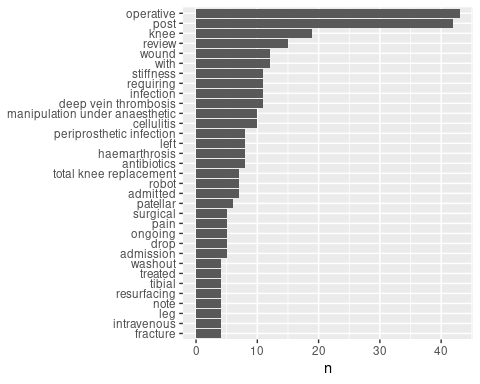  Figure 3: Complication terms by frequency. |
| --- |

A wordcloud was generated using *wordcloud* (2.6) (Fellows 2018) to express the most common terms in the complication description free text field.

Figure4 <- ComplicResponse5 |> filter(!Term1 == "treatment") |>
 count(Term1) |>
 with(wordcloud::wordcloud(Term1, n, max.words = 50))

knitr::knit_print(Figure4)

NULL

| 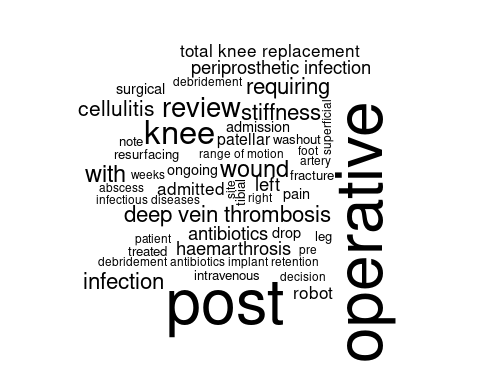  Figure 4: Word cloud of terms appearing in the adverse event descriptions. |
| --- |

#This code performs several operations:

# Initial Join:
#
# Takes ComplicResponse3 and joins it with a summarized version of ComplicResponse5
# The summarization combines all Term1 values for each ResponseID into a single space-separated string
#
#
# Date Management:
#
# Removes an old date column
# Renames DateofOccurrence2 to DateofOccurrence
# This suggests there was a date correction or update in the data
#
#
# Data Organization:
#
# Sorts the data by PatientID and DateofOccurrence
# Creates a new identifier (TempIDPart) by combining PatientID and Side
#
#
# Master Data Integration:
#
# Joins with selected columns from MasterSheet5a
# Uses a many-to-many relationship, meaning multiple rows can match on both sides
# Brings in SurgeryDate and GroupCorrected information
#
#
# Time Calculation:
#
# Calculates the delay between surgery and occurrence dates
# Converts the dates using ymd() (assumes dates are in YYYY-MM-DD format)
# Expresses the time difference in days

ComplicResponse6 <- left_join(
 ComplicResponse3,
 ComplicResponse5 |>
 group_by(
 ResponseID
 ) |>
 summarize(
 Term2 = str_c(Term1, collapse = " ")
 ) |> ungroup(),
 by = "ResponseID"
) |> dplyr::select(
 -DateofOccurrence
) |> rename(
 DateofOccurrence = "DateofOccurrence2"
) |> arrange(
 PatientID,
 DateofOccurrence
) |> mutate(
 TempIDPart = paste0(PatientID,Side)
) |> left_join(
 MasterSheet5a |> dplyr::select(
 TempIDPart,
 SurgeryDate,
 GroupCorrected
 ),
 by = "TempIDPart",
 relationship = "many-to-many"
) |> mutate(
 OccurDelay = as.numeric(as.duration(interval(ymd(SurgeryDate), ymd(DateofOccurrence))),"days")
 )

Retrieve problem entries

ComplicProblem <- ComplicResponse6 |> filter(
 !(between(OccurDelay,0,365))
)

Data entry errors were corrected manually by shifting date of occurrence entries forward or backwards by years to align with the surgery date appropriately. Regular expressions were used to match terms to consensus adverse event definitions for total knee arthroplasty (Healy et al. 2013).

ComplicResponse6a <- ComplicResponse6 |>
 mutate(
 DateofOccurrence = case_when(
 ResponseID == "5258079" ~ DateofOccurrence + years(1),
 ResponseID == "5627467" ~ DateofOccurrence + years(1),
 ResponseID == "5258108" ~ DateofOccurrence - years(3),
 .default = DateofOccurrence
 ),
 ReoperationDate = case_when(
 ResponseID == "5627467" ~ DateofOccurrence + years(1),
 .default = ReoperationDate
 )
 ) |> mutate(
 OccurDelay = as.numeric(interval(ymd(SurgeryDate), ymd(DateofOccurrence)), "days")
 ) |> mutate(
 Bleed = if_else(str_detect(Term2,"bleed*|haemarth*|transfusion") & ComplicationType == "Post-Op","Bleed","No"),
 Wound = if_else(str_detect(Term2,"\\b(delay\\w*|heal\\w*|breakdown|necrosis)\\b") & ComplicationType == "Post-Op","Wound","No"),
 Thromboembolism = if_else(str_detect(Term2,"thromb*|embol*|occul*"),"Thromboembolism","No"),
 Neural = if_else(str_detect(Term2,"palsy|nerve*|(foot drop)"),"Neural","No"),
 Vascular = if_else(str_detect(Term2,"bleed*") & ComplicationType == "In-theatre","Vascular","No"),
 #Medial collateral injury - no occurrences
 Instability = if_else(str_detect(Term2,"instab*|unstable") & ComplicationType == "Post-Op","Instability","No"),
 #Malalignment - no occurrences
 Stiffness = if_else(str_detect(Term2,"range|stiff*|motion|arthrofib*|manipulat*|loss.extens*") & ComplicationType == "Post-Op","Stiffness","No"),
 Infection1 = if_else(str_detect(Term2,"superficial|absc*|cellulitis|(surgical site)|wound.+infect*") & ComplicationType == "Post-Op","InfectionSuperficial","No"),
 Infection2 = if_else(str_detect(Term2,"(implant.+retention)|(periprosthetic.+infect\\w*)|(prosthe\\w*.+infect\\w*)") & ComplicationType == "Post-Op","InfectionDeep","No"),
 Infection3 = if_else(str_detect(Term2,"(urinary.+infect*)|diverticulitis") & ComplicationType == "Post-Op","InfectionSyst","No"),
 PeriprostheticFracture = if_else(str_detect(Term2,"fracture*"),"Fracture","No"),
 Extensor = if_else(str_detect(Term2,"extensor.+mechansism") & ComplicationType == "Post-Op","Extensor","No"),
 Patellofemoral = if_else(str_detect(Term2,"patella*|patello*") & ComplicationType == "Post-Op","Patellofemoral","No"),
 Dislocation = if_else(str_detect(Term2,"disloc*") & ComplicationType == "Post-Op","Dislocation","No"),
 Delivery = if_else(str_detect(Term2,"robot|conver*") & ComplicationType == "In-theatre","Delivery","No"),
 Loosening = if_else(str_detect(Term2,"loosening|loose") & ComplicationType == "Post-Op","Loosening","No"),
 Revision = if_else(str_detect(Term2,"revision|revise*") & ComplicationType == "Post-Op" & !is.na(ReoperationDate),"Revision","No"),
 Reoperation = if_else(!is.na(ReoperationDate) & Revision == "No","Reoperation","No"),
 Readmission = if_else((Revision == "Revision"|Reoperation == "Reoperation")|str_detect(Term2,"readmi*|admit*|admiss*|hospital") & ComplicationType != "In-theatre","Readmission","No")
 #Mortality - no occurrences
 ) |> rowwise() |> mutate(
 Other = case_when(
 if_all(Bleed:Delivery, ~ str_detect(., "No")) ~ "Other",
 .default = "No"
 )
 ) |> relocate(
 Other,
 .before = Revision
 )

ComplicResponse7 <- ComplicResponse6a |> pivot_longer(
 cols = c(
 "Bleed",
 "Wound",
 "Thromboembolism",
 "Neural",
 "Vascular",
 "Instability",
 "Stiffness",
 "Infection1",
 "Infection2",
 "Infection3",
 "PeriprostheticFracture",
 "Extensor",
 "Patellofemoral",
 "Dislocation",
 "Delivery",
 "Loosening",
 "Other",
 "Reoperation",
 "Readmission"
 ),
 cols_vary = "slowest",
 names_to = "Category_Name",
 values_to = "Category_Value",
 values_drop_na = FALSE
) |> filter(
 !(Category_Value == "No"),
 OccurDelay <= 90
) |> group_by(
 TempIDPart
) |> arrange(
 desc(DateofOccurrence),
 .by_group = TRUE
) |> distinct( #duplicate entries for the same patient(Treatment)
 pick(TempIDPart,
 DateofOccurrence,
 Category_Value
 ),
 .keep_all = TRUE
) |> ungroup()

CutOffDate <- as.character("20230630")

MasterCensor <- MasterSheet5a |> filter(
 !(TempIDPart %in% ComplicResponse7$TempIDPart)
) |> dplyr::select(
 TempIDPart,
 SurgeryDate,
 GroupCorrected
) |> bind_rows(
 ComplicResponse7 |> dplyr::select(
 TempIDPart,
 SurgeryDate,
 GroupCorrected
 )
 ) |> distinct(
 TempIDPart,
 .keep_all = TRUE
 ) |> mutate(
 ComplicationOccurrence = "No",
 DateofOccurrence = ymd(CutOffDate),
 Category_Value = "Censored"
)

#### Patient Reported Outcomes

MasterPROMs <- left_join(
 MasterSheet5a |> dplyr::select(
 TempIDFull,
 AgeAtSurgery,
 Sex,
 BMI,
 GroupCorrected
 ),
 dplyr::select(DEPARTMENTAL REGISTRYSnapshot,
 TempIDFull,
 InclusionSurgeryType,
 Consent,
 ZipCode,
 VR12MentalScore_Preop,
 VR12PhysicalScore_Preop,
 Oxford12_PainTotal_Preop,
 Oxford12_FunctionTotal_Preop,
 Oxford12_itemScore_Preop,
 VASPainScore_Preop,
 EligibleAtx3months,
 VR12MentalScore_3months,
 VR12PhysicalScore_3months,
 Oxford12_PainTotal_3months,
 Oxford12_FunctionTotal_3months,
 Oxford12_itemScore_3months,
 VASPainScore_3months,
),
by = "TempIDFull"
) |> mutate(
 Group1 = factor(GroupCorrected, levels = c("RAS", "Pre-RAS", "Non-RAS"))
)

levels <- c("Preoperative" = "Preop",
 "3months" = "3months")
MasterPROMs1 <- MasterPROMs |> rename_with(
 ~ stringr::str_replace(.,"_",""),
 starts_with("Oxford")
) |> dplyr::select(
 Group1,
 TempIDFull,
 starts_with("Oxford"),
 starts_with("VR12")
) |> pivot_longer(
 cols = !c(Group1,TempIDFull),
 names_to = c(".value","TimePoint"),
 names_sep = "_",
 values_drop_na = TRUE
) |> mutate(
 TimePoint2 = forcats::fct_recode(
 TimePoint,!!!levels
 ) |> fct_relevel("Preoperative","3months")
)

TablePROMs <- tbl_summary(
 MasterPROMs1 |> dplyr::select(
 Group1,
 TimePoint2,
 starts_with("Oxford"),
 starts_with("VR12")
 ),
 by = TimePoint2,
 missing = "no",
 label = "Group1" ~ "Group"
)

as_flex_table(TablePROMs) |> flextable::set_table_properties(
 layout = "autofit",
 width = 0.15,
 align = "center",
 opts_word = list(
 split = TRUE
 )
 )

| Table 9: Summary of available patient reported outcomes from analysis dataset.   \| **Characteristic** \| **Preoperative**  N = 185^1^ \| **3months**  N = 118^1^ \| \| --- \| --- \| --- \| \| Group \|  \|  \| \| RAS \| 65 (35%) \| 26 (22%) \| \| Pre-RAS \| 91 (49%) \| 76 (64%) \| \| Non-RAS \| 29 (16%) \| 16 (14%) \| \| Oxford12PainTotal \| 30 (20, 40) \| 65 (45, 80) \| \| Oxford12FunctionTotal \| 36 (25, 46) \| 75 (55, 82) \| \| Oxford12itemScore \| 15 (12, 22) \| 33 (25, 39) \| \| VR12MentalScore \| 46 (39, 56) \| 53 (42, 60) \| \| VR12PhysicalScore \| 27 (23, 34) \| 37 (29, 45) \| \| ^1^n (%); Median (Q1, Q3) \| \| \| |
| --- | --- | --- | --- | --- | --- | --- | --- | --- | --- | --- | --- | --- | --- | --- | --- | --- | --- | --- | --- | --- | --- | --- | --- | --- | --- | --- | --- | --- | --- | --- | --- | --- | --- |

## 5.11 STROBE [9] Bias:

*Critically analyze potential sources of bias and the adequacy of methods used to address them.*

Observational cohort analyses are subject to a number of biases (Brown et al. 2023). Specific to this analysis, the following considerations are noted below.

| Table 10: Biases in a comparative observational cohort analysis.   \| **Bias** \| **Definition** \| **Source** \| **Mitigation** \| \| --- \| --- \| --- \| --- \| \| Misclassification \| The clinical notes are misinterpreted to place the case in the incorrect Group \|  \| Clinical notes reviewed by multiple reviewers.  Additional analysis checks to review grouping selected versus grouping by calculation against retrieved chart notes. \| \| Confounder \| A variable of interest and a target outcome simultaneously influenced by a third variable \| (Tennant et al. 2020) \| Regression models used to adjust models for estimated confounders identified through minimal adjustment sets using directed acyclic graphs. \| \| Missing data \| The absence of a data value where a treatment record is eligible to have a data value collected \| (Carroll et al. 2020) \| Missingness assessed to be of low impact (low incidence) to analysis dataset. \| \| Pseudoreplication \| Analyse data while ignoring dependency between observations. Inadequate model specification. \| (Davies and Gray 2015; Lazic 2010) \| Used mixed effects linear model for procedure duration with patient identifier as random effect. Cluster for patient in survival model for time-to-event outcomes (adverse events). \| |
| --- | --- | --- | --- | --- | --- | --- | --- | --- | --- | --- | --- | --- | --- | --- | --- | --- | --- | --- | --- | --- |

## 5.12 Missing Data

Missingness is an unavoidable characteristic of clinical datasets, in particular patients from non-english speaking or lower socioeconomic backgrounds are less likely to respond to patient-reported outcome measures in lower limb arthroplasty (Konopka et al. 2023). The mastersheet was adjusted using tidyverse to include only the required columns and to convert categorical columns to factors. The naniar package was used to generate a summary figure of missingness in the key variables of the analysis. Demographic covariates required for the primary models (age, sex, BMI, diabetes comorbidity) were backfilled where missing in the study dataset using an independent snapshot of the same patients held in the hospital’s clinical quality registry, matched by patient identifier and surgical side. This reduced missingness in these variables to under 1% (Table 12). Surgical and procedural fields were not backfilled in this way; missingness in these fields remained below 5% for most variables. Given the low overall missingness following the registry backfill, and the explainable pattern in the remaining fields, no further imputation was performed; models were fitted using complete-case analysis for the non-backfilled variables.

MasterSheetMiss <- MasterSheet5a |> filter(
 if_any(c(AgeAtSurgery,Sex,ComorbidDiabetes,BMI,Surgery_ProcedureName:OperationFinish), ~is.na(.))
)


DEPARTMENTAL REGISTRYSoc2a <- DEPARTMENTAL REGISTRYSoc2 |> rename(
 PatientID = "PatientId"
 ) |> mutate(
 TempIDPart = paste0(PatientID,SurgerySide1)
) |> relocate(
 TempIDPart
)

DEPARTMENTAL REGISTRYBackfill <- DEPARTMENTAL REGISTRYSoc2a |> filter(
 TempIDPart %in% MasterSheetMiss$TempIDPart
)

MasterSheet5b <- MasterSheet5a |> filter(
 TempIDPart %in% DEPARTMENTAL REGISTRYBackfill$TempIDPart
) |> filter(
 !(TempIDFull == "412600Right44896")
)

MismatchSHARK <- anti_join(
 MasterSheet5b,
 DEPARTMENTAL REGISTRYBackfill,
 by = "TempIDPart"
)

MissingMaster <- naniar::as_shadow(MasterSheet5b) |> mutate(Sheet = "StudyMaster")

MissingDEPARTMENTAL REGISTRY <- naniar::as_shadow(
 DEPARTMENTAL REGISTRYBackfill
 ) |> mutate(
 Sheet = "RegistrySnapshot"
 )

MissingComp <-bind_rows(
 MissingMaster,
 MissingDEPARTMENTAL REGISTRY
) |> relocate(
 Sheet,
 .before = ProgressIndicator_NA
)

TableMiss1 <- tbl_summary(
 MissingComp |> dplyr::select(
 Sheet,
 Surgery_ProcedureName_NA:OperationFinish_NA
 ),
 by = Sheet,
type = all_categorical() ~ "dichotomous",
value = everything() ~ "NA"
)

as_flex_table(TableMiss1) |> flextable::set_table_properties(
 layout = "autofit",
 width = 0.15,
 align = "center",
 opts_word = list(
 split = TRUE
 )
 )

| Table 11: Summary of missing values within surgical fields of analysis dataset.   \| **Characteristic** \| **RegistrySnapshot**  N = 199^1^ \| **StudyMaster**  N = 196^1^ \| \| --- \| --- \| --- \| \| Surgery_ProcedureName_NA \| 0 (NA%) \| 16 (8.2%) \| \| Unknown \| 199 \| 0 \| \| Operator_NA \| 14 (7.0%) \| 5 (2.6%) \| \| Approach2_NA \| 0 (NA%) \| 5 (2.6%) \| \| Unknown \| 199 \| 0 \| \| Anaesthetic_NA \| 14 (7.0%) \| 5 (2.6%) \| \| TourniquetType_NA \| 28 (14%) \| 20 (10%) \| \| Pathology_NA \| 16 (8.0%) \| 6 (3.1%) \| \| AlignmentReferencing_NA \| 26 (13%) \| 15 (7.7%) \| \| SoftTissueRelease_NA \| 23 (12%) \| 13 (6.6%) \| \| FemurBrand_NA \| 29 (15%) \| 17 (8.7%) \| \| FemurFixation_NA \| 30 (15%) \| 17 (8.7%) \| \| FemoralSize_NA \| 29 (15%) \| 17 (8.7%) \| \| TibiaBrand_NA \| 27 (14%) \| 15 (7.7%) \| \| TibiaFixation_NA \| 29 (15%) \| 17 (8.7%) \| \| TibialSize_NA \| 28 (14%) \| 16 (8.2%) \| \| InsertThickness_NA \| 32 (16%) \| 21 (11%) \| \| PatellaIntervention_NA \| 14 (7.0%) \| 5 (2.6%) \| \| PatellaType_NA \| 92 (46%) \| 92 (47%) \| \| PatellaSize_NA \| 105 (53%) \| 160 (82%) \| \| Constraint_NA \| 28 (14%) \| 16 (8.2%) \| \| OperationStart_NA \| 0 (NA%) \| 2 (1.0%) \| \| Unknown \| 199 \| 0 \| \| OperationFinish_NA \| 0 (NA%) \| 2 (1.0%) \| \| Unknown \| 199 \| 0 \| \| ^1^n (%) \| \| \| |
| --- | --- | --- | --- | --- | --- | --- | --- | --- | --- | --- | --- | --- | --- | --- | --- | --- | --- | --- | --- | --- | --- | --- | --- | --- | --- | --- | --- | --- | --- | --- | --- | --- | --- | --- | --- | --- | --- | --- | --- | --- | --- | --- | --- | --- | --- | --- | --- | --- | --- | --- | --- | --- | --- | --- | --- | --- | --- | --- | --- | --- | --- | --- | --- | --- | --- | --- | --- | --- | --- | --- | --- | --- | --- | --- | --- | --- | --- | --- | --- | --- | --- |

MissingMaster2 <- naniar::as_shadow(MasterSheet5a)

TableMiss2 <- tbl_summary(
 MissingMaster2 |> dplyr::select(
 AgeAtSurgery_NA,
 Sex_NA,
 BMI_NA,
 ComorbidDiabetes_NA,
 Surgery_ProcedureName_NA:OperationFinish_NA),
type = all_categorical() ~ "dichotomous",
value = everything() ~ "NA"
)

as_flex_table(TableMiss2) |> flextable::set_table_properties(
 layout = "autofit",
 width = 0.15,
 align = "center",
 opts_word = list(
 split = TRUE
 )
 )

| Table 12: Overall missingness rates for key variables within the analysis dataset.   \| **Characteristic** \| **N = 568**^1^ \| \| --- \| --- \| \| AgeAtSurgery_NA \| 3 (0.5%) \| \| Sex_NA \| 0 (0%) \| \| BMI_NA \| 0 (0%) \| \| ComorbidDiabetes_NA \| 0 (0%) \| \| Surgery_ProcedureName_NA \| 16 (2.8%) \| \| Operator_NA \| 5 (0.9%) \| \| Approach2_NA \| 5 (0.9%) \| \| Anaesthetic_NA \| 5 (0.9%) \| \| TourniquetType_NA \| 20 (3.5%) \| \| Pathology_NA \| 6 (1.1%) \| \| AlignmentReferencing_NA \| 15 (2.6%) \| \| SoftTissueRelease_NA \| 14 (2.5%) \| \| FemurBrand_NA \| 17 (3.0%) \| \| FemurFixation_NA \| 18 (3.2%) \| \| FemoralSize_NA \| 17 (3.0%) \| \| TibiaBrand_NA \| 15 (2.6%) \| \| TibiaFixation_NA \| 17 (3.0%) \| \| TibialSize_NA \| 16 (2.8%) \| \| InsertThickness_NA \| 21 (3.7%) \| \| PatellaIntervention_NA \| 5 (0.9%) \| \| PatellaType_NA \| 161 (28%) \| \| PatellaSize_NA \| 218 (38%) \| \| Constraint_NA \| 16 (2.8%) \| \| OperationStart_NA \| 2 (0.4%) \| \| OperationFinish_NA \| 2 (0.4%) \| \| ^1^n (%) \| \| |
| --- | --- | --- | --- | --- | --- | --- | --- | --- | --- | --- | --- | --- | --- | --- | --- | --- | --- | --- | --- | --- | --- | --- | --- | --- | --- | --- | --- | --- | --- | --- | --- | --- | --- | --- | --- | --- | --- | --- | --- | --- | --- | --- | --- | --- | --- | --- | --- | --- | --- | --- | --- | --- | --- | --- |

FigureMiss3 <- gg_miss_var(MasterSheet5a |> dplyr::select(
 AgeAtSurgery,
 Sex,
 BMI,
 ComorbidDiabetes,
 ComorbidSmoke,
),
show_pct = TRUE
)

knitr::knit_print(FigureMiss3)

| 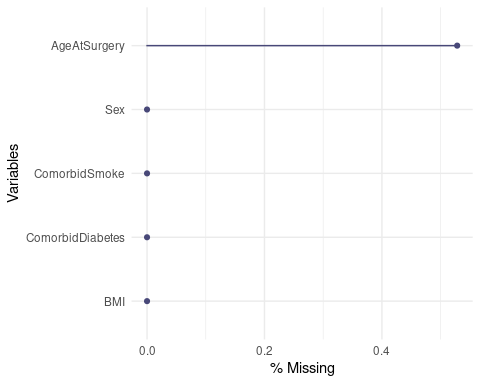  Figure 5: Summary of missingness of key demographic variables for analysis sample. |
| --- |

## 5.13 STROBE [11] Quantitative variables:

*Evaluate the analytical choices of the quantitative variables within the dataset.*

Quantitative variables in this analysis fall into three categories: continuous covariates (age at surgery, body mass index, procedure duration), binary adverse event indicators (presence/absence of each complication category, used for univariate comparisons), and time-to-event variables (used for survival models). For the time-to-event variables, each treated joint could record more than one adverse event over the follow-up period, so occurrence dates were ordered chronologically per joint and converted into a sequence of start–stop intervals, allowing multiple complications for the same joint to be represented without losing the order in which they occurred. Where a specific adverse event of interest (superficial infection, stiffness, readmission, thromboembolism) did not occur for a given joint, time was right-censored at 90 days post-surgery, consistent with the analysis follow-up window; the first occurrence of each event type was then carried forward as the input to the corresponding survival model.

ComplicMaster <- bind_rows(
 ComplicResponse7,
 MasterCensor
) |> mutate(
 Category = fct(
 Category_Value,
 levels = c(
 "Censored",
 "Fracture",
 "Thromboembolism",
 "Bleed",
 "InfectionSuperficial",
 "Delivery",
 "Stiffness",
 "Neural",
 "Vascular",
 "Patellofemoral",
 "InfectionDeep",
 "Other",
 "Wound",
 "InfectionSyst",
 "Reoperation",
 "Readmission"
 )
 )
) |> mutate(
 OccurDelay = if_else(is.na(OccurDelay),90,OccurDelay)
) |> group_by( # grouping here
 TempIDPart
 ) |> arrange(
 DateofOccurrence,
 .by_group = TRUE
 ) |> arrange(
 OccurDelay,
 .by_group = TRUE
 ) |> mutate(
 RowNum = row_number()
 ) |> mutate(
 DurationStart = case_when(
 RowNum > 1 ~ dplyr::lag(OccurDelay),
 .default = 0
 )
 ) |> mutate(
 DurationDiff = as.numeric(OccurDelay - DurationStart)
 ) |> rename(
 DurationStop = "OccurDelay"
 ) |> relocate(
 DurationStop, .after = DurationDiff
 ) |> mutate(
 DurationStop1 = case_when(
 DurationDiff == 0 ~ DurationStop + 1,
 .default = DurationStop
 )
 ) |> mutate(
 DurationStart1 = case_when(
 RowNum > 1 ~ dplyr::lag(DurationStop1),
 .default = 0
 ),
 DurationDiffLag = as.numeric(lag(DurationStop1) - DurationStart1),
 DurationDiff2 = as.numeric(DurationStop1 - DurationStart1)
 ) |> relocate(
 DurationStop1, .after = DurationStart1
 ) |> dplyr::select(
 TempIDPart,
 DurationStart1,
 DurationStop1,
 DurationDiff2,
 DurationDiffLag,
 Category,
 RowNum,
 GroupCorrected
 ) |> ungroup() |> mutate(
 Group1 = factor(GroupCorrected, levels = c("RAS", "Pre-RAS", "Non-RAS"))
 )

ComplicMaster2 <- left_join(
 ComplicMaster,
 MasterSheet5a |> filter(
 !(MockUpID == "157031left44551" & Surgeon == "B"),
 !(MockUpID == "412600right44923")
 ) |> dplyr::select(
 TempIDPart,
 Sex,
 AgeAtSurgery,
 BMI,
 BilateralStatus2,
 ComorbidSmoke,
 ComorbidDiabetes
 ),
 by = "TempIDPart"
)

category_dataframes <- ComplicMaster |>
 group_split(Category) |>
 set_names(levels(ComplicMaster$Category))

ComplicRevision <- ComplicResponse6a |> filter(Revision == "Revision")
ComplicReoperation <- ComplicResponse6a |> filter(Reoperation == "Reoperation")
ComplicReadmission <- ComplicResponse6a |> filter(Readmission == "Readmission")

MasterSheet6 <- MasterSheet5a |>
 mutate(
 Bleed = if_else(TempIDPart %in% category_dataframes$Bleed$TempIDPart, "Yes", "No"),
 Thromboembolism = if_else(TempIDPart %in% category_dataframes$Thromboembolism$TempIDPart,"Yes","No"),
 Neural = if_else(TempIDPart %in% category_dataframes$Neural$TempIDPart,"Yes","No"),
 Vascular = if_else(TempIDPart %in% category_dataframes$Vascular$TempIDPart,"Yes","No"),
 Stiffness = if_else(TempIDPart %in% category_dataframes$Stiffness$TempIDPart,"Yes","No"),
 InfectionSuperficial = if_else(TempIDPart %in% category_dataframes$InfectionSuperficial$TempIDPart,"Yes","No"),
 InfectionDeep = if_else(TempIDPart %in% category_dataframes$InfectionDeep$TempIDPart,"Yes","No"),
 InfectionSyst = if_else(TempIDPart %in% category_dataframes$InfectionSyst$TempIDPart,"Yes","No"),
 PeriprostheticFracture = if_else(TempIDPart %in% category_dataframes$Fracture$TempIDPart,"Yes","No"),
 Patellofemoral = if_else(TempIDPart %in% category_dataframes$Patellofemoral$TempIDPart,"Yes","No"),
 Delivery = if_else(TempIDPart %in% category_dataframes$Delivery$TempIDPart,"Yes","No"),
 Readmission = if_else(TempIDPart %in% ComplicReadmission$TempIDPart,"Yes","No"),
 Reoperation = if_else(TempIDPart %in% ComplicReoperation$TempIDPart,"Yes","No"),
 Revision = if_else(TempIDPart %in% ComplicRevision$TempIDPart,"Yes","No"),
 Group1 = factor(GroupCorrected, levels = c("RAS", "Pre-RAS", "Non-RAS"))
 )

## 5.14 STROBE [12] Statistical Methods:

*Evaluate the appropriateness and robustness of the statistical techniques used for analysis.*

### 5.14.1 Descriptives

Descriptives for exposure and outcome variables were summarised into tables. Univariate comparisons were between Groups for adverse events and comparisons made by Fisher’s exact test (binary outcomes) and Pearson’s chi-square tests (multilevel categorical variables) as per the *gtsummary* package functions.

### 5.14.2 Regression preparation

Set up input table for statistical analysis with only variables required for models.

MasterSheetKM <- MasterSheet6 |> dplyr::select(
 TempIDPart,
 PatientID2,
 Bleed:Group1,
 AgeAtSurgery,
 Sex,
 BMI,
 BilateralStatus2,
 ComorbidBone,
 ComorbidSmoke,
 ComorbidDiabetes,
 ComorbidCardiovascular,
 ComorbidAutoImmune
) |> dplyr::mutate(
 across(
 .cols = Bleed:Revision,
 .fns = ~case_when(
 !is.na(.) ~ if_else(.== "No",0,1),
 .default = NA
 ),
 .names = "{.col}Bin")
) |> left_join(
 ComplicMaster2 |> group_by(
 TempIDPart
 ) |> filter(
 Category == "InfectionSuperficial"
 ) |> dplyr::select(
 TempIDPart,
 DurationStop1
 ) |> slice_min(
 DurationStop1,
 n = 1),
 by = "TempIDPart"
 ) |> mutate(
 DurationInfectSuper = if_else(is.na(DurationStop1),90,DurationStop1)
 ) |> dplyr::select(
 -DurationStop1
 ) |> left_join(
 ComplicMaster2 |> group_by(
 TempIDPart
 ) |> filter(
 Category == "Stiffness"
 ) |> dplyr::select(
 TempIDPart,
 DurationStop1
 ) |> slice_min(
 DurationStop1,
 n = 1),
 by = "TempIDPart"
 ) |> mutate(
 DurationStiff = if_else(is.na(DurationStop1),90,DurationStop1)
 ) |> dplyr::select(
 -DurationStop1
 ) |> left_join(
 ComplicMaster2 |> group_by(
 TempIDPart
 ) |> filter(
 Category == "Readmission"
 ) |> dplyr::select(
 TempIDPart,
 DurationStop1
 ) |> slice_min(
 DurationStop1,
 n = 1),
 by = "TempIDPart"
 ) |> mutate(
 DurationReadmit = if_else(is.na(DurationStop1),90,DurationStop1)
 ) |> dplyr::select(
 -DurationStop1
 ) |> left_join(
 ComplicMaster2 |> group_by(
 TempIDPart
 ) |> filter(
 Category == "Thromboembolism"
 ) |> dplyr::select(
 TempIDPart,
 DurationStop1
 ) |> slice_min(
 DurationStop1,
 n = 1),
 by = "TempIDPart"
 ) |> mutate(
 DurationThrombo = if_else(is.na(DurationStop1),90,DurationStop1)
 ) |> dplyr::select(
 -DurationStop1
 ) |> ungroup()

The *ggdag* package (v0.2.13 (Barrett 2024) was used to create and visualise directed acyclic graphs (in conjunction with *ggplot2*), with minimal adjustment sets to ensure correct specification of models to estimate the effect of Group on outcome variables.

### 5.14.3 Procedure Duration

A brief literature review was conducted to identify potential factors that should be adjusted for when assessing the effect of *Group* on *Procedure Duration* in the present sample. One study from the USA (Acuña et al. 2020) examined the NSQIP database between 01-Jan-2008 and 31-Dec-2017 in 140,890 total knee arthroplasty cases (mixture of primary and revision) and reported significant correlations with a number of variables (Table 1). A registry study from Norway (Badawy et al. 2017) of 28 043 primary TKA from 2005 to 2015 identified significant correlations a number of patient and surgical variables (Table 1).

| Table 13: Summary of potential adjustment set for Procedure Duration retrieved from the literature.   \| Category \| Variable \| Source \| Available in present dataset \| \| --- \| --- \| --- \| --- \| \| Patient Factor \| Sex (Male) \| Acuna et al  Badawy et al \| ✅ \| \|  \| Age at surgery \| Acuna et al  Badawy et al \| ✅ \| \|  \| Body mass index \| Acuna et al \| ✅ \| \|  \| ASA - Class 3 \| Acuna et al  Badawy et al \| ❌ \| \|  \| Functional status \| Acuna et al \| ❌ \| \|  \| Preoperative haematocrit, sodium and INR \| Acuna et al \| ❌ \| \|  \| Smoking history \| Acuna et al \| ✅ \| \|  \| Hypertension (requiring medication) \| Acuna et al \| ✅* \| \|  \| Weight loss within previous 6 months \| Acuna et al \| ❌ \| \|  \| Diagnosis other than OA \| Badawy et al \| ✅† \| \|  \| Previous knee surgery \| Badawy et al \| ❌ \| \| Managament Factors \| Perioperative complications \| Badawy et al \| ✅ \| \|  \| Use of navigation \| Badawy et al \| ✅ \| \|  \| Implant Brand \| Badawy et al \| ✅ \| |
| --- | --- | --- | --- | --- | --- | --- | --- | --- | --- | --- | --- | --- | --- | --- | --- | --- | --- | --- | --- | --- | --- | --- | --- | --- | --- | --- | --- | --- | --- | --- | --- | --- | --- | --- | --- | --- | --- | --- | --- | --- | --- | --- | --- | --- | --- | --- | --- | --- | --- | --- | --- | --- | --- | --- | --- | --- | --- | --- | --- | --- |

*Captured as presence of cardiovascular comorbidity (CVA, MI, hypertension); † insufficient variation in this variable for inclusion into model.

Other factors included within the present context included the following;

- Bilateral surgery - considering the case mix of simultaneous bilateral surgeries
- Operator - considering the teaching hospital context, it is not possible to rule out the effect of having a junior surgeon performing the procedure on longer operative duration.
- Implant brand was discarded from the present analysis due to the high variation in implant selection and the low numbers within certain implant categories.

DAGDuration <- ggdag::dagify(ProcedureDuration ~ GuidanceGroup + Sex + Age + BMI + ComorbidSmoke + ComorbidCardiovascular + Bilateral,
 GuidanceGroup ~ Bilateral,
 ComorbidSmoke ~ Age + Sex,
 ComorbidCardiovascular ~ Age + Sex + ComorbidSmoke + BMI,
 Bilateral ~ Age,
 BMI ~ Age,
 labels = c(
 "ProcedureDuration" = "Duration of\n Procedure",
 "ComorbidSmoke" = "Smoking History",
 "ComorbidCardiovascular" = "CVD History",
 "Age" = "Age at\n Surgery",
 "GuidanceGroup" = "GuidanceGroup",
 "BMI" = "Body Mass\n Index",
 "Sex" = "Sex",
 "Bilateral" = "Bilateral Surgery"
 ),
 exposure = "GuidanceGroup",
 outcome = "ProcedureDuration"
)

# Create a tidy version of the DAG and apply custom labels
tidy_dag1 <- tidy_dagitty(DAGDuration, layout = "auto") |>
 mutate(label = case_when(
 name == "ProcedureDuration" ~ "Duration of\n Procedure",
 name == "ComorbidSmoke" ~ "Smoking History",
 name == "ComorbidCardiovascular" ~ "CVD History",
 name == "Age" ~ "Age at\n Surgery",
 name == "GuidanceGroup" ~ "GuidanceGroup",
 name == "BMI" ~ "Body Mass\n Index",
 name == "Bilateral" ~ "Bilateral Surgery",
 name == "Sex" ~ "Sex",
 TRUE ~ name
 ))

Figure5 <- ggplot(
 tidy_dag1,
 aes(
 x = x,
 y = y,
 xend = xend,
 yend = yend)
 ) +
 geom_dag_node(
 color = "darkblue",
 fill = "lightblue",
 alpha = 0.5,
 size = 20) +
 geom_dag_text(
 aes(
 label = label
 ),
 color = "black",
 fontface = "bold",
 size = 3,
 vjust = 0.5
 ) +
 geom_dag_edges(
 edge_color = "gray50",
 edge_width = 0.5,
 #edge_arrow_size = 0.3
 ) +
 theme_dag(
 base_size = 12,
 base_family = "",
 plot.background = element_rect(fill = "white"),
 panel.background = element_rect(fill = "white")
 ) +
 theme(
 plot.title = element_text(
 hjust = 0.5,
 size = 16,
 face = "bold"
 ),
 plot.margin = margin(
 20,
 20,
 20,
 20
 )
 ) +
 ggtitle(
 "Directed Acyclic Graph"
 )

knitr::knit_print(Figure5)

| 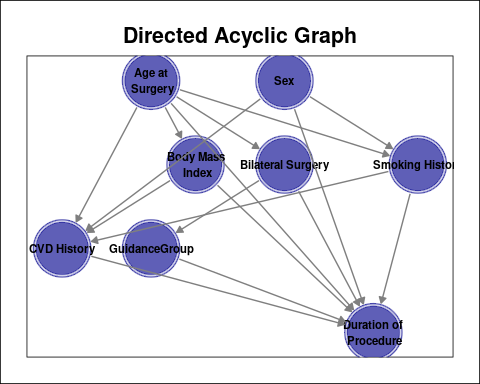  Figure 6: Directed acyclic graph of procedure duration in total knee arthroplasty. |
| --- |

Figure6 <- ggdag_adjustment_set(
 tidy_dag1,
 text = FALSE,
 #use_labels = "label"
 exposure = "GuidanceGroup",
 outcome = "ProcedureDuration"
 ) +
 geom_dag_node(
 color = "darkblue",
 fill = "lightblue",
 alpha = 0.7,
 size = 10
 ) +
 # geom_dag_text(
 # aes(label = label), # Use the 'label' column directly
 # color = "black",
 # fontface = "bold",
 # size = 3,
 # vjust = -1.5, # Adjust vertical position
 # hjust = 1.5, # Adjust horizontal position
 # show.legend = FALSE # Prevent text from appearing in legend
 # ) +
 geom_dag_label_repel(
 aes(label = label, fill = adjusted),
 color = "white",
 fontface = "bold",
 size = 3,
 show.legend = FALSE,
 box.padding = unit(0.5, "lines"), # Padding around labels
 point.padding = unit(0.5, "lines"), # Padding around points
 nudge_y = 0.5, # Adjust vertical nudge
 nudge_x = 0.5 # Adjust horizontal nudge
 ) +
 geom_dag_edges(
 edge_color = "gray50",
 edge_width = 0.5
 ) +
 theme_dag(
 base_size = 10,
 base_family = "",
 plot.background = element_rect(fill = "white"),
 panel.background = element_rect(fill = "white")
 ) +
 theme(
 plot.title = element_text(
 hjust = 0.5,
 size = 10,
 face = "bold"
 ),
 plot.margin = margin(20,20,20,20),
 legend.position = "bottom",
 legend.background = element_rect(fill = "white", color = "gray80"),
 legend.title = element_text(face = "bold")
 ) +
 ggtitle("Adjustment Set for Directed Acyclic Graph")


knitr::knit_print(Figure6)

| 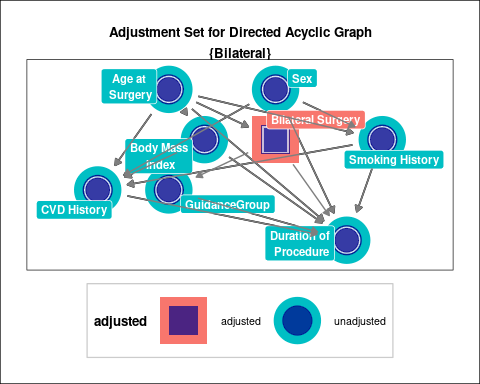  Figure 7: Calculated minimum adjustment set for modelling procedure duration in total knee arthroplasty. |
| --- |

The procedure duration outcome is characterised by positive values, overdispersion (high variance) and right skewness (some cases take substantially longer than the group median) (Figure 8 and Figure 21), as well as overdispersion (variance exceeding the average, particularly in the pre-RAS group). Although ordinary least squares is a popular regression technique for continuous data, these features of the dataset render this approach inappropriate. The *gamma* distribution is a continuous probability distribution that is suitable for modelling positive values that are skewed and has been previously demonstrated to be a robust choice in skewed medical data (Malehi et al. 2015). The master table was subset for procedure duration analysis. The dataset is also at risk of pseudoreplication due to the presence of multiple data entries for the same patient with bilateral procedures. To account for this non-independence, a patient-level random interecept was included in the model specific (*1|PatientID*), allowing each patient’s repeated observations to share a common baseline, while fixed efffects estimated the population-average effect of Group on procedure duration. A mixed-effects gamma regression model (“log” link function) with the minimal adjustment set as identified in the DAG, was applied using *lme4* package (v2.0.1). The log link was selected over alternative functions within the gamma family (inverse, identify, square root), based on model fit. All four link functions were compared using Akaike information criteria and Bayesian information criteria. The rationale and analysis for the selection are presented in STROBE [17] Other Analyses. Model predictions and post-comparisons between levels of fixed effects were calculated using the *marginaleffects* package package (v0.32.0).

Figure7 <- ggplot(data = MasterSheet6, mapping = aes(y = Group1, x = ProcedureDuration)) +
 stat_slab(aes(thickness = after_stat(pdf*n)), scale = 0.7) +
 stat_dotsinterval(side = "bottom", scale = 0.7, slab_linewidth = NA) +
 #scale_fill_brewer(palette = "Set2") +
 ggtitle('Procedure Duration by Group')

knitr::knit_print(Figure7)

| 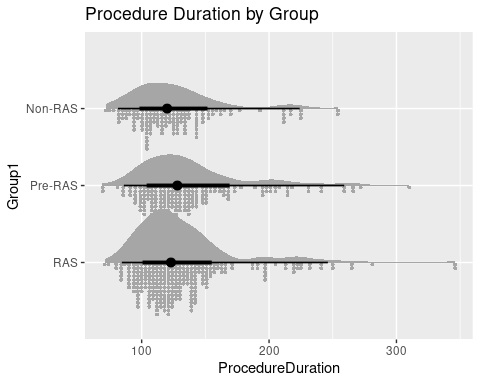  Figure 8: Initial distribution of procedure duration (observed) separated by group. |
| --- |

Duration_Analysis <- MasterSheet6 |> dplyr::select(PatientID2,
 ProcedureDuration,
 Group1,
 AgeAtSurgery,
 Sex,
 BMI,
 BilateralStatus) |>
 mutate(
 BilateralStatus = forcats::fct(BilateralStatus,
 levels = )
 #ComorbidSmoke = as.factor(ComorbidSmoke),
 #ComorbidCardiovascular = as.factor(ComorbidCardiovascular),
 #Pathology2 = as.factor(ifelse(Pathology == "Osteoarthritis", "Osteoarthritis","Other"))
 )

# Fit a mixed-effects Gamma model
Duration_model <- lme4::glmer(ProcedureDuration ~ Group1 + BilateralStatus + (1|PatientID2),
 family = Gamma(link = "log"),
 data = Duration_Analysis)

### 5.14.4 Adverse events

A brief literature review was conducted to identify potential factors that should be adjusted for when assessing the effect of *Group* on adverse events (*Superficial Infection*, *Stiffness, Thromboembolism, Readmission*) in the present sample.

For surgical site infections, one study by (Poultsides et al. 2013) reported a number of patient factors associated with SSI in primary knee arthroplasty using the USA National Inpatient Sample (N = 784 335 TKAs). A retrospective cohort (Simon and Hollenbeck 2022) identified smoking as a risk factor for SSI, and a systematic review of six articles (N = 7081 TKA) (Heifner et al. 2023) contended that local adiposity may be a more reliable predictor of infection risk after TKA, with mixed evidence over the relationship between BMI and infection risk.

| Table 14: Summary of potential adjustment set for Infection retrieved from the literature.   \| Variable \| Source \| Available in present dataset \| \| --- \| --- \| --- \| \| Sex (Male) \| Poultsides et al \| ✅ \| \| Race \| Poultsides et al \| ❌ \| \| Cancer diagnosis \| Poultsides et al \| ✅ \| \| Liver disease \| Poultsides et al \| ❌ \| \| Coagulopathies \| Poultsides et al \| ❌ \| \| Fluid \| Eloctrolyte disorders \| Poultsides et al \| ❌ \| \| Heart failure \| Poultsides et al \| ✅ * \| \| Pulmonary circulation disease \| Poultsides et al \| ✅ * \| \| Smoking history \| Simon et al \| ✅ \| \| Body mass index \| Heifner et al \| ✅ \| |
| --- | --- | --- | --- | --- | --- | --- | --- | --- | --- | --- | --- | --- | --- | --- | --- | --- | --- | --- | --- | --- | --- | --- | --- | --- | --- | --- | --- | --- | --- | --- | --- | --- | --- |

*Stored in the dataset as comorbidities of a cardiovascular nature.

# Superficial Infection
DAGAE1 <- dagify(SSI ~ GuidanceGroup + Sex + BMI + ComorbidSmoke + ComorbidCVD + ComorbidCancer + ComorbidDiabetes,
 GuidanceGroup ~ Sex + ComorbidSmoke + ComorbidCardiovascular, #assumptions prior to group comparison
 ComorbidSmoke ~ Sex,
 ComorbidCVD ~ Sex + ComorbidSmoke + BMI,
 ComorbidDiabetes ~ Sex + ComorbidSmoke + BMI,
 labels = c(
 "SSI" = "SSI",
 "Sex" = "Sex",
 "ComorbidSmoke" = "Smoking History",
 "ComorbidCVD" = "CVD History",
 "ComorbidCancer" = "Cancer History",
 "ComorbidDiabetes" = "Diabetes History",
 "GuidanceGroup" = "Guidance Group",
 "BMI" = "Body Mass Index"
 ),
 exposure = "GuidanceGroup",
 outcome = "SSI"
)

# Create a tidy version of the DAG and apply custom labels
tidy_dag2 <- tidy_dagitty(DAGAE1, layout = "auto") |>
 mutate(label = case_when(
 name == "SSI" ~ "SSI",
 name == "ComorbidSmoke" ~ "Smoking History",
 name == "ComorbidCardiovascular" ~ "CVD History",
 name == "GuidanceGroup" ~ "GuidanceGroup",
 name == "BMI" ~ "Body Mass\n Index",
 name == "Sex" ~ "Sex",
 TRUE ~ name
 ))

Figure8 <- ggplot(
 tidy_dag2,
 aes(
 x = x,
 y = y,
 xend = xend,
 yend = yend)
 ) +
 geom_dag_node(
 color = "darkblue",
 fill = "lightblue",
 alpha = 0.5,
 size = 20) +
 geom_dag_text(
 aes(
 label = label
 ),
 color = "black",
 fontface = "bold",
 size = 3,
 vjust = 0.5
 ) +
 geom_dag_edges(
 edge_color = "gray50",
 edge_width = 0.5,
 #edge_arrow_size = 0.3
 ) +
 theme_dag(
 base_size = 12,
 base_family = "",
 plot.background = element_rect(fill = "white"),
 panel.background = element_rect(fill = "white")
 ) +
 theme(
 plot.title = element_text(
 hjust = 0.5,
 size = 16,
 face = "bold"
 ),
 plot.margin = margin(
 20,
 20,
 20,
 20
 )
 ) +
 ggtitle(
 "Directed Acyclic Graph"
 )

knitr::knit_print(Figure8)

| 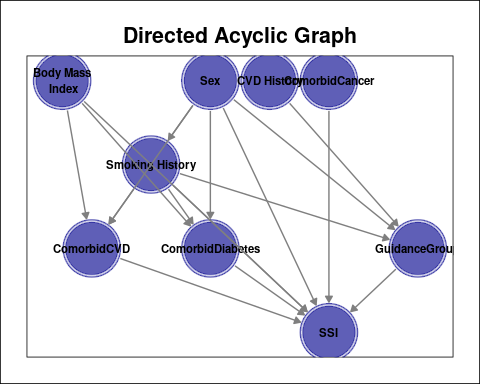  Figure 9: Directed acyclic graph of surgical site infection after total knee arthroplasty. |
| --- |

Figure9 <- ggdag_adjustment_set(
 tidy_dag2,
 text = FALSE,
 #use_labels = "label"
 exposure = "GuidanceGroup",
 outcome = "SSI"
 ) +
 geom_dag_node(
 color = "darkblue",
 fill = "lightblue",
 alpha = 0.7,
 size = 10
 ) +
 # geom_dag_text(
 # aes(label = label), # Use the 'label' column directly
 # color = "black",
 # fontface = "bold",
 # size = 3,
 # vjust = -1.5, # Adjust vertical position
 # hjust = 1.5, # Adjust horizontal position
 # show.legend = FALSE # Prevent text from appearing in legend
 # ) +
 geom_dag_label_repel(
 aes(label = label, fill = adjusted),
 color = "white",
 fontface = "bold",
 size = 3,
 show.legend = FALSE,
 box.padding = unit(0.5, "lines"), # Padding around labels
 point.padding = unit(0.5, "lines"), # Padding around points
 nudge_y = 0.5, # Adjust vertical nudge
 nudge_x = 0.5 # Adjust horizontal nudge
 ) +
 geom_dag_edges(
 edge_color = "gray50",
 edge_width = 0.5
 ) +
 theme_dag(
 base_size = 10,
 base_family = "",
 plot.background = element_rect(fill = "white"),
 panel.background = element_rect(fill = "white")
 ) +
 theme(
 plot.title = element_text(
 hjust = 0.5,
 size = 10,
 face = "bold"
 ),
 plot.margin = margin(20,20,20,20),
 legend.position = "bottom",
 legend.background = element_rect(fill = "white", color = "gray80"),
 legend.title = element_text(face = "bold")
 ) +
 ggtitle("Adjustment Set for Directed Acyclic Graph")


knitr::knit_print(Figure9)

| 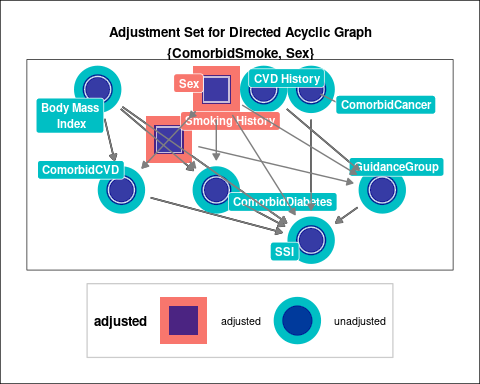  Figure 10: Calculated minimum adjustment set for modelling surgical site infection in total knee arthroplasty. |
| --- |

A literature review identified a recent meta-analysis (Zhang et al. 2015) of fourteen cohort studies (18075 VTE from 1723350 cases) that posed a number of potential factors affecting VTE risk. Oddly, this paper did not examine smoking as a factor. Smoking in general has been associated with an increased risk of VTE in observational studies (35151 events in 3966184 participants) not specific to lower limb arthroplasty (Cheng et al. 2013). Similarly, a diagnosis of diabetes has been associated with an increased risk of VTE in the general population (Deischinger et al. 2022), which may be confounded by sex.

| Table 15: Summmary of potential factors associated with venous thromboembolism (VTE) after total knee arthroplasty.   \| Variable \| Source \| Present in current dataset \| \| --- \| --- \| --- \| \| History of VTE \| Zhang et al \| ❌ \| \| Varicose vein \| Zhang et al \| ❌ \| \| Congestive heart failure \| Zhang et al \| ✅* \| \| Sex \| Zhang et al  Deischinger et al \| ✅ \| \| Age \| Zhang et al \| ✅ \| \| Active hypertension \| Zhang et al \| ✅* \| \| History of cancer \| Zhang et al \| ✅† \| \| Active diabetes \| Deischinger et al \| ✅ \| \| BMI \| Zhang et al \| ✅ \| \| Race \| Zhang et al \| ❌ \| \| History of smoking \| Cheng et al \| ✅ \| |
| --- | --- | --- | --- | --- | --- | --- | --- | --- | --- | --- | --- | --- | --- | --- | --- | --- | --- | --- | --- | --- | --- | --- | --- | --- | --- | --- | --- | --- | --- | --- | --- | --- | --- | --- | --- | --- |

*Stored in the dataset as comorbidities of a cardiovascular nature. †Stored as cancer related comorbities, combined as history or current diagnosis.

# Thromboembolism
DAGAE2 <- dagify(VTE ~ GuidanceGroup + Sex + BMI + Age + ComorbidSmoke + ComorbidCVD + ComorbidCancer + ComorbidDiabetes,

 GuidanceGroup ~ Sex + ComorbidSmoke + ComorbidCardiovascular + ComorbidDiabetes, #assumptions prior to group comparison
 ComorbidSmoke ~ Sex,
 ComorbidCVD ~ Sex + ComorbidSmoke + BMI,
 ComorbidDiabetes ~ Sex + ComorbidSmoke + BMI,
 labels = c(
 "VTE" = "VTE",
 "Sex" = "Sex",
 "ComorbidSmoke" = "Smoking History",
 "ComorbidCVD" = "CVD History",
 "ComorbidCancer" = "Cancer History",
 "ComorbidDiabetes" = "Diabetes History",
 "GuidanceGroup" = "Guidance Group",
 "Age" = "Age at\n Surgery",
 "BMI" = "Body Mass Index"
 ),
 exposure = "GuidanceGroup",
 outcome = "VTE"
)

# Create a tidy version of the DAG and apply custom labels
tidy_dag3 <- tidy_dagitty(DAGAE2, layout = "auto") |>
 mutate(label = case_when(
 name == "VTE" ~ "VTE",
 name == "Sex" ~ "Sex",
 name == "ComorbidSmoke" ~ "Smoking History",
 name == "ComorbidCVD" ~ "CVD History",
 name == "ComorbidCancer" ~ "Cancer History",
 name == "ComorbidDiabetes" ~ "Diabetes History",
 name == "GuidanceGroup" ~ "Guidance Group",
 name == "Age" ~ "Age at\n Surgery",
 name == "BMI" ~ "Body Mass Index",
 TRUE ~ name
 ))

Figure10 <- ggplot(
 tidy_dag3,
 aes(
 x = x,
 y = y,
 xend = xend,
 yend = yend)
 ) +
 geom_dag_node(
 color = "darkblue",
 fill = "lightblue",
 alpha = 0.5,
 size = 20) +
 geom_dag_text(
 aes(
 label = label
 ),
 color = "black",
 fontface = "bold",
 size = 3,
 vjust = 0.5
 ) +
 geom_dag_edges(
 edge_color = "gray50",
 edge_width = 0.5,
 #edge_arrow_size = 0.3
 ) +
 theme_dag(
 base_size = 12,
 base_family = "",
 plot.background = element_rect(fill = "white"),
 panel.background = element_rect(fill = "white")
 ) +
 theme(
 plot.title = element_text(
 hjust = 0.5,
 size = 16,
 face = "bold"
 ),
 plot.margin = margin(
 20,
 20,
 20,
 20
 )
 ) +
 ggtitle(
 "Directed Acyclic Graph"
 )

knitr::knit_print(Figure10)

| 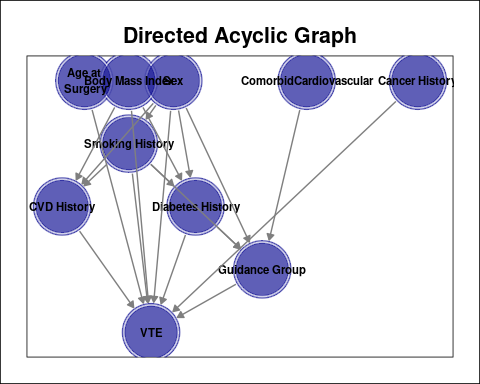  Figure 11: Directed acyclic graph of venous thromboembolism after total knee arthroplasty. |
| --- |

Figure11 <- ggdag_adjustment_set(
 tidy_dag3,
 text = FALSE,
 #use_labels = "label"
 exposure = "GuidanceGroup",
 outcome = "VTE"
 ) +
 geom_dag_node(
 color = "darkblue",
 fill = "lightblue",
 alpha = 0.7,
 size = 10
 ) +
 # geom_dag_text(
 # aes(label = label), # Use the 'label' column directly
 # color = "black",
 # fontface = "bold",
 # size = 3,
 # vjust = -1.5, # Adjust vertical position
 # hjust = 1.5, # Adjust horizontal position
 # show.legend = FALSE # Prevent text from appearing in legend
 # ) +
 geom_dag_label_repel(
 aes(label = label, fill = adjusted),
 color = "white",
 fontface = "bold",
 size = 3,
 show.legend = FALSE,
 box.padding = unit(0.5, "lines"), # Padding around labels
 point.padding = unit(0.5, "lines"), # Padding around points
 nudge_y = 0.5, # Adjust vertical nudge
 nudge_x = 0.5 # Adjust horizontal nudge
 ) +
 geom_dag_edges(
 edge_color = "gray50",
 edge_width = 0.5
 ) +
 theme_dag(
 base_size = 10,
 base_family = "",
 plot.background = element_rect(fill = "white"),
 panel.background = element_rect(fill = "white")
 ) +
 theme(
 plot.title = element_text(
 hjust = 0.5,
 size = 10,
 face = "bold"
 ),
 plot.margin = margin(20,20,20,20),
 legend.position = "bottom",
 legend.background = element_rect(fill = "white", color = "gray80"),
 legend.title = element_text(face = "bold")
 )
# +
# ggtitle("Adjustment Set for Directed Acyclic Graph")


knitr::knit_print(Figure11)

| 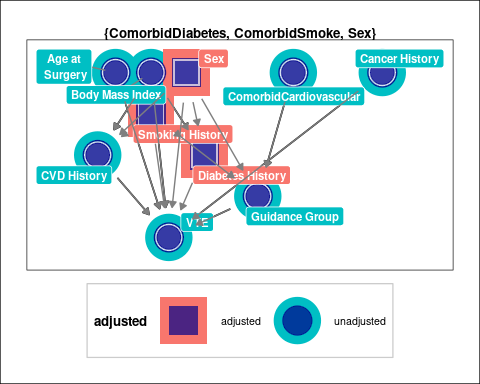  Figure 12: Calculated minimum adjustment set for modelling venous thromboembolism in total knee arthroplasty. |
| --- |

A literature review revealed a recent meta-analysis (Tibbo et al. 2019) of 35 cohort studies (48873 TKAs) and an incidence of 4% at up to 12 weeks postoperatively. There was insufficient evidence to determine the relationship between diabetes and smoking status on stiffness risk. A more recent cohort study (Owen et al. 2021) of 12735 cases also observed an incidence of 3.6% A summary of factors associated with postoperative stiffness is summarised below.

| Table 16: Summmary of potential factors associated with **stiffness** after total knee arthroplasty.   \| Variable \| Source \| Present in current dataset \| \| --- \| --- \| --- \| \| Sex \| Tibbo et al \| ✅ \| \| Age \| Tibbo et al  Owen et al \| ✅ \| \| BMI \| Tibbo et al \| ✅ \| \| Diabetes \| Owen et al \| ✅ \| |
| --- | --- | --- | --- | --- | --- | --- | --- | --- | --- | --- | --- | --- | --- | --- | --- |

# Stiffness
DAGAE3 <- dagify(Stiffness ~ GuidanceGroup + Sex + BMI + Age + ComorbidDiabetes,
 GuidanceGroup ~ Sex + ComorbidDiabetes, #assumptions prior to group comparison
 ComorbidDiabetes ~ Sex + BMI,
 labels = c(
 "Stiffness" = "Stiffness",
 "Sex" = "Sex",
 "ComorbidDiabetes" = "Diabetes History",
 "GuidanceGroup" = "Guidance Group",
 "Age" = "Age at\n Surgery",
 "BMI" = "Body Mass Index"
 ),
 exposure = "GuidanceGroup",
 outcome = "Stiffness"
)

# Create a tidy version of the DAG and apply custom labels
tidy_dag4 <- tidy_dagitty(DAGAE3, layout = "auto") |>
 mutate(label = case_when(
 name == "Stiffness" ~ "Stiffness",
 name == "Sex" ~ "Sex",
 name == "ComorbidDiabetes" ~ "Diabetes History",
 name == "GuidanceGroup" ~ "Guidance Group",
 name == "Age" ~ "Age at\n Surgery",
 name == "BMI" ~ "Body Mass Index",
 TRUE ~ name
 ))

Figure12 <- ggplot(
 tidy_dag4,
 aes(
 x = x,
 y = y,
 xend = xend,
 yend = yend)
 ) +
 geom_dag_node(
 color = "darkblue",
 fill = "lightblue",
 alpha = 0.5,
 size = 20) +
 geom_dag_text(
 aes(
 label = label
 ),
 color = "black",
 fontface = "bold",
 size = 3,
 vjust = 0.5
 ) +
 geom_dag_edges(
 edge_color = "gray50",
 edge_width = 0.5,
 #edge_arrow_size = 0.3
 ) +
 theme_dag(
 base_size = 12,
 base_family = "",
 plot.background = element_rect(fill = "white"),
 panel.background = element_rect(fill = "white")
 ) +
 theme(
 plot.title = element_text(
 hjust = 0.5,
 size = 16,
 face = "bold"
 ),
 plot.margin = margin(
 20,
 20,
 20,
 20
 )
 ) +
 ggtitle(
 "Directed Acyclic Graph"
 )

knitr::knit_print(Figure12)

| 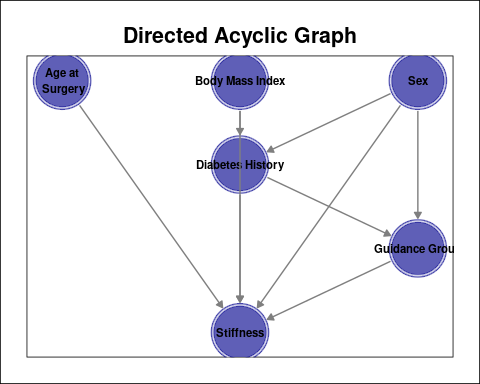  Figure 13: Directed acyclic graph of knee stiffness after total knee arthroplasty. |
| --- |

Figure13 <- ggdag_adjustment_set(
 tidy_dag4,
 text = FALSE,
 #use_labels = "label"
 exposure = "GuidanceGroup",
 outcome = "Stiffness"
 ) +
 geom_dag_node(
 color = "darkblue",
 fill = "lightblue",
 alpha = 0.7,
 size = 10
 ) +
 # geom_dag_text(
 # aes(label = label), # Use the 'label' column directly
 # color = "black",
 # fontface = "bold",
 # size = 3,
 # vjust = -1.5, # Adjust vertical position
 # hjust = 1.5, # Adjust horizontal position
 # show.legend = FALSE # Prevent text from appearing in legend
 # ) +
 geom_dag_label_repel(
 aes(label = label, fill = adjusted),
 color = "white",
 fontface = "bold",
 size = 3,
 show.legend = FALSE,
 box.padding = unit(0.5, "lines"), # Padding around labels
 point.padding = unit(0.5, "lines"), # Padding around points
 nudge_y = 0.5, # Adjust vertical nudge
 nudge_x = 0.5 # Adjust horizontal nudge
 ) +
 geom_dag_edges(
 edge_color = "gray50",
 edge_width = 0.5
 ) +
 theme_dag(
 base_size = 10,
 base_family = "",
 plot.background = element_rect(fill = "white"),
 panel.background = element_rect(fill = "white")
 ) +
 theme(
 plot.title = element_text(
 hjust = 0.5,
 size = 10,
 face = "bold"
 ),
 plot.margin = margin(20,20,20,20),
 legend.position = "bottom",
 legend.background = element_rect(fill = "white", color = "gray80"),
 legend.title = element_text(face = "bold")
 )
# +
# ggtitle("Adjustment Set for Directed Acyclic Graph")


knitr::knit_print(Figure13)

| 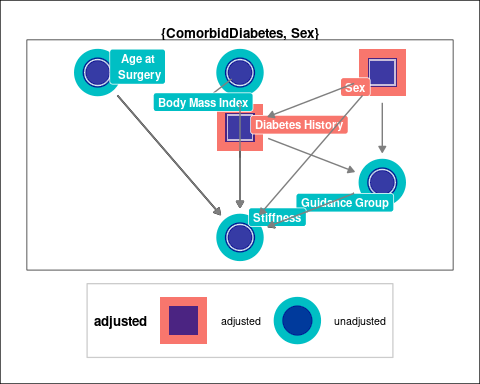  Figure 14: Calculated minimum adjustment set for modelling stiffness after total knee arthroplasty. |
| --- |

A recent systematic review (69 articles) and meta-analysis (16 articles) (Gould et al. 2021) identified a number of factors that may be associated with readmission risk after total knee arthroplasty, summarised in the table below (Table).

| Table 17: Summmary of potential factors associated with **all-cause readmission** after total knee arthroplasty.   \| Variable \| Present in current dataset \| \| --- \| --- \| \| In-hospital complication \| ✅ \| \| Peripheral vascular disease \| ✅* \| \| Liver disease \| ❌ \| \| Anaemia \| ❌ \| \| Coagulopathy \| ❌ \| \| Fluid and electrolyte disorders \| ❌ \| \| Chronic pulmonary disease \| ❌ \| \| Smoking history \| ✅ \| \| Psychiatric disorders \| ❌ \| \| Rheumatologic disorders \| ✅† \| \| Cardiovascular comorbidity \| ✅ \| \| Diabetes \| ✅ \| \| Obesity \| ✅** \| |
| --- | --- | --- | --- | --- | --- | --- | --- | --- | --- | --- | --- | --- | --- | --- | --- | --- | --- | --- | --- | --- | --- | --- | --- | --- | --- | --- | --- | --- |

†Stored in the dataset as autoimmune comorbidities. **Stored in the dataset as body mass index (BMI).

DAGAE4 <- dagify(
 ACRadmit ~ GuidanceGroup
 + BMI
 + ComorbidSmoke
 + ComorbidAutoimmune
 + ComorbidCardiovascular
 + ComorbidDiabetes,
 GuidanceGroup ~ BMI
 + ComorbidSmoke
 + ComorbidCardiovascular
 + ComorbidDiabetes, #assumptions prior to group comparison
 ComorbidSmoke ~ BMI,
 ComorbidCardiovascular ~ ComorbidDiabetes + BMI,
 ComorbidDiabetes ~ BMI,
 labels = c(
 "ACRadmit" = "Readmission",
 "GuidanceGroup" = "Guidance Group",
 "BMI" = "Body Mass Index",
 "ComorbidSmoke" = "Smoking History",
 "ComorbidAutoimmune" = "Autoimmune Disorders",
 "ComorbidCardiovascular" = "CVD",
 "ComorbidDiabetes" = "Diabetes History"

 ),
 exposure = "GuidanceGroup",
 outcome = "ACRadmit"
)

# Create a tidy version of the DAG and apply custom labels
tidy_dag5 <- tidy_dagitty(DAGAE4, layout = "auto") |>
 mutate(label = case_when(
 name == "ACRadmit" ~ "Readmission",
 name == "GuidanceGroup" ~ "Guidance Group",
 name == "BMI" ~ "Body Mass Index",
 name == "ComorbidSmoke" ~ "Smoking History",
 name == "ComorbidAutoimmune" ~ "Autoimmune Disorders",
 name == "ComorbidCardiovascular" ~ "CVD",
 name == "ComorbidDiabetes" ~ "Diabetes History",
 TRUE ~ name
 ))

Figure14 <- ggplot(
 tidy_dag5,
 aes(
 x = x,
 y = y,
 xend = xend,
 yend = yend)
 ) +
 geom_dag_node(
 color = "darkblue",
 fill = "lightblue",
 alpha = 0.5,
 size = 20) +
 geom_dag_text(
 aes(
 label = label
 ),
 color = "black",
 fontface = "bold",
 size = 3,
 vjust = 0.5
 ) +
 geom_dag_edges(
 edge_color = "gray50",
 edge_width = 0.5,
 #edge_arrow_size = 0.3
 ) +
 theme_dag(
 base_size = 12,
 base_family = "",
 plot.background = element_rect(fill = "white"),
 panel.background = element_rect(fill = "white")
 ) +
 theme(
 plot.title = element_text(
 hjust = 0.5,
 size = 16,
 face = "bold"
 ),
 plot.margin = margin(
 20,
 20,
 20,
 20
 )
 )
# +
# ggtitle(
# "Directed Acyclic Graph"
# )

knitr::knit_print(Figure14)

| 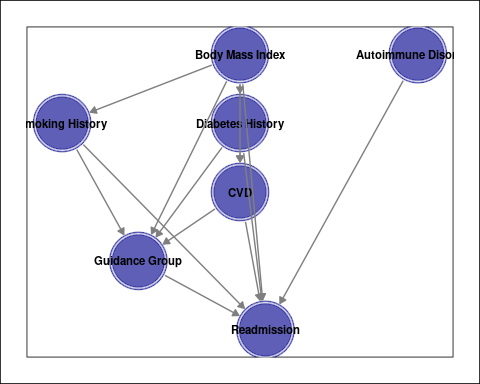  Figure 15: Directed acyclic graph of all-cause readmission after total knee arthroplasty. |
| --- |

Figure15 <- ggdag_adjustment_set(
 tidy_dag5,
 text = FALSE,
 #use_labels = "label"
 exposure = "GuidanceGroup",
 outcome = "ACRadmit"
 ) +
 geom_dag_node(
 color = "darkblue",
 fill = "lightblue",
 alpha = 0.7,
 size = 10
 ) +
 # geom_dag_text(
 # aes(label = label), # Use the 'label' column directly
 # color = "black",
 # fontface = "bold",
 # size = 3,
 # vjust = -1.5, # Adjust vertical position
 # hjust = 1.5, # Adjust horizontal position
 # show.legend = FALSE # Prevent text from appearing in legend
 # ) +
 geom_dag_label_repel(
 aes(label = label, fill = adjusted),
 color = "white",
 fontface = "bold",
 size = 3,
 show.legend = FALSE,
 box.padding = unit(0.5, "lines"), # Padding around labels
 point.padding = unit(0.5, "lines"), # Padding around points
 nudge_y = 0.5, # Adjust vertical nudge
 nudge_x = 0.5 # Adjust horizontal nudge
 ) +
 geom_dag_edges(
 edge_color = "gray50",
 edge_width = 0.5
 ) +
 theme_dag(
 base_size = 10,
 base_family = "",
 plot.background = element_rect(fill = "white"),
 panel.background = element_rect(fill = "white")
 ) +
 theme(
 plot.title = element_text(
 hjust = 0.5,
 size = 10,
 face = "bold"
 ),
 plot.margin = margin(20,20,20,20),
 legend.position = "bottom",
 legend.background = element_rect(fill = "white", color = "gray80"),
 legend.title = element_text(face = "bold")
 )
# +
# ggtitle("Adjustment Set for Directed Acyclic Graph")


knitr::knit_print(Figure15)

| 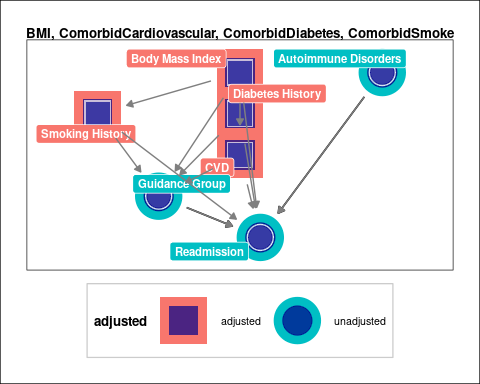  Figure 16: Calculated minimum adjustment set for modelling all-cause readmission after total knee arthroplasty. |
| --- |

The master table was subset for adverse event analysis. To account for censoring over the follow up period (90 days), adjusted cox proportional hazards models were fit for each of the key adverse events (SSI, VTE, Stiffness, All-cause Readmission) based on incidence, with the minimal adjustment set as identified in the DAGs. The cox models were fit with the *survival* package (v3.8.6 (Therneau 2024). The assumption of proportional hazards was tested for each model. A penalised spline was used to fit BMI as a continuous variable in the all-cause readmission model.

# https://stats.stackexchange.com/questions/518387/understanding-the-output-from-coxph-when-used-with-pspline

MasterSheetKM_Clean <- na.omit(MasterSheetKM)


# Superficial Infection
ModelCox1 <- survival::coxph(Surv(DurationInfectSuper,InfectionSuperficialBin) ~ Group1 + Sex + ComorbidSmoke,
 data = MasterSheetKM_Clean,
 id = PatientID2,
 cluster = PatientID2
)

#VTE
ModelCox2 <- survival::coxph(Surv(DurationThrombo,ThromboembolismBin) ~ Group1 + Sex + ComorbidSmoke + ComorbidDiabetes,
 data = MasterSheetKM_Clean,
 id = PatientID2,
 cluster = PatientID2
)

#Stiffness
ModelCox3 <- survival::coxph(Surv(DurationStiff,StiffnessBin) ~ Group1 + Sex + ComorbidDiabetes,
 data = MasterSheetKM_Clean
)

#All-cause readmission
ModelCox4 <- survival::coxph(Surv(DurationReadmit,ReadmissionBin) ~ Group1 + Sex + ComorbidSmoke + ComorbidCardiovascular + pspline(BMI, df = 3),
 data = MasterSheetKM_Clean,
 id = PatientID2,
 cluster = PatientID2
)

## 5.15 STROBE [10] Study size:

*Critically analyze the final study sample and the rationale/methods used for its justification.*

The sample size for this study was constrained by the number of cases available for analysis and meeting the inclusion criteria. For future studies a power calculation was observed of the incidence of surgical site infections using the *npsurvSS* package v(1.1.0) (Yung and Liu 2024) using multiple tests (as per below). Future studies should conduct more robust power calculations using simulated data for prospective designs, based on the results of the present analysis as inputs.

#https://stats.stackexchange.com/questions/601373/calculating-statistical-power-for-a-cox-proportional-hazards-regression-with-a-c

active <- npsurvSS::create_arm(size=258,
 accr_time=10,
 surv_scale=0.039,
 loss_scale=0,
 follow_time=90)
control <- npsurvSS::create_arm(size=173,
 accr_time = 10,
 surv_scale = 0.023,
 loss_scale = 0,
 follow_time = 90)

# SampleEx <- seq(0, 90, 0.1)
# plot(SampleEx, psurv(q=SampleEx, arm=control, lower.tail=F),
# xlab="Time from study entry (days)",
# ylab="Survival function",
# type="l")

# Your existing power analysis code
SampleSize1 <- power_two_arm(active, control,
 test=
 list(list(test="weighted logrank"),
 list(test="weighted logrank", weight="n"),
 list(test="survival difference", milestone=90),
 list(test="rmst ratio", milestone=90))
)

# Create a data frame from the results
SampleSize_df <- data.frame(
 Test = c("Weighted logrank", "Weighted logrank (n)", "Survival difference", "RMST ratio"),
 #TestNumber = SampleSize1$test,
 Power = SampleSize1$power
)

# Create the gt table
results_table <- SampleSize_df |>
 gt() |>
 tab_header(
 title = "Power Analysis Results",
 subtitle = "Two-arm survival analysis"
 ) |>
 fmt_number(
 columns = c(Power),
 decimals = 2
 ) |>
 cols_label(
 Test = "Test Type",
 #TestNumber = "Test Number",
 Power = "Power"
 ) |>
 tab_source_note(
 source_note = "Based on npsurvSS::power_two_arm() function"
 )

# gt table, cannot display using as_flex_table
knitr::knit_print(results_table)

| Table 18: Estimated power based on calculated incidences of Robot and Pre-Robot groups for superficial infection after total knee arthroplasty at 90 day follow up.  Table 1: Power Analysis Results  Two-arm survival analysis   \| Test Type \| Power \| \| --- \| --- \| \| Weighted logrank \| 1.00 \| \| Weighted logrank (n) \| 0.99 \| \| Survival difference \| 0.94 \| \| RMST ratio \| 1.00 \| \| Based on npsurvSS::power_two_arm() function \| \| |
| --- | --- | --- | --- | --- | --- | --- | --- | --- | --- | --- | --- | --- |

## 5.16 IDEAL Stage2b [5]: Learning effect

*Where possible, the effect of skill differences and learning should be assessed with appropriate data analysis*

The number of cases per surgeon by alignment delivery method were cross-tabulated for visualisation purposes, but not compared.

Table17 <- tbl_cross(
 MasterSheet6,
 row = Surgeon,
 col = Group1,
 percent = "row",
 margin = c("row")
)

as_flex_table(Table17) |> flextable::set_table_properties(
 layout = "autofit",
 width = 0.15,
 align = "center",
 opts_word = list(
 split = TRUE
 )
 )

| Table 19: Case volume by surgeon label across Groups.   \|  \| Group1 \| \| \| \| --- \| --- \| --- \| --- \| \|  \| RAS \| Pre-RAS \| Non-RAS \| \| Surgeon \|  \|  \|  \| \| A \| 10 (18%) \| 45 (82%) \| 0 (0%) \| \| B \| 44 (45%) \| 54 (55%) \| 0 (0%) \| \| C \| 96 (56%) \| 74 (44%) \| 0 (0%) \| \| D \| 0 (0%) \| 0 (0%) \| 46 (100%) \| \| E \| 31 (82%) \| 0 (0%) \| 7 (18%) \| \| F \| 25 (96%) \| 0 (0%) \| 1 (3.8%) \| \| G \| 52 (39%) \| 0 (0%) \| 83 (61%) \| \| Total \| 258 (45%) \| 173 (30%) \| 137 (24%) \| |
| --- | --- | --- | --- | --- | --- | --- | --- | --- | --- | --- | --- | --- | --- | --- | --- | --- | --- | --- | --- | --- | --- | --- | --- | --- | --- | --- | --- | --- | --- | --- | --- | --- | --- | --- | --- | --- | --- | --- | --- | --- | --- | --- | --- | --- |

The potential for a learning curve within the RAS group was visualised using graphical plots of sequential cases by surgeon.

# Within-surgeon case sequence for RAS cases only
LearningCurve_Analysis <- MasterSheet6 |>
 dplyr::filter(Group1 == "RAS") |>
 dplyr::select(Surgeon, SurgeryDate, ProcedureDuration) |>
 dplyr::arrange(Surgeon, SurgeryDate) |>
 dplyr::group_by(Surgeon) |>
 dplyr::mutate(CaseSequence = dplyr::row_number()) |>
 dplyr::ungroup()

# Case volume check per surgeon (for transparency on which panels are sparse)
# LearningCurve_Analysis |>
# dplyr::count(Surgeon, name = "RAS_n")

LearningCurveFig <- ggplot(
 data = LearningCurve_Analysis,
 mapping = aes(x = CaseSequence, y = ProcedureDuration)
) +
 geom_point(alpha = 0.5) +
 geom_smooth(method = "loess", se = TRUE, linewidth = 0.8) +
 facet_wrap(~ Surgeon, scales = "free_x") +
 labs(
 x = "Sequential RAS case number (within surgeon)",
 y = "Procedure duration (minutes)"
 ) +
 theme_minimal()

knitr::knit_print(LearningCurveFig)

| 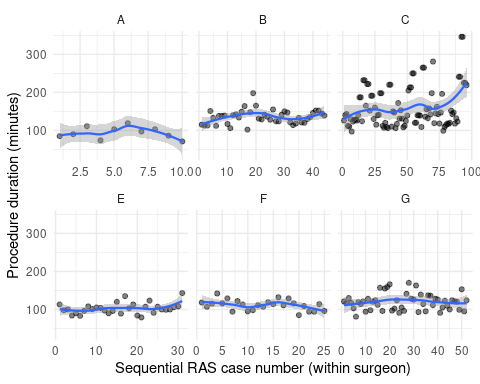  Figure 17: Within-surgeon procedure duration by sequential RAS case number |
| --- |

There were no visually discernible changes in procedure duration consistent with a learning curve within each RAS surgeon across sequential cases. A learning curve was not considered to be present within the procedure duration data.

# 6. Results:

## 6.1 STROBE [13] Participant Flow:

*Critically assess the transparency and completeness of participant flow reporting.*

A flow chart of record retrieval, review and grouping was constructed using the *consort* package v(1.2.3) (Dayim 2026).

MasterList3 <- left_join(
 MasterList2,
 MasterSheet1 |> dplyr::select(
 TempIDFull,
 ProgressComment,
 ProgressIndicator,
 PotentialExclus
 ),
 by = "TempIDFull"
) |> left_join(
 MasterSheet6 |> dplyr::select(
 TempIDFull,
 Group1
 ),
 by = "TempIDFull"
)

# Check for any mismatches
JoinCheck <- anti_join(
 MasterList3,
 MasterSheet6,
 by = "TempIDFull"
 ) |> mutate(
 Exclusion = case_when(
 #PotentialExclus == "Yes" ~ "Excluded after Review",
 SurgerySide == "Bilateral" ~ "Vendor Duplicate",
 SurgeryType != "Primary" ~ "Surgery Type",
 is.na(SurgeryDate) ~ "Cancellation|Wait List",
 Source == "Vendor" ~ "Unable to Match",
 SurgeryDate > lubridate::ymd("2023-03-01") ~ "Surgery Date",
 SurgeryDate > lubridate::ymd("2023-03-01") ~ "Surgery Date",
 .default = "Unable to Match"
 )
 )

CONSORTInput <- left_join(
 MasterList3,
 JoinCheck |> dplyr::select(
 TempIDFull,
 Exclusion
 ),
 by = "TempIDFull"
) |> rename(
 exclusion1 = "Exclusion",
 trialno = "TempIDFull",
 arm3 = "Group1"
) |> mutate(
 exclusion2 = case_when(
 PotentialExclus == "Yes" & str_detect(str_to_lower(ProgressComment),"dair|unicomp*|hemiarth*") ~ "Surgery Type",
 PotentialExclus == "Yes" & str_detect(str_to_lower(ProgressComment),"done") ~ "Cancellation",
 PotentialExclus == "Yes" & str_detect(str_to_lower(ProgressComment),"iemr|note") ~ "Record unavailable",
 PotentialExclus == "Yes" & str_detect(str_to_lower(ProgressComment),"duplicate") ~ "Record duplicate",
 .default = NA_character_
 )
) |> mutate(
 exclusion1 = ifelse(!is.na(exclusion2),NA_character_,exclusion1)
) |> mutate(
 induction = ifelse(is.na(exclusion1),trialno,NA_character_)
)

ConsortPlot <- consort::consort_plot(
 data = CONSORTInput,
 orders = c(
 trialno = "Population for Review",
 exclusion1 = "Excluded Prior\n to Chart Review",
 induction = "Chart Review",
 exclusion2 = "Excluded",
 arm3 = "Grouping"
 ),
 side_box = c(
 "exclusion1",
 "exclusion2"
 ),
 allocation = "arm3",
 labels = c(
 "1" = "Record ingestion",
 "2" = "Review",
 "3" = "Grouping"),
 cex = 0.7
)

plot(ConsortPlot)

| 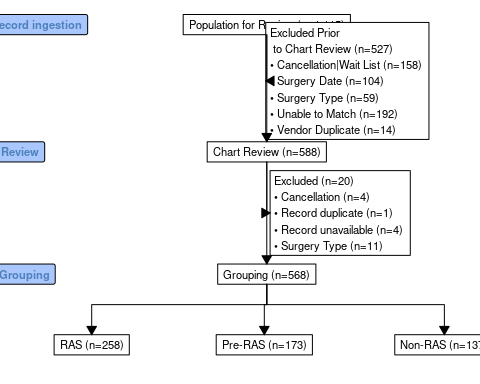  Figure 18: Flowchart of record ingestion and review. |
| --- |

## 6.2 STROBE [14] Descriptive Data:

*Evaluate the adequacy of the presentation of characteristics of the study population.*

TableChar <- tbl_summary(
 MasterSheet6 |> dplyr::select(
 Group1,
 Sex,
 AgeAtSurgery,
 BMI,
 ComorbidBone,
 ComorbidAutoImmune,
 ComorbidCancer,
 ComorbidCardiovascular,
 ComorbidDiabetes,
 ComorbidNeuro,
 BilateralStatus,


 ),
 by = Group1,
 missing = "no"
) |> add_overall()

as_flex_table(TableChar) |> flextable::set_table_properties(
 layout = "autofit",
 width = 0.15,
 align = "center",
 opts_word = list(
 split = TRUE
 )
 )

| Table 20: Patient characteristics of analysis sample separated by Group.   \| **Characteristic** \| **Overall**  N = 568^1^ \| **RAS**  N = 258^1^ \| **Pre-RAS**  N = 173^1^ \| **Non-RAS**  N = 137^1^ \| \| --- \| --- \| --- \| --- \| --- \| \| Sex \|  \|  \|  \|  \| \| Female \| 322 (57%) \| 141 (55%) \| 93 (54%) \| 88 (64%) \| \| Male \| 246 (43%) \| 117 (45%) \| 80 (46%) \| 49 (36%) \| \| AgeAtSurgery \| 69 (62, 76) \| 69 (64, 76) \| 68 (61, 75) \| 70 (63, 77) \| \| BMI \| 32.2 (28.1, 36.8) \| 32.3 (28.5, 36.8) \| 32.0 (27.6, 37.1) \| 32.3 (28.3, 35.9) \| \| ComorbidBone \| 25 (4.4%) \| 8 (3.1%) \| 7 (4.0%) \| 10 (7.3%) \| \| ComorbidAutoImmune \| 22 (3.9%) \| 9 (3.5%) \| 9 (5.2%) \| 4 (2.9%) \| \| ComorbidCancer \| 13 (2.3%) \| 2 (0.8%) \| 0 (0%) \| 11 (8.0%) \| \| ComorbidCardiovascular \| 16 (2.8%) \| 9 (3.5%) \| 1 (0.6%) \| 6 (4.4%) \| \| ComorbidDiabetes \| 86 (15%) \| 43 (17%) \| 25 (14%) \| 18 (13%) \| \| ComorbidNeuro \| 1 (0.2%) \| 1 (0.4%) \| 0 (0%) \| 0 (0%) \| \| BilateralStatus \|  \|  \|  \|  \| \| Bilateral \| 204 (36%) \| 79 (31%) \| 69 (40%) \| 56 (41%) \| \| Unilateral \| 364 (64%) \| 179 (69%) \| 104 (60%) \| 81 (59%) \| \| ^1^n (%); Median (Q1, Q3) \| \| \| \| \| |
| --- | --- | --- | --- | --- | --- | --- | --- | --- | --- | --- | --- | --- | --- | --- | --- | --- | --- | --- | --- | --- | --- | --- | --- | --- | --- | --- | --- | --- | --- | --- | --- | --- | --- | --- | --- | --- | --- | --- | --- | --- | --- | --- | --- | --- | --- | --- | --- | --- | --- | --- | --- | --- | --- | --- | --- | --- | --- | --- | --- | --- | --- | --- | --- | --- | --- | --- | --- | --- | --- | --- | --- | --- | --- | --- | --- | --- | --- | --- | --- | --- |

TableSurgMain <- tbl_summary(
 MasterSheet6 |> dplyr::select(
 SurgerySide,
 Group1,
 BilateralStatus2,
 Surgery_ProcedureName,
 Operator2,
 Approach2,
 SoftTissueRelease,
 Anaesthetic3,
 TourniquetType2,
 Pathology,
 AlignmentReferencing,
 SoftTissueRelease,
 FemurBrand2,
 FemurFixation2,
 TibiaFixation2,
 PatellaIntervention,
 Constraint2
 ),
 label = list(
 BilateralStatus2 ~ "Bilateral Status",
 Operator2 ~ "Operator",
 Approach2 ~ "Approach",
 Anaesthetic3 ~ "Anaesthetic",
 TourniquetType2 ~ "Tourniquet Use",
 FemurBrand2 ~ "Femur Brand",
 FemurFixation2 ~ "Femur Fixation",
 TibiaFixation2 ~ "Tibia Fixation",
 Constraint2 ~ "Prosthesis Constraint",
 PatellaIntervention ~ "Patella Resurfaced"
 ),
 type = list(
 PatellaIntervention ~ "dichotomous"
 ),
 value = list(
 PatellaIntervention ~ "Resurfaced"
 ),
 by = Group1
) |> add_overall()

as_flex_table(Table6) |> flextable::set_table_properties(
 layout = "autofit",
 width = 0.15,
 align = "center",
 opts_word = list(
 split = TRUE
 )
 )

| Table 21: Surgery characteristics of analysis sample separated by Group.   \| **Characteristic** \| **Overall**  N = 568^1^ \| **Non-RAS**  N = 137^1^ \| **Pre-RAS**  N = 173^1^ \| **RAS**  N = 258^1^ \| \| --- \| --- \| --- \| --- \| --- \| \| SurgerySide \|  \|  \|  \|  \| \| Left \| 278 (49%) \| 59 (43%) \| 88 (51%) \| 131 (51%) \| \| Right \| 290 (51%) \| 78 (57%) \| 85 (49%) \| 127 (49%) \| \| Bilateral Status \|  \|  \|  \|  \| \| Simultaneous \| 53 (9.3%) \| 8 (5.8%) \| 20 (12%) \| 25 (9.7%) \| \| Staged \| 149 (26%) \| 46 (34%) \| 49 (28%) \| 54 (21%) \| \| Unilateral \| 366 (64%) \| 83 (61%) \| 104 (60%) \| 179 (69%) \| \| Surgery_ProcedureName \|  \|  \|  \|  \| \| Kap1 - Primary Total Arthroplasty (Unilateral) \| 376 (68%) \| 84 (61%) \| 115 (68%) \| 177 (72%) \| \| Kap2 - Primary Total Arthroplasty (Bilateral) \| 63 (11%) \| 12 (8.8%) \| 20 (12%) \| 31 (13%) \| \| Kap2.2 - Primary Total Arthroplasty (Subsequent Bilateral) \| 113 (20%) \| 41 (30%) \| 33 (20%) \| 39 (16%) \| \| Unknown \| 16 \| 0 \| 5 \| 11 \| \| Operator \|  \|  \|  \|  \| \| Consultant \| 402 (72%) \| 79 (58%) \| 122 (73%) \| 201 (78%) \| \| Resident \| 158 (28%) \| 58 (42%) \| 44 (27%) \| 56 (22%) \| \| Unknown \| 8 \| 0 \| 7 \| 1 \| \| Approach \|  \|  \|  \|  \| \| Lateral Parapatellar \| 30 (5.3%) \| 0 (0%) \| 12 (7.1%) \| 18 (7.0%) \| \| Medial Parapatellar \| 531 (94%) \| 137 (100%) \| 155 (92%) \| 239 (93%) \| \| Medial Parapatellar, Quads Snip \| 1 (0.2%) \| 0 (0%) \| 1 (0.6%) \| 0 (0%) \| \| Other \| 1 (0.2%) \| 0 (0%) \| 1 (0.6%) \| 0 (0%) \| \| Unknown \| 5 \| 0 \| 4 \| 1 \| \| Anaesthetic \|  \|  \|  \|  \| \| Epidural \| 5 (0.9%) \| 0 (0%) \| 4 (2.4%) \| 1 (0.4%) \| \| Epidural, Sedation \| 1 (0.2%) \| 0 (0%) \| 1 (0.6%) \| 0 (0%) \| \| General \| 100 (18%) \| 28 (20%) \| 27 (16%) \| 45 (18%) \| \| General, Epidural \| 6 (1.1%) \| 4 (2.9%) \| 0 (0%) \| 2 (0.8%) \| \| General, Regional Block \| 5 (0.9%) \| 2 (1.5%) \| 3 (1.8%) \| 0 (0%) \| \| General, Regional Block, Spinal \| 3 (0.5%) \| 3 (2.2%) \| 0 (0%) \| 0 (0%) \| \| General, Spinal \| 82 (15%) \| 10 (7.3%) \| 27 (16%) \| 45 (18%) \| \| General, Spinal, Epidural \| 1 (0.2%) \| 0 (0%) \| 0 (0%) \| 1 (0.4%) \| \| General, Spinal, Sedation \| 1 (0.2%) \| 0 (0%) \| 1 (0.6%) \| 0 (0%) \| \| Regional Block, Epidural \| 1 (0.2%) \| 0 (0%) \| 1 (0.6%) \| 0 (0%) \| \| Regional Block, Spinal \| 5 (0.9%) \| 1 (0.7%) \| 4 (2.4%) \| 0 (0%) \| \| Regional Block, Spinal, Sedation \| 3 (0.5%) \| 0 (0%) \| 3 (1.8%) \| 0 (0%) \| \| Spinal \| 230 (41%) \| 57 (42%) \| 59 (35%) \| 114 (44%) \| \| Spinal, Epidural \| 7 (1.2%) \| 0 (0%) \| 2 (1.2%) \| 5 (1.9%) \| \| Spinal, Sedation \| 113 (20%) \| 32 (23%) \| 37 (22%) \| 44 (17%) \| \| Unknown \| 5 \| 0 \| 4 \| 1 \| \| Tourniquet Use \|  \|  \|  \|  \| \| Intermittent \| 45 (8.2%) \| 13 (9.5%) \| 6 (3.8%) \| 26 (10%) \| \| Not Used \| 88 (16%) \| 9 (6.6%) \| 43 (27%) \| 36 (14%) \| \| Released After Closure \| 46 (8.4%) \| 1 (0.7%) \| 26 (17%) \| 19 (7.5%) \| \| Released Before Closure \| 369 (67%) \| 114 (83%) \| 82 (52%) \| 173 (68%) \| \| Unknown \| 20 \| 0 \| 16 \| 4 \| \| Pathology \|  \|  \|  \|  \| \| Inflammatory Arthritis \| 2 (0.4%) \| 0 (0%) \| 1 (0.6%) \| 1 (0.4%) \| \| Osteoarthritis \| 555 (99%) \| 136 (99%) \| 165 (98%) \| 254 (99%) \| \| Osteonecrosis \| 1 (0.2%) \| 0 (0%) \| 1 (0.6%) \| 0 (0%) \| \| Trauma \| 4 (0.7%) \| 1 (0.7%) \| 2 (1.2%) \| 1 (0.4%) \| \| Unknown \| 6 \| 0 \| 4 \| 2 \| \| AlignmentReferencing \|  \|  \|  \|  \| \| Im Femur/Em Tibia \| 116 (21%) \| 93 (68%) \| 23 (14%) \| 0 (0%) \| \| Navigation \| 183 (33%) \| 43 (32%) \| 140 (86%) \| 0 (0%) \| \| Robotic \| 254 (46%) \| 0 (0%) \| 0 (0%) \| 254 (100%) \| \| Unknown \| 15 \| 1 \| 10 \| 4 \| \| SoftTissueRelease \|  \|  \|  \|  \| \| Combined Release/Tighten \| 1 (0.2%) \| 1 (0.7%) \| 0 (0%) \| 0 (0%) \| \| Extensive Lateral \| 10 (1.8%) \| 0 (0%) \| 4 (2.4%) \| 6 (2.4%) \| \| Extensive Medial \| 27 (4.9%) \| 1 (0.7%) \| 12 (7.3%) \| 14 (5.5%) \| \| Minimal Lateral \| 29 (5.2%) \| 0 (0%) \| 21 (13%) \| 8 (3.2%) \| \| Minimal Medial \| 403 (73%) \| 124 (91%) \| 74 (45%) \| 205 (81%) \| \| Minimal Medial, Itb Pie Crust \| 4 (0.7%) \| 4 (2.9%) \| 0 (0%) \| 0 (0%) \| \| None \| 75 (14%) \| 6 (4.4%) \| 49 (30%) \| 20 (7.9%) \| \| Other \| 5 (0.9%) \| 0 (0%) \| 5 (3.0%) \| 0 (0%) \| \| Unknown \| 14 \| 1 \| 8 \| 5 \| \| Femur Brand \|  \|  \|  \|  \| \| Attune \| 51 (9.3%) \| 51 (37%) \| 0 (0%) \| 0 (0%) \| \| Genesis \| 18 (3.3%) \| 18 (13%) \| 0 (0%) \| 0 (0%) \| \| Nexgen \| 38 (6.9%) \| 2 (1.5%) \| 36 (23%) \| 0 (0%) \| \| Persona \| 392 (71%) \| 14 (10%) \| 121 (77%) \| 257 (100%) \| \| RHK \| 2 (0.4%) \| 2 (1.5%) \| 0 (0%) \| 0 (0%) \| \| Triathlon \| 50 (9.1%) \| 50 (36%) \| 0 (0%) \| 0 (0%) \| \| Unknown \| 17 \| 0 \| 16 \| 1 \| \| Femur Fixation \|  \|  \|  \|  \| \| Cement \| 347 (63%) \| 84 (62%) \| 109 (69%) \| 154 (60%) \| \| Cementless \| 203 (37%) \| 52 (38%) \| 49 (31%) \| 102 (40%) \| \| Unknown \| 18 \| 1 \| 15 \| 2 \| \| Tibia Fixation \|  \|  \|  \|  \| \| Cement \| 459 (83%) \| 92 (67%) \| 144 (89%) \| 223 (88%) \| \| Cementless \| 92 (17%) \| 45 (33%) \| 17 (11%) \| 30 (12%) \| \| Unknown \| 17 \| 0 \| 12 \| 5 \| \| Patella Resurfaced \| 429 (76%) \| 80 (58%) \| 124 (73%) \| 225 (88%) \| \| Unknown \| 5 \| 0 \| 4 \| 1 \| \| Prosthesis Constraint \|  \|  \|  \|  \| \| Constrained Condylar \| 5 (0.9%) \| 2 (1.5%) \| 2 (1.3%) \| 1 (0.4%) \| \| Cruciate Retaining \| 466 (84%) \| 118 (86%) \| 126 (79%) \| 222 (87%) \| \| Medial Pivot \| 34 (6.2%) \| 0 (0%) \| 14 (8.8%) \| 20 (7.8%) \| \| Other \| 18 (3.3%) \| 7 (5.1%) \| 6 (3.8%) \| 5 (2.0%) \| \| Posterior Stabilised \| 29 (5.3%) \| 10 (7.3%) \| 11 (6.9%) \| 8 (3.1%) \| \| Unknown \| 16 \| 0 \| 14 \| 2 \| \| ^1^n (%) \| \| \| \| \| |
| --- | --- | --- | --- | --- | --- | --- | --- | --- | --- | --- | --- | --- | --- | --- | --- | --- | --- | --- | --- | --- | --- | --- | --- | --- | --- | --- | --- | --- | --- | --- | --- | --- | --- | --- | --- | --- | --- | --- | --- | --- | --- | --- | --- | --- | --- | --- | --- | --- | --- | --- | --- | --- | --- | --- | --- | --- | --- | --- | --- | --- | --- | --- | --- | --- | --- | --- | --- | --- | --- | --- | --- | --- | --- | --- | --- | --- | --- | --- | --- | --- | --- | --- | --- | --- | --- | --- | --- | --- | --- | --- | --- | --- | --- | --- | --- | --- | --- | --- | --- | --- | --- | --- | --- | --- | --- | --- | --- | --- | --- | --- | --- | --- | --- | --- | --- | --- | --- | --- | --- | --- | --- | --- | --- | --- | --- | --- | --- | --- | --- | --- | --- | --- | --- | --- | --- | --- | --- | --- | --- | --- | --- | --- | --- | --- | --- | --- | --- | --- | --- | --- | --- | --- | --- | --- | --- | --- | --- | --- | --- | --- | --- | --- | --- | --- | --- | --- | --- | --- | --- | --- | --- | --- | --- | --- | --- | --- | --- | --- | --- | --- | --- | --- | --- | --- | --- | --- | --- | --- | --- | --- | --- | --- | --- | --- | --- | --- | --- | --- | --- | --- | --- | --- | --- | --- | --- | --- | --- | --- | --- | --- | --- | --- | --- | --- | --- | --- | --- | --- | --- | --- | --- | --- | --- | --- | --- | --- | --- | --- | --- | --- | --- | --- | --- | --- | --- | --- | --- | --- | --- | --- | --- | --- | --- | --- | --- | --- | --- | --- | --- | --- | --- | --- | --- | --- | --- | --- | --- | --- | --- | --- | --- | --- | --- | --- | --- | --- | --- | --- | --- | --- | --- | --- | --- | --- | --- | --- | --- | --- | --- | --- | --- | --- | --- | --- | --- | --- | --- | --- | --- | --- | --- | --- | --- | --- | --- | --- | --- | --- | --- | --- | --- | --- | --- | --- | --- | --- | --- | --- | --- | --- | --- | --- | --- | --- | --- | --- | --- | --- | --- | --- | --- | --- | --- | --- | --- | --- | --- | --- | --- | --- | --- | --- | --- | --- | --- | --- | --- | --- | --- | --- | --- | --- | --- | --- | --- | --- | --- | --- | --- | --- | --- | --- | --- | --- | --- | --- | --- | --- | --- | --- | --- | --- | --- | --- | --- | --- | --- | --- | --- | --- | --- | --- | --- | --- | --- | --- | --- | --- | --- | --- | --- | --- | --- | --- | --- | --- | --- | --- | --- | --- | --- | --- | --- | --- | --- | --- | --- | --- | --- | --- | --- | --- | --- | --- | --- | --- | --- | --- | --- | --- | --- | --- | --- | --- | --- | --- | --- | --- | --- | --- | --- | --- | --- | --- | --- | --- | --- | --- | --- | --- | --- | --- | --- | --- | --- | --- | --- | --- | --- | --- | --- | --- | --- | --- | --- | --- | --- | --- | --- | --- | --- | --- | --- | --- | --- | --- | --- | --- | --- | --- | --- | --- | --- | --- | --- |

## 6.3 STROBE [15] Outcome Data:

*Evaluate the adequacy of the presentation of outcome data (over time).*

TableOutcomes <- tbl_summary(
 MasterSheet6 |> dplyr::select(
 Group1,
 ProcedureDuration,
 Bleed:Revision
 ),
 by = Group1,
 missing = "no"
) |> add_overall()

as_flex_table(TableOutcomes) |> flextable::set_table_properties(
 layout = "autofit",
 width = 0.15,
 align = "center",
 opts_word = list(
 split = TRUE
 )
 )

| Table 22: Summary of procedure duration and adverse events separated by Group.   \| **Characteristic** \| **Overall**  N = 568^1^ \| **RAS**  N = 258^1^ \| **Pre-RAS**  N = 173^1^ \| **Non-RAS**  N = 137^1^ \| \| --- \| --- \| --- \| --- \| --- \| \| ProcedureDuration \| 124 (107, 144) \| 123 (108, 143) \| 128 (110, 151) \| 120 (103, 142) \| \| Bleed \| 7 (1.2%) \| 3 (1.2%) \| 1 (0.6%) \| 3 (2.2%) \| \| Thromboembolism \| 15 (2.6%) \| 5 (1.9%) \| 6 (3.5%) \| 4 (2.9%) \| \| Neural \| 4 (0.7%) \| 1 (0.4%) \| 2 (1.2%) \| 1 (0.7%) \| \| Vascular \| 1 (0.2%) \| 1 (0.4%) \| 0 (0%) \| 0 (0%) \| \| Stiffness \| 9 (1.6%) \| 7 (2.7%) \| 0 (0%) \| 2 (1.5%) \| \| InfectionSuperficial \| 18 (3.2%) \| 10 (3.9%) \| 4 (2.3%) \| 4 (2.9%) \| \| InfectionDeep \| 1 (0.2%) \| 0 (0%) \| 0 (0%) \| 1 (0.7%) \| \| InfectionSyst \| 2 (0.4%) \| 0 (0%) \| 1 (0.6%) \| 1 (0.7%) \| \| PeriprostheticFracture \| 3 (0.5%) \| 0 (0%) \| 1 (0.6%) \| 2 (1.5%) \| \| Patellofemoral \| 3 (0.5%) \| 1 (0.4%) \| 2 (1.2%) \| 0 (0%) \| \| Delivery \| 4 (0.7%) \| 4 (1.6%) \| 0 (0%) \| 0 (0%) \| \| Readmission \| 38 (6.7%) \| 16 (6.2%) \| 8 (4.6%) \| 14 (10%) \| \| Reoperation \| 24 (4.2%) \| 12 (4.7%) \| 4 (2.3%) \| 8 (5.8%) \| \| Revision \| 2 (0.4%) \| 0 (0%) \| 0 (0%) \| 2 (1.5%) \| \| ^1^Median (Q1, Q3); n (%) \| \| \| \| \| |
| --- | --- | --- | --- | --- | --- | --- | --- | --- | --- | --- | --- | --- | --- | --- | --- | --- | --- | --- | --- | --- | --- | --- | --- | --- | --- | --- | --- | --- | --- | --- | --- | --- | --- | --- | --- | --- | --- | --- | --- | --- | --- | --- | --- | --- | --- | --- | --- | --- | --- | --- | --- | --- | --- | --- | --- | --- | --- | --- | --- | --- | --- | --- | --- | --- | --- | --- | --- | --- | --- | --- | --- | --- | --- | --- | --- | --- | --- | --- | --- | --- | --- | --- | --- | --- | --- |

The RAS group displayed 4 (1.6%) instances of the system failing intraoperatively.

DeliveryComp <- ComplicResponse7 |> filter(
 Category_Value == "Delivery"
) |> dplyr::select(
 SurgeryDate,
 ComplicationNature
) |> gt::gt()

knitr::knit_print(DeliveryComp)

| Table 23: Complications identified intraoperatively with RAS delivery.   \| SurgeryDate \| ComplicationNature \| \| --- \| --- \| \| 2022-05-11 \| Op note states 'technical difficulties with ROSA intermittently throughout case'. No further elaboration \| \| 2022-11-30 \| ROSA malfunction after tibial resection & first femoral cut --> unable to use ROSA for rest of resections or balancing --> converted to instrumented set. Op note states ' ROSA robot lost herself' - nil further description of issues. \| \| 2022-07-01 \| ROSA camera not registering --> converted to nav \| \| 2022-09-14 \| Extensive op time as difficulty achieving adequate extension gap with ROSA. decision made to increase constraint to CCK prosthesis \| |
| --- | --- | --- | --- | --- | --- | --- | --- | --- | --- | --- |

## 6.4 STROBE [16] Main Results:

*Critically analyze the presentation and interpretation of primary findings, including effect estimates and confidence intervals.*

### 6.4.1 Procedure Duration

model_data <- data.frame(
 fitted = fitted(Duration_model),
 residuals = resid(Duration_model)
)

# Create the ggplot
Durationp1 <- ggplot(model_data, aes(x = fitted, y = residuals)) +
 geom_point() +
 geom_hline(yintercept = 0, linetype = "dashed", color = "red") +
 geom_smooth(se = FALSE, method = "loess", color = "red") +
 labs(x = "Fitted values",
 y = "Residuals",
 title = "Residuals vs Fitted") +
 theme_minimal()


knitr::knit_print(Durationp1)

| 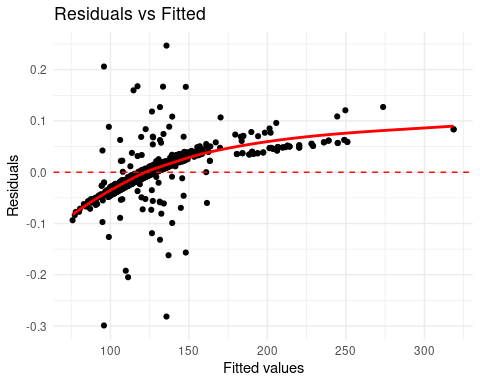  Figure 19: Summary of gamma regression residuals for procedure duration. |
| --- |

# Assuming m1 is your model
residuals <- resid(Duration_model)

# Create a data frame with theoretical and sample quantiles
qq_data <- qqplot(qnorm(ppoints(length(residuals))),
 residuals,
 plot.it = FALSE)

# Function to calculate qqline
qqline_data <- function(y, probs = c(0.25, 0.75), qtype = 7) {
 y <- quantile(y, probs, type = qtype, names = FALSE)
 x <- qnorm(probs)
 slope <- diff(y) / diff(x)
 int <- y[1L] - slope * x[1L]
 data.frame(intercept = int, slope = slope)
}

line_data <- qqline_data(residuals)

# Create the ggplot
Durationp2 <- ggplot(data.frame(x = qq_data$x, y = qq_data$y), aes(x = x, y = y)) +
 geom_point() +
 geom_abline(data = line_data,
 aes(intercept = intercept, slope = slope),
 color = "red", linetype = "dashed") +
 labs(x = "Theoretical Quantiles",
 y = "Sample Quantiles",
 title = "Normal Q-Q Plot") +
 theme_minimal()

knitr::knit_print(Durationp2)

| 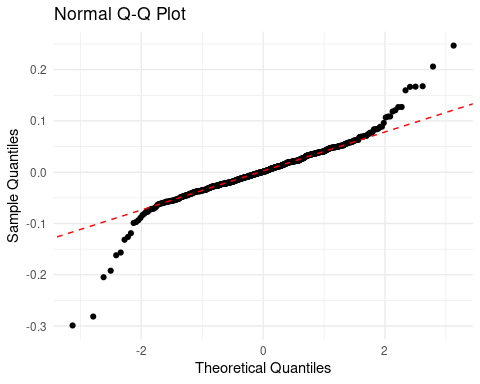  Figure 20: Summary of gamma regression leverage points (qq plot) for procedure duration. |
| --- |

The residual fits and leverage points for the gamma regression were outside acceptable patterns (see STROBE 17 for further details). The model appeared to suffer from non-linear patterns of residuals, heteroscedasticity and outliers (Figure 19 and Figure 20). Further work may be required to refine the model further to establish a better fit to the observed data.

TableDuration <- tbl_regression(Duration_model,
 show_single_row = "BilateralStatus",
 label = list(BilateralStatus ~ "Bilateral"
 #AgeAtSurgery ~ "Age at Surgery",
 #Pathology2 ~ "Osteoarthritis"
 ),
 estimate_fun = function(x) style_number(x, digits = 2), pvalue_fun = function(x) style_pvalue(x, digits = 3))

as_flex_table(TableDuration) |> flextable::set_table_properties(
 layout = "autofit",
 width = 0.15,
 align = "center",
 opts_word = list(
 split = TRUE
 )
 )

| Table 24: Summary of gamma regression model to assess the effect of Group on procedure duration   \| **Characteristic** \| **Beta** \| **95% CI** \| **p-value** \| \| --- \| --- \| --- \| --- \| \| Group1 \|  \|  \|  \| \| RAS \| — \| — \|  \| \| Pre-RAS \| 0.05 \| 0.02, 0.09 \| 0.004 \| \| Non-RAS \| -0.03 \| -0.08, 0.03 \| 0.319 \| \| Bilateral \| -0.02 \| -0.07, 0.03 \| 0.469 \| \| Abbreviation: CI = Confidence Interval \| \| \| \| |
| --- | --- | --- | --- | --- | --- | --- | --- | --- | --- | --- | --- | --- | --- | --- | --- | --- | --- | --- | --- | --- | --- | --- | --- | --- | --- | --- | --- | --- |

The model identified a significant difference in procedure duration between the RAS and Pre-RAS groups, but not the Non-RAS group ( Table 24).

#marginaleffects package

DurationPredict <- marginaleffects::predictions(
 Duration_model,
 re.form = NA,
 type = "response",
 conf_level = 0.95)

DurationObserved <- MasterSheet6 |>
 select(Group1, ProcedureDuration) |>
 tidyr::drop_na()

DurationFig <- ggplot() +
 stat_slab(
 data = DurationPredict,
 mapping = aes(y = Group1, x = ProcedureDuration,
 thickness = after_stat(pdf * n), fill = "Predicted"),
 side = "top", scale = 0.7, alpha = 0.7
 ) +
 stat_slab(
 data = DurationObserved,
 mapping = aes(y = Group1, x = ProcedureDuration,
 thickness = after_stat(pdf * n), fill = "Observed"),
 side = "bottom", scale = 0.7, alpha = 0.7
 ) +
 stat_dotsinterval(
 data = DurationObserved,
 mapping = aes(y = Group1, x = ProcedureDuration,
 fill = "Observed", colour = "Observed"),
 side = "bottom", scale = 0.7, slab_linewidth = NA
 ) +
 scale_fill_manual(name = "Data source", values = c(Predicted = "#3182bd", Observed = "#de2d26")) +
 scale_colour_manual(name = "Data source", values = c(Predicted = "#3182bd", Observed = "#de2d26")) +
 guides(colour = "none")

knitr::knit_print(DurationFig)

| 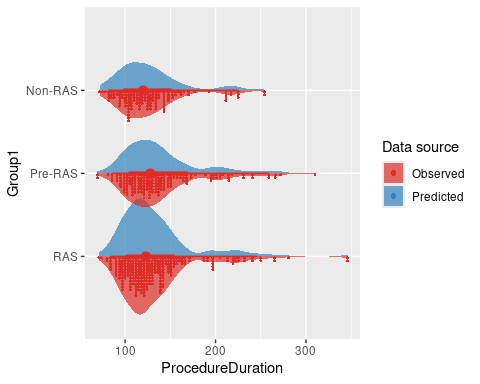  Figure 21: Distribution of model-predicted procedure duration separated by Group. |
| --- |

# --- Step 2: complete-case observed dataset ---
DurationObserved <- MasterSheet6 |>
 select(Group1, ProcedureDuration) |>
 tidyr::drop_na()

epoxy::epoxy(
 "Complete-case observed n = {nrow(DurationObserved)} of {nrow(MasterSheet6)} total rows in MasterSheet6."
)

Complete-case observed n = 566 of 568 total rows in MasterSheet6.

The model-predicted procedure duration Figure 21 conformed with the patterns from the observed data, including the extreme values.

DurationPost <- avg_comparisons(
 Duration_model,
 variables = list(Group1 = "pairwise"),
 type = "response",
 re.form = NA) |> gt::gt() |>
 fmt_number(
 decimals = 2
 )


knitr::knit_print(DurationPost) #gt object, cant be displayed as_flex_table

| Table 25: Post-hoc comparisons between Groups for procedure duration.   \| term \| contrast \| estimate \| std.error \| statistic \| p.value \| s.value \| conf.low \| conf.high \| \| --- \| --- \| --- \| --- \| --- \| --- \| --- \| --- \| --- \| \| Group1 \| Non-RAS - Pre-RAS \| -9.68 \| 3.28 \| -2.95 \| 0.00 \| 8.29 \| -16.11 \| -3.24 \| \| Group1 \| Non-RAS - RAS \| -3.13 \| 3.13 \| -1.00 \| 0.32 \| 1.66 \| -9.27 \| 3.00 \| \| Group1 \| Pre-RAS - RAS \| 6.55 \| 2.27 \| 2.88 \| 0.00 \| 7.99 \| 2.10 \| 11.00 \| |
| --- | --- | --- | --- | --- | --- | --- | --- | --- | --- | --- | --- | --- | --- | --- | --- | --- | --- | --- | --- | --- | --- | --- | --- | --- | --- | --- | --- | --- | --- | --- | --- | --- | --- | --- | --- | --- |

# Calculate marginal means
TableDurationSum <- avg_predictions(Duration_model,
 by = "Group1",
 re.form = NA,
 conf_level = 0.95,
 type = "response") |> mutate(
 standarddev = case_when(
 Group1 == "Pre-RAS" ~ std.error*(sqrt(nrow(Duration_Analysis |> filter(Group1 == "Pre-RAS")))),
 Group1 == "Non-RAS" ~ std.error*(sqrt(nrow(Duration_Analysis |> filter(Group1 == "Non-RAS")))),
 Group1 == "RAS" ~ std.error*(sqrt(nrow(Duration_Analysis |> filter(Group1 == "RAS")))),
 )
 ) |> dplyr::select(
 Group1,
 estimate,
 standarddev,
 std.error,
 conf.low,
 conf.high
 ) |> gt::gt() |>
 fmt_number(
 decimals = 1
 )

knitr::knit_print(TableDurationSum)

| Table 26: Model-predictedprocedure duration by Group   \| Group1 \| estimate \| standarddev \| std.error \| conf.low \| conf.high \| \| --- \| --- \| --- \| --- \| --- \| --- \| \| RAS \| 121.9 \| 34.8 \| 2.2 \| 117.6 \| 126.1 \| \| Pre-RAS \| 128.2 \| 33.9 \| 2.6 \| 123.2 \| 133.3 \| \| Non-RAS \| 118.5 \| 32.7 \| 2.8 \| 113.1 \| 124.0 \| |
| --- | --- | --- | --- | --- | --- | --- | --- | --- | --- | --- | --- | --- | --- | --- | --- | --- | --- | --- | --- | --- | --- | --- | --- | --- |

The beta coefficient for the Non-RAS compared to the RAS group -0.03 (95% CI -0.08 - 0.03],p=0.3) was not significant. Similarly, cases with a unilateral procedure were not significantly shorter than bilateral, -0.02 (95% CI -0.07 - 0.03),p=0.5). This translated to an increase of 3.1 minutes (3 - 9.3, p = 0.317) for RAS cases compared to Non-RAS. However, a significant decrease was observed in the average procedure duration of 6.5 (2.1 - 11, p = 0.004) minutes per case for RAS cases, compared to Pre-RAS (Table 26).

### 6.4.2 Adverse Events

TableComp <- tbl_summary(
 data = MasterSheet6 |> dplyr::select(
 Group1,
 Bleed,
 Thromboembolism,
 Neural,
 Vascular,
 Stiffness,
 InfectionSuperficial,
 InfectionDeep,
 InfectionSyst,
 PeriprostheticFracture,
 Patellofemoral,
 Delivery,
 Readmission,
 Reoperation,
 Revision

 ),
 by = Group1
) |> add_overall() |> add_p() |> add_q()

as_flex_table(TableComp) |> flextable::set_table_properties(
 layout = "autofit",
 width = 0.15,
 align = "center",
 opts_word = list(
 split = TRUE
 )
 )

| Table 27: Summary of adverse event incidence separated by Group   \| **Characteristic** \| **Overall**  N = 568^1^ \| **RAS**  N = 258^1^ \| **Pre-RAS**  N = 173^1^ \| **Non-RAS**  N = 137^1^ \| **p-value**^2^ \| **q-value**^3^ \| \| --- \| --- \| --- \| --- \| --- \| --- \| --- \| \| Bleed \| 7 (1.2%) \| 3 (1.2%) \| 1 (0.6%) \| 3 (2.2%) \| 0.4 \| 0.6 \| \| Thromboembolism \| 15 (2.6%) \| 5 (1.9%) \| 6 (3.5%) \| 4 (2.9%) \| 0.6 \| 0.7 \| \| Neural \| 4 (0.7%) \| 1 (0.4%) \| 2 (1.2%) \| 1 (0.7%) \| 0.8 \| 0.9 \| \| Vascular \| 1 (0.2%) \| 1 (0.4%) \| 0 (0%) \| 0 (0%) \| >0.9 \| >0.9 \| \| Stiffness \| 9 (1.6%) \| 7 (2.7%) \| 0 (0%) \| 2 (1.5%) \| 0.061 \| 0.4 \| \| InfectionSuperficial \| 18 (3.2%) \| 10 (3.9%) \| 4 (2.3%) \| 4 (2.9%) \| 0.7 \| 0.8 \| \| InfectionDeep \| 1 (0.2%) \| 0 (0%) \| 0 (0%) \| 1 (0.7%) \| 0.2 \| 0.5 \| \| InfectionSyst \| 2 (0.4%) \| 0 (0%) \| 1 (0.6%) \| 1 (0.7%) \| 0.3 \| 0.5 \| \| PeriprostheticFracture \| 3 (0.5%) \| 0 (0%) \| 1 (0.6%) \| 2 (1.5%) \| 0.095 \| 0.4 \| \| Patellofemoral \| 3 (0.5%) \| 1 (0.4%) \| 2 (1.2%) \| 0 (0%) \| 0.5 \| 0.6 \| \| Delivery \| 4 (0.7%) \| 4 (1.6%) \| 0 (0%) \| 0 (0%) \| 0.2 \| 0.4 \| \| Readmission \| 38 (6.7%) \| 16 (6.2%) \| 8 (4.6%) \| 14 (10%) \| 0.13 \| 0.4 \| \| Reoperation \| 24 (4.2%) \| 12 (4.7%) \| 4 (2.3%) \| 8 (5.8%) \| 0.3 \| 0.5 \| \| Revision \| 2 (0.4%) \| 0 (0%) \| 0 (0%) \| 2 (1.5%) \| 0.058 \| 0.4 \| \| ^1^n (%) \| \| \| \| \| \| \| \| ^2^Fisher's exact test; Pearson's Chi-squared test \| \| \| \| \| \| \| \| ^3^False discovery rate correction for multiple testing \| \| \| \| \| \| \| |
| --- | --- | --- | --- | --- | --- | --- | --- | --- | --- | --- | --- | --- | --- | --- | --- | --- | --- | --- | --- | --- | --- | --- | --- | --- | --- | --- | --- | --- | --- | --- | --- | --- | --- | --- | --- | --- | --- | --- | --- | --- | --- | --- | --- | --- | --- | --- | --- | --- | --- | --- | --- | --- | --- | --- | --- | --- | --- | --- | --- | --- | --- | --- | --- | --- | --- | --- | --- | --- | --- | --- | --- | --- | --- | --- | --- | --- | --- | --- | --- | --- | --- | --- | --- | --- | --- | --- | --- | --- | --- | --- | --- | --- | --- | --- | --- | --- | --- | --- | --- | --- | --- | --- | --- | --- | --- | --- | --- | --- | --- | --- | --- | --- | --- | --- | --- | --- | --- | --- | --- | --- | --- | --- | --- | --- | --- | --- |

TableCov <- tbl_summary(
 MasterSheetKM |> dplyr::select(
 Group1,
 AgeAtSurgery,
 Sex,
 BMI,
 ComorbidCardiovascular,
 ComorbidSmoke,
 ComorbidDiabetes
 ),
 by = Group1,
 missing = "no"
) |> add_overall() |> add_p()

as_flex_table(TableCov) |> flextable::set_table_properties(
 layout = "autofit",
 width = 0.15,
 align = "center",
 opts_word = list(
 split = TRUE
 )
 )

| Table 28: Summary of model covariates for adverse events separated by Group   \| **Characteristic** \| **Overall**  N = 568^1^ \| **RAS**  N = 258^1^ \| **Pre-RAS**  N = 173^1^ \| **Non-RAS**  N = 137^1^ \| **p-value**^2^ \| \| --- \| --- \| --- \| --- \| --- \| --- \| \| AgeAtSurgery \| 69 (62, 76) \| 69 (64, 76) \| 68 (61, 75) \| 70 (63, 77) \| 0.2 \| \| Sex \|  \|  \|  \|  \| 0.12 \| \| Female \| 322 (57%) \| 141 (55%) \| 93 (54%) \| 88 (64%) \|  \| \| Male \| 246 (43%) \| 117 (45%) \| 80 (46%) \| 49 (36%) \|  \| \| BMI \| 32.2 (28.1, 36.8) \| 32.3 (28.5, 36.8) \| 32.0 (27.6, 37.1) \| 32.3 (28.3, 35.9) \| 0.7 \| \| ComorbidCardiovascular \| 16 (2.8%) \| 9 (3.5%) \| 1 (0.6%) \| 6 (4.4%) \| 0.067 \| \| ComorbidSmoke \| 34 (6.0%) \| 13 (5.0%) \| 16 (9.2%) \| 5 (3.6%) \| 0.082 \| \| ComorbidDiabetes \| 86 (15%) \| 43 (17%) \| 25 (14%) \| 18 (13%) \| 0.6 \| \| ^1^Median (Q1, Q3); n (%) \| \| \| \| \| \| \| ^2^Kruskal-Wallis rank sum test; Pearson's Chi-squared test; Fisher's exact test \| \| \| \| \| \| |
| --- | --- | --- | --- | --- | --- | --- | --- | --- | --- | --- | --- | --- | --- | --- | --- | --- | --- | --- | --- | --- | --- | --- | --- | --- | --- | --- | --- | --- | --- | --- | --- | --- | --- | --- | --- | --- | --- | --- | --- | --- | --- | --- | --- | --- | --- | --- | --- | --- | --- | --- | --- | --- | --- | --- | --- | --- | --- | --- | --- | --- | --- | --- | --- | --- | --- | --- |

An important assumption for Cox proportional hazards models is that the hazard function for each Group remains proportional to the other Groups over the follow up period (Kuitunen et al. 2021). The assumption was assessed using a score test calculated in the survival package.

#coxph diagnostics
# https://shariq-mohammed.github.io/files/cbsa2019/1-intro-to-survival.html#6_cox_regression


test.ph1 = survival::cox.zph(ModelCox1)
test.ph2 = survival::cox.zph(ModelCox2)
test.ph3 = survival::cox.zph(ModelCox3)
test.ph4 = survival::cox.zph(ModelCox4)


# Assuming the cox.zph tests have been run as shown

# Create a function to format the cox.zph results
format_coxzph <- function(test, model_name) {
 test$table |>
 as.data.frame() |>
 rownames_to_column("term") |>
 mutate(
 model = model_name,
 p.value = p
 ) |>
 select(model, term, p.value)
}

# Apply the function to each model's test results
ph_test1 <- format_coxzph(test.ph1, "Superficial Infection")
ph_test2 <- format_coxzph(test.ph2, "VTE")
ph_test3 <- format_coxzph(test.ph3, "Stiffness")
ph_test4 <- format_coxzph(test.ph4, "All-cause Readmission")

# Combine all test results
all_ph_tests <- bind_rows(ph_test1, ph_test2, ph_test3, ph_test4)

# Create the summary table
ph_summary_table <- all_ph_tests |>
 mutate(
 term = ifelse(term == "GLOBAL", "Global test", term)
 ) |>
 pivot_wider(
 names_from = model,
 values_from = p.value
 ) |>
 gtsummary::tbl_summary(
 by = term,
 type = everything() ~ "continuous",
 digits = everything() ~ 3,
 missing = "no",
 statistic = everything() ~ "{median}"
 ) |>
 modify_header(all_stat_cols() ~ "**{level}**") |>
 #modify_spanning_header(all_stat_cols() ~ "**Model**") |>
 modify_caption("Proportional Hazards Assumption Test (p-values)") |>
 modify_footnote(everything() ~ "Median p-value shown")

# Display the table
as_flex_table(ph_summary_table) |> flextable::set_table_properties(
 layout = "autofit",
 width = 0.15,
 align = "center",
 opts_word = list(
 split = TRUE
 )
 )

| Table 29: Summary of proportional hazards assumption test p-values for adverse events adjusted Cox models   \| **Characteristic**^1^ \| **ComorbidCardiovascular**^1^ \| **ComorbidDiabetes**^1^ \| **ComorbidSmoke**^1^ \| **Global test**^1^ \| **Group1**^1^ \| **pspline(BMI, df = 3)**^1^ \| **Sex**^1^ \| \| --- \| --- \| --- \| --- \| --- \| --- \| --- \| --- \| \| Superficial Infection \| NA \| NA \| 0.963 \| 0.634 \| 0.634 \| NA \| 0.180 \| \| VTE \| NA \| 0.482 \| 0.310 \| 0.863 \| 0.983 \| NA \| 0.643 \| \| Stiffness \| NA \| 1.000 \| NA \| 0.066 \| 0.079 \| NA \| 0.064 \| \| All-cause Readmission \| 1.000 \| NA \| 0.649 \| 0.855 \| 0.628 \| 0.855 \| 0.157 \| \| ^1^Median p-value shown \| \| \| \| \| \| \| \| |
| --- | --- | --- | --- | --- | --- | --- | --- | --- | --- | --- | --- | --- | --- | --- | --- | --- | --- | --- | --- | --- | --- | --- | --- | --- | --- | --- | --- | --- | --- | --- | --- | --- | --- | --- | --- | --- | --- | --- | --- | --- | --- | --- | --- | --- | --- | --- | --- | --- |

TableCox1 <- tbl_regression(
 ModelCox1,
 exponentiate = TRUE,
 show_single_row = c(
 "Sex",
 "ComorbidSmoke"),
 label = list(
 Sex = "Sex (Male)",
 ComorbidSmoke = "Smoking History"
 )
)

TableCox2 <- tbl_regression(
 ModelCox2,
 exponentiate = TRUE,
 show_single_row = c(
 "Sex",
 "ComorbidDiabetes",
 "ComorbidSmoke"
 ),
 label = list(
 Sex = "Sex (Male)",
 ComorbidDiabetes = "Diabetes",
 ComorbidSmoke = "Smoking History"
 )
)

TableCox3 <- tbl_regression(
 ModelCox3,
 exponentiate = TRUE,
 show_single_row = c(
 "Sex",
 "ComorbidDiabetes"
 ),
 label = list(
 Sex = "Sex (Male)",
 ComorbidDiabetes = "Diabetes"
 )
)

TableCox4 <- tbl_regression(
 ModelCox4,
 exponentiate = TRUE,
 show_single_row = c(
 "Sex",
 "ComorbidSmoke",
 "ComorbidCardiovascular"
 ),
 label = list(
 Sex = "Sex (Male)",
 #`pspline(BMI, df = 3)` = "Body Mass Index",
 ComorbidSmoke = "Smoking History",
 ComorbidCardiovascular = "Cardiovascular Disease"

 )
)

TableCox <- tbl_merge(
 list(TableCox1,
 TableCox2,
 TableCox3,
 TableCox4
 ),
 tab_spanner = c("Superficial Infection",
 "Thromboembolism",
 "Stiffness",
 "Readmission"
 )
)


as_flex_table(TableCox) |> flextable::set_table_properties(
 layout = "autofit",
 width = 0.15,
 align = "center",
 opts_word = list(
 split = TRUE
 )
 )

| Table 30: Summary of adjusted Cox proportional hazards models for surgical site infection, venous thromboembolism, stiffness and all-cause readmission   \|  \| Superficial Infection \| \| \| Thromboembolism \| \| \| Stiffness \| \| \| Readmission \| \| \| \| --- \| --- \| --- \| --- \| --- \| --- \| --- \| --- \| --- \| --- \| --- \| --- \| --- \| \| **Characteristic** \| **HR** \| **95% CI** \| **p-value** \| **HR** \| **95% CI** \| **p-value** \| **HR** \| **95% CI** \| **p-value** \| **HR** \| **95% CI** \| **p-value** \| \| Group1 \|  \|  \|  \|  \|  \|  \|  \|  \|  \|  \|  \|  \| \| RAS \| — \| — \|  \| — \| — \|  \| — \| — \|  \| — \| — \|  \| \| Pre-RAS \| 0.58 \| 0.18, 1.92 \| 0.4 \| 1.80 \| 0.54, 6.04 \| 0.3 \| 0.00 \| 0.00, Inf \| >0.9 \| 0.66 \| 0.28, 1.55 \| 0.3 \| \| Non-RAS \| 0.75 \| 0.24, 2.27 \| 0.6 \| 1.44 \| 0.37, 5.63 \| 0.6 \| 0.53 \| 0.11, 2.53 \| 0.4 \| 1.70 \| 0.83, 3.52 \| 0.15 \| \| Sex (Male) \| 1.05 \| 0.42, 2.64 \| >0.9 \| 0.64 \| 0.21, 2.00 \| 0.4 \| 2.95 \| 0.74, 11.8 \| 0.13 \| 1.81 \| 0.95, 3.46 \| 0.072 \| \| Smoking History \| 0.98 \| 0.13, 7.67 \| >0.9 \| 1.01 \| 0.12, 8.29 \| >0.9 \|  \|  \|  \| 0.55 \| 0.07, 4.03 \| 0.6 \| \| Diabetes \|  \|  \|  \| 1.49 \| 0.39, 5.66 \| 0.6 \| 0.00 \| 0.00, Inf \| >0.9 \|  \|  \|  \| \| Cardiovascular Disease \|  \|  \|  \|  \|  \|  \|  \|  \|  \| 0.00 \| 0.00, Inf \| >0.9 \| \| Abbreviations: CI = Confidence Interval, HR = Hazard Ratio \| \| \| \| \| \| \| \| \| \| \| \| \| |
| --- | --- | --- | --- | --- | --- | --- | --- | --- | --- | --- | --- | --- | --- | --- | --- | --- | --- | --- | --- | --- | --- | --- | --- | --- | --- | --- | --- | --- | --- | --- | --- | --- | --- | --- | --- | --- | --- | --- | --- | --- | --- | --- | --- | --- | --- | --- | --- | --- | --- | --- | --- | --- | --- | --- | --- | --- | --- | --- | --- | --- | --- | --- | --- | --- | --- | --- | --- | --- | --- | --- | --- | --- | --- | --- | --- | --- | --- | --- | --- | --- | --- | --- | --- | --- | --- | --- | --- | --- | --- | --- | --- | --- | --- | --- | --- | --- | --- | --- | --- | --- | --- | --- | --- | --- | --- | --- | --- | --- | --- | --- | --- | --- | --- | --- | --- | --- | --- | --- | --- | --- | --- | --- | --- | --- | --- | --- | --- | --- | --- | --- | --- | --- | --- | --- | --- | --- | --- | --- | --- | --- | --- | --- | --- |

The adjusted models revealed no significant effect of Group on any incidence of SSI, VTE, stiffness or all-cause readmission after total knee arthroplasty in the first 90 days after surgery (Table 30).

# Maybe swap out for this
# https://cran.r-project.org/web/packages/survivalAnalysis/vignettes/multivariate.html

# Tidy the model output
tidy_cox1 <- broom::tidy(ModelCox1)
tidy_cox2 <- broom::tidy(ModelCox2)
tidy_cox3 <- broom::tidy(ModelCox3)
tidy_cox4 <- broom::tidy(ModelCox4)

# Add confidence intervals
tidy_cox1 <- tidy_cox1 |>
 mutate(conf.low = estimate - 1.96 * std.error,
 conf.high = estimate + 1.96 * std.error)

tidy_cox2 <- tidy_cox2 |>
 mutate(conf.low = estimate - 1.96 * std.error,
 conf.high = estimate + 1.96 * std.error)

tidy_cox3 <- tidy_cox3 |>
 mutate(conf.low = estimate - 1.96 * std.error,
 conf.high = estimate + 1.96 * std.error)

tidy_cox4 <- tidy_cox4 |>
 mutate(conf.low = estimate - 1.96 * std.error,
 conf.high = estimate + 1.96 * std.error)

# Plot using ggplot2
FigureCox1 <- ggplot(tidy_cox1, aes(x = term, y = estimate, ymin = conf.low, ymax = conf.high)) +
 geom_pointrange() +
 coord_flip() + # Flip the coordinates for better readability
 labs(
 title = "Superficial Infection",
 x = "Terms",
 y = "Coefficient Estimate"
 ) +
 theme_ggdist()

FigureCox2 <- ggplot(tidy_cox2, aes(x = term, y = estimate, ymin = conf.low, ymax = conf.high)) +
 geom_pointrange() +
 coord_flip() + # Flip the coordinates for better readability
 labs(
 title = "Thromboembolism",
 x = "Terms",
 y = "Coefficient Estimate"
 ) +
 theme_ggdist()

FigureCox3 <- ggplot(tidy_cox3, aes(x = term, y = estimate, ymin = conf.low, ymax = conf.high)) +
 geom_pointrange() +
 coord_flip() + # Flip the coordinates for better readability
 labs(
 title = "Stiffness",
 x = "Terms",
 y = "Coefficient Estimate"
 ) +
 theme_ggdist()

FigureCox4 <- ggplot(tidy_cox4, aes(x = term, y = estimate, ymin = conf.low, ymax = conf.high)) +
 geom_pointrange() +
 coord_flip() + # Flip the coordinates for better readability
 labs(
 title = "Readmission",
 x = "Terms",
 y = "Coefficient Estimate"
 ) +
 theme_ggdist()

patchwork::wrap_plots(FigureCox1,FigureCox2)

| 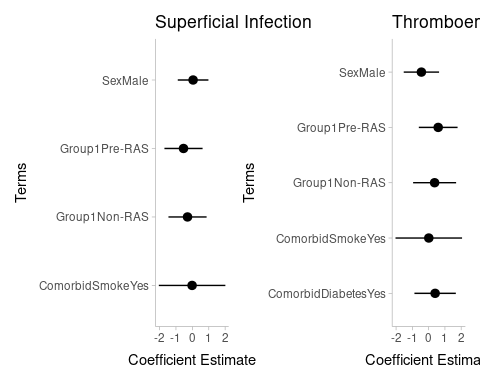  Figure 22: Summary of adjusted cox proportional hazards models for SSI (Left) and VTE (Right) |
| --- |

Visualisation of the coefficients and confidence intervals for fixed effects (superficial infection, thromboembolism) showed overlap with zero and substantial variability (Figure 22).

ptemp <- termplot(ModelCox4, se = TRUE, plot = FALSE)
attributes(ptemp)

$constant
[1] -2.200582

$names
[1] "Group1" "Sex" "ComorbidSmoke"
[4] "ComorbidCardiovascular" "BMI"

# Extract the term data
term <- ptemp$BMI

# Calculate the center value by mean
center <- mean(term$x)

# Calculate y values with confidence intervals
ytemp <- term$y + outer(term$se, c(0, -1.96, 1.96), '*')

# Create a data frame for ggplot2
df <- data.frame(
 BMI = rep(term$x, 3),
 RelativeRate = exp(c(ytemp[,1], ytemp[,2], ytemp[,3])),
 Type = factor(rep(c("Estimate", "Lower", "Upper"), each = nrow(ytemp)))
)

# Plot using ggplot2
FigureCox1 <- ggplot(df, aes(x = BMI, y = RelativeRate, linetype = Type)) +
 geom_line(color = "black") +
 scale_y_log10() +
 labs(
 x = "BMI",
 y = "Relative readmission rate",
 linetype = "Type"
 ) +
 theme_minimal()

knitr::knit_print(FigureCox1)

| 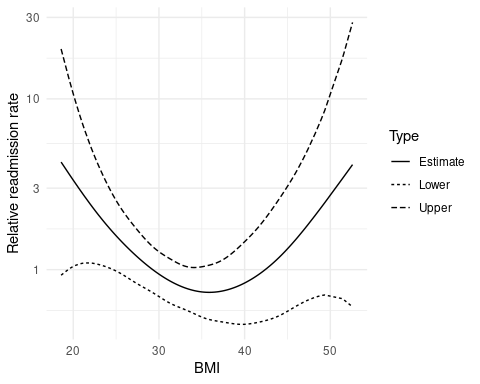  Figure 23: Relationship between penalised spline of body mass index on all-cause readmission risk. |
| --- |

ModelCox1a <- survival::coxph(Surv(DurationInfectSuper,InfectionSuperficialBin) ~ Group1 + Sex + ComorbidSmoke,
 data = MasterSheetKM_Clean,
 x = TRUE
)


ModelCox2a <- survival::coxph(Surv(DurationThrombo,ThromboembolismBin) ~ Group1 + Sex + ComorbidSmoke + ComorbidDiabetes,
 data = MasterSheetKM_Clean,
 x = TRUE
)

ModelCox3a <- survival::coxph(Surv(DurationStiff,StiffnessBin) ~ Group1 + Sex + ComorbidDiabetes,
 data = MasterSheetKM_Clean,
 x = TRUE
)

ModelCox4a <- survival::coxph(Surv(DurationReadmit,ReadmissionBin) ~ Group1 + Sex + ComorbidSmoke + ComorbidDiabetes + ComorbidCardiovascular,
 data = MasterSheetKM_Clean,
 x = TRUE
)

AdjustCurve1 <- adjustedCurves::adjustedsurv(data= MasterSheetKM_Clean,
 variable="Group1",
 ev_time="DurationInfectSuper",
 event="InfectionSuperficialBin",
 method="direct",
 outcome_model= ModelCox1a,
 conf_int=TRUE,
 bootstrap = TRUE,
 n_boot = 100)

AdjustCurve2 <- adjustedCurves::adjustedsurv(data= MasterSheetKM_Clean,
 variable="Group1",
 ev_time="DurationThrombo",
 event="ThromboembolismBin",
 method="direct",
 outcome_model= ModelCox2a,
 conf_int=TRUE,
 bootstrap = TRUE,
 n_boot = 100)

AdjustCurve3 <- adjustedCurves::adjustedsurv(data= MasterSheetKM_Clean,
 variable="Group1",
 ev_time="DurationStiff",
 event="StiffnessBin",
 method="direct",
 outcome_model= ModelCox3a,
 conf_int=TRUE,
 bootstrap = TRUE,
 n_boot = 100)

AdjustCurve4 <- adjustedCurves::adjustedsurv(data= MasterSheetKM_Clean,
 variable="Group1",
 ev_time="DurationReadmit",
 event="ReadmissionBin",
 method="direct",
 outcome_model= ModelCox4a,
 conf_int=TRUE,
 bootstrap = TRUE,
 n_boot = 100)

AdjustCurveFig1 <- plot(
 AdjustCurve1,
 conf_int = FALSE,
 gg_theme=ggdist::theme_ggdist(),
 # risk_table = TRUE,
 # risk_table_type = "n_events",
 # risk_table_stratify = TRUE,
 # risk_table_height = 0.35
 )

AdjustCurveFig2 <- plot(
 AdjustCurve2,
 conf_int = FALSE,
 gg_theme=ggdist::theme_ggdist(),
 # risk_table = TRUE,
 # risk_table_type = "n_events",
 # risk_table_stratify = TRUE,
 # risk_table_height = 0.35
 )

AdjustCurveFig1 / AdjustCurveFig2

| 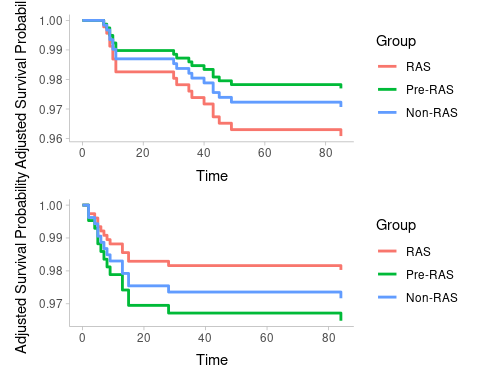  Figure 24: Adjusted survival curves for surgical site infection (top) and venous thromboembolism (bottom) by Group |
| --- |

AdjustCurveFig3 <- plot(
 AdjustCurve3,
 conf_int = FALSE,
 gg_theme = ggdist::theme_ggdist(),
 # risk_table = TRUE,
 # risk_table_type = "n_events",
 # risk_table_stratify = TRUE,
 # risk_table_height = 0.35
 )

AdjustCurveFig4 <- plot(
 AdjustCurve4,
 conf_int = FALSE,
 gg_theme = ggdist::theme_ggdist(),
 # risk_table = TRUE,
 # risk_table_type = "n_events",
 # risk_table_stratify = TRUE,
 # risk_table_height = 0.35
 )

AdjustCurveFig3 / AdjustCurveFig4

| 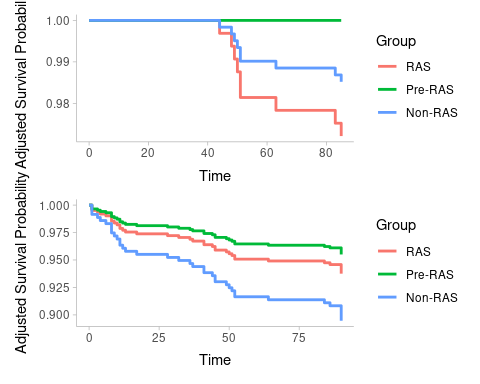  Figure 25: Adjusted survival curves for stiffness (top) and all-cause readmission (bottom) by Group |
| --- |

## 6.5 STROBE [17] Other Analyses:

*Critically analyze the presentation and interpretation of subgroups, interactions or the description and evaluation of sensitivity analyses.*

# Step 1: Prepare the data
MasterSheetKM_Clean <- MasterSheetKM_Clean |>
 mutate(Group1Sex = interaction(Group1, Sex))

# Step 2: Fit the Kaplan-Meier model
km_fit1 <- survfit(Surv(DurationInfectSuper, InfectionSuperficialBin) ~ Group1Sex,
 data = MasterSheetKM_Clean)

# Step 3: Create the table
TableKM1 <- km_fit1 |>
 tbl_survfit(
 times = c(14, 90),
 label_header = "**Days {time}**"
 ) |>
 add_p() |>
 add_n() |>
 add_nevent()


# Step 1: Prepare the data
MasterSheetKM_Clean <- MasterSheetKM_Clean |>
 mutate(Group1Sex = interaction(Group1, Sex))

# Step 2: Fit the Kaplan-Meier model
km_fit2 <- survfit(Surv(DurationInfectSuper, InfectionSuperficialBin) ~ Group1,
 data = MasterSheetKM_Clean |> filter(Sex == "Male"))

# Step 3: Create the table
TableKM2 <- km_fit2 |>
 tbl_survfit(
 times = c(14, 90),
 label_header = "**Days {time}**"
 ) |>
 add_p() |>
 add_n() |>
 add_nevent()


TableKMComb <- tbl_stack(
list(TableKM1,TableKM2),
group_header = c("Group-Sex Interaction","Male Subset")
)

as_flex_table(TableKMComb) |> flextable::set_table_properties(
 layout = "autofit",
 width = 0.15,
 align = "center",
 opts_word = list(
 split = TRUE
 )
 )

| Table 31: Summary of survival from superficial infection up to 90 days after total knee arthroplasty by group*sex interaction and for males only compared between Group   \| **Group** \| **Characteristic** \| **N** \| **Event N** \| **Days 14** \| **Days 90** \| **p-value**^1^ \| \| --- \| --- \| --- \| --- \| --- \| --- \| --- \| \| Group-Sex Interaction \| Group1Sex \| 565 \| 18 \|  \|  \| 0.2 \| \|  \| RAS.Female \|  \|  \| 99% (97%, 100%) \| 98% (95%, 100%) \|  \| \|  \| Pre-RAS.Female \|  \|  \| 99% (97%, 100%) \| 97% (93%, 100%) \|  \| \|  \| Non-RAS.Female \|  \|  \| 97% (93%, 100%) \| 95% (91%, 100%) \|  \| \|  \| RAS.Male \|  \|  \| 98% (96%, 100%) \| 94% (90%, 98%) \|  \| \|  \| Pre-RAS.Male \|  \|  \| 100% (100%, 100%) \| 99% (96%, 100%) \|  \| \|  \| Non-RAS.Male \|  \|  \| 100% (100%, 100%) \| 100% (100%, 100%) \|  \| \| Male Subset \| Group1 \| 244 \| 8 \|  \|  \| 0.062 \| \|  \| RAS \|  \|  \| 98% (96%, 100%) \| 94% (90%, 98%) \|  \| \|  \| Pre-RAS \|  \|  \| 100% (100%, 100%) \| 99% (96%, 100%) \|  \| \|  \| Non-RAS \|  \|  \| 100% (100%, 100%) \| 100% (100%, 100%) \|  \| \| ^1^Log-rank test \| \| \| \| \| \| \| |
| --- | --- | --- | --- | --- | --- | --- | --- | --- | --- | --- | --- | --- | --- | --- | --- | --- | --- | --- | --- | --- | --- | --- | --- | --- | --- | --- | --- | --- | --- | --- | --- | --- | --- | --- | --- | --- | --- | --- | --- | --- | --- | --- | --- | --- | --- | --- | --- | --- | --- | --- | --- | --- | --- | --- | --- | --- | --- | --- | --- | --- | --- | --- | --- | --- | --- | --- | --- | --- | --- | --- | --- | --- | --- | --- | --- | --- | --- | --- | --- | --- | --- | --- | --- | --- | --- | --- | --- | --- | --- | --- | --- |

The survival rate at 90 day follow up for males undergoing RAS was 94% [95% CI 90% - 98%], but this did not reach significance when compared to the other groups (p=0.062).

### 6.5.1 Model sensitivity - Procedure duration

model_inverse <- lme4::glmer(ProcedureDuration ~ Group1 + BilateralStatus + (1|PatientID2),
 family = Gamma(link = "log"),
 data = Duration_Analysis)
model_log <- lme4::glmer(ProcedureDuration ~ Group1 + BilateralStatus + (1|PatientID2),
 family = Gamma(link = "inverse"),
 data = Duration_Analysis)
model_identity <- lme4::glmer(ProcedureDuration ~ Group1 + BilateralStatus + (1|PatientID2),
 family = Gamma(link = "identity"),
 data = Duration_Analysis)
model_sqrt <- lme4::glmer(ProcedureDuration ~ Group1 + BilateralStatus + (1|PatientID2),
 family = Gamma(link = "sqrt"),
 data = Duration_Analysis)

# Calculate AIC and BIC values for each model
AIC_values <- AIC(model_inverse, model_log, model_identity, model_sqrt)
BIC_values <- BIC(model_inverse, model_log, model_identity, model_sqrt)

# Create a data frame with the AIC and BIC values
results <- data.frame(
 Model = c("Inverse Link", "Log Link", "Identity Link", "Square Root Link"),
 AIC = AIC_values[,2],
 BIC = BIC_values[,2]
)

# Convert the data frame to a gt table
results_table <- results |>
 gt() |>
 tab_header(
 title = "Model Comparison: AIC and BIC"
 ) |>
 cols_label(
 Model = "Model",
 AIC = "AIC",
 BIC = "BIC"
 )

knitr::knit_print(results_table) #gt table, cant be converted to flextable

| Table 32: Summary of Akaike information criterion and Bayesian information criterion values for four variations of the gamma link function for procedure duration modelling  Table 2: Model Comparison: AIC and BIC   \| Model \| AIC \| BIC \| \| --- \| --- \| --- \| \| Inverse Link \| 4689.515 \| 4715.547 \| \| Log Link \| 4666.676 \| 4692.707 \| \| Identity Link \| 4854.735 \| 4880.767 \| \| Square Root Link \| 4755.208 \| 4781.239 \| |
| --- | --- | --- | --- | --- | --- | --- | --- | --- | --- | --- | --- | --- | --- | --- | --- |

Model predictions displayed consistent patterns, regardless of the link function used.

#marginaleffects package

DurationPredictInv <- marginaleffects::predictions(model_inverse, re.form = NA)
DurationPredictLog <- marginaleffects::predictions(model_log, re.form = NA)
DurationPredictIdent <- marginaleffects::predictions(model_identity, re.form = NA)
DurationPredictSqrt <- marginaleffects::predictions(model_sqrt, re.form = NA)

DurationFigInv <- ggplot(data = DurationPredictInv, mapping = aes(y = Group1, x = ProcedureDuration)) +
 stat_slab(aes(thickness = after_stat(pdf*n)), scale = 0.7) +
 stat_dotsinterval(side = "bottom", scale = 0.7, slab_linewidth = NA) +
 #scale_fill_brewer(palette = "Set2") +
 ggtitle('Inverse')

DurationFigLog <- ggplot(data = DurationPredictLog, mapping = aes(y = Group1, x = ProcedureDuration)) +
 stat_slab(aes(thickness = after_stat(pdf*n)), scale = 0.7) +
 stat_dotsinterval(side = "bottom", scale = 0.7, slab_linewidth = NA) +
 #scale_fill_brewer(palette = "Set2") +
 ggtitle('Log')

DurationFigIdent <- ggplot(data = DurationPredictIdent, mapping = aes(y = Group1, x = ProcedureDuration)) +
 stat_slab(aes(thickness = after_stat(pdf*n)), scale = 0.7) +
 stat_dotsinterval(side = "bottom", scale = 0.7, slab_linewidth = NA) +
 #scale_fill_brewer(palette = "Set2") +
 ggtitle('Identity')

DurationFigSqrt <- ggplot(data = DurationPredictSqrt, mapping = aes(y = Group1, x = ProcedureDuration)) +
 stat_slab(aes(thickness = after_stat(pdf*n)), scale = 0.7) +
 stat_dotsinterval(side = "bottom", scale = 0.7, slab_linewidth = NA) +
 #scale_fill_brewer(palette = "Set2") +
 ggtitle('Square Root')

patchwork::wrap_plots(DurationFigInv,DurationFigLog,DurationFigIdent,DurationFigSqrt)

| 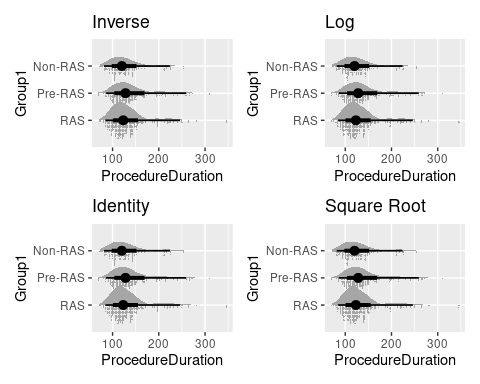  Figure 26: Model predicted procedure duration with different link functions within the gamma family of generalised linear models for procedure duration |
| --- |

# Function to create a ggplot of residuals vs fitted
plot_residuals <- function(model, title) {
 df <- data.frame(
 fitted = fitted(model),
 residuals = residuals(model, type = "pearson")
 )
 ggplot(df, aes(x = fitted, y = residuals)) +
 geom_point() +
 geom_hline(yintercept = 0, linetype = "dashed", color = "red") +
 ggtitle(title) +
 xlab("Fitted values") +
 ylab("Pearson residuals")
}

# Create plots for each model
p1 <- plot_residuals(model_inverse, "Inverse Link")
p2 <- plot_residuals(model_log, "Log Link")
p3 <- plot_residuals(model_identity, "Identity Link")
p4 <- plot_residuals(model_sqrt, "Square Root Link")


patchwork::wrap_plots(p1,p2,p3,p4)

| 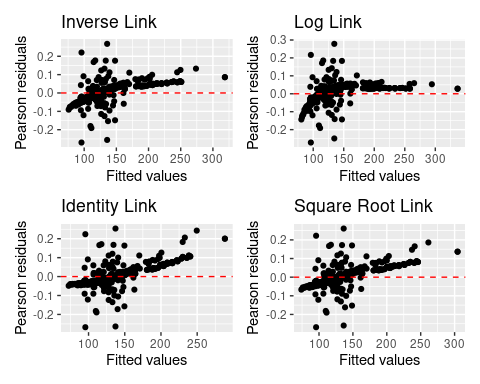  Figure 27: Summary of residuals across variations of link function within the gamma family of generalised linear models for procedure duration |
| --- |

Four link functions (log, inverse, identity and square root) within the gamma family were compared to assess the robustness of the primary model specification. Model fit was evaluated using the Akaike information criterion (AIC) and Bayesion information criterion (BIC) with the log function producing the lowest values (Table 32). The marginal predicted distributions, as well as the residuals, across the three study groups were visually consistent across all link functions ( Figure 26 and Figure 27). These results indicated that the substantive finding that procedure duration did not differ between groups, was not sensitive to the link function choice.

### 6.5.2 Model sensitivity - Surgeon

Based on reviewer feedback 24-Jun-2026, sensitivity analyses were conducted on procedure duration and adverse events to test the potential influence of surgeon variation on the group comparisons. Rather than subset the dataset as suggested, the mixed effects models were extended by including surgeon as an additional random effect in both the linear model (procedure duration) and the cox proportional hazards survival model (readmission incidence).

#### Procedure Duration

The primary procedure duration model, which accounted for non-independence between bilateral cases via a patient-level random intercept, did not separately account for surgeon-level variation. Surgeon-level clustering in outcomes studies of orthopaedic surgery is chronically under-reported in the literature (Oltean and Gagnier 2015) and is usually ignored in the studies such as the one described here. Nevertheless, it is a recognised source of unmeasured confounding in surgical outcomes research (Cook et al. 2012). Intracluster correlation coefficients (ICCs) indicate that outcomes such as procedure duration can vary between surgeons independent of treatment allocation

To assess whether Surgeon influenced the present results, a sensitivity analysis was performed by extending the primary model of procedure duration with an additional surgeon-level random intercept. A random intercept is the appropriate design choice as it enables an assessment of surgeon-level confounding within the model without sacrificing degrees of freedom. The fixed effects for 7 surgeons would consume the cases-per-surgeon imbalance within the model and lead to difficulties with convergence and inflating uncertainty within the estimates.

Surgeon as a random effect was specified as crossed rather than nested with the existing patient-level term, since individual surgeons’ caseloads span multiple groups rather than being hierarchically subsumed within them (Baayen et al. 2008) . The relative contribution of surgeon-level variation was quantified using the ICC, defined as the proportion of total outcome variance attributable to a given grouping factor (Shrout and Fleiss 1979), allowing direct comparison of surgeon-, patient-, and residual-level contributions to variance in procedure duration. Given the small number of surgeon levels (n = 7) contributing to this cohort, variance component estimates at the surgeon level are subject to reduced precision (Maas and Hox 2005), therefore this model is presented as a sensitivity check rather than a replacement.

Surgeon was added to the analysis subset for procedure duration.

# Add Surgeon to the Duration_Analysis subset
Duration_Analysis_Surgeon <- MasterSheet6 |> dplyr::select(
 PatientID2,
 ProcedureDuration,
 Group1,
 BilateralStatus,
 Surgeon
) |>
 mutate(
 BilateralStatus = forcats::fct(BilateralStatus,
 levels = c("Unilateral", "Bilateral"))
 )

Surgeon was added as a crossed random effect to the existing model and fit using the bobyqa optimiser [citation] implemented in lme4, after the default optimiser failed to converge, with the maximum number of function evaluations increased to 200 000 to enable convergence.

# Fit crossed random effects model with Surgeon and PatientID2
Duration_model_surgeon <- lme4::glmer(
 ProcedureDuration ~ Group1 + BilateralStatus + (1|PatientID2) + (1|Surgeon),
 family = Gamma(link = "log"),
 data = Duration_Analysis_Surgeon,
 control = lme4::glmerControl(optimizer = "bobyqa", optCtrl = list(maxfun = 2e5))
)

# AIC and BIC comparison between primary model and surgeon random effect model
AIC_surgeon <- AIC(Duration_model, Duration_model_surgeon)
BIC_surgeon <- BIC(Duration_model, Duration_model_surgeon)

surgeon_comparison <- data.frame(
 Model = c("Primary (PatientID2 only)", "Surgeon random effect (PatientID2 + Surgeon)"),
 AIC = AIC_surgeon[, 2],
 BIC = BIC_surgeon[, 2]
)

surgeon_comparison_table <- surgeon_comparison |>
 gt() |>
 cols_label(
 Model = "Model",
 AIC = "AIC",
 BIC = "BIC"
 )

knitr::knit_print(surgeon_comparison_table)

| Table 33: Model comparison: Primary vs surgeon random effect   \| Model \| AIC \| BIC \| \| --- \| --- \| --- \| \| Primary (PatientID2 only) \| 4689.515 \| 4715.547 \| \| Surgeon random effect (PatientID2 + Surgeon) \| 4653.427 \| 4683.797 \| |
| --- | --- | --- | --- | --- | --- | --- | --- | --- | --- |

The comparison between models for AIC and BIC revealed a marginal improvement in fits when Surgeon was added as an additional factor ( Table 33 )

# Regression table for surgeon random effect model
TableDuration_Surgeon <- tbl_regression(
 Duration_model_surgeon,
 show_single_row = "BilateralStatus",
 label = list(BilateralStatus ~ "Bilateral"),
 estimate_fun = function(x) style_number(x, digits = 2),
 pvalue_fun = function(x) style_pvalue(x, digits = 3)
)

TableDurMerge <- gtsummary::tbl_merge(list(
 TableDuration,
 TableDuration_Surgeon),
 tab_spanner = c(
 "Primary model",
 "Expanded (Surgeon)"
 )
)

as_flex_table(TableDurMerge) |>
 flextable::set_table_properties(
 layout = "autofit",
 width = 0.15,
 align = "center",
 opts_word = list(split = TRUE)
 )

| Table 34: Summary of surgeon random effect model: Primary model vs expanded sensitivity model   \|  \| Primary model \| \| \| Expanded (Surgeon) \| \| \| \| --- \| --- \| --- \| --- \| --- \| --- \| --- \| \| **Characteristic** \| **Beta** \| **95% CI** \| **p-value** \| **Beta** \| **95% CI** \| **p-value** \| \| Group1 \|  \|  \|  \|  \|  \|  \| \| RAS \| — \| — \|  \| — \| — \|  \| \| Pre-RAS \| 0.05 \| 0.02, 0.09 \| 0.004 \| 0.03 \| -0.01, 0.06 \| 0.140 \| \| Non-RAS \| -0.03 \| -0.08, 0.03 \| 0.319 \| 0.10 \| 0.03, 0.18 \| 0.006 \| \| Bilateral \| -0.02 \| -0.07, 0.03 \| 0.469 \| -0.01 \| -0.06, 0.04 \| 0.693 \| \| Abbreviation: CI = Confidence Interval \| \| \| \| \| \| \| |
| --- | --- | --- | --- | --- | --- | --- | --- | --- | --- | --- | --- | --- | --- | --- | --- | --- | --- | --- | --- | --- | --- | --- | --- | --- | --- | --- | --- | --- | --- | --- | --- | --- | --- | --- | --- | --- | --- | --- | --- | --- | --- | --- | --- | --- | --- | --- | --- | --- | --- | --- | --- | --- | --- | --- | --- | --- |

The crossed random effects model with Surgeon added as a random intercept shows a noticeable effect on the magnitude, direction and significance of the coefficients compared to the primary model (Table 34). However, the expanded model relies on within-surgeon contrasts with some balance to estimate the fixed effect of Group. As shown by the counts (Table 19), this assumption is well supported for the Pre-RAS vs RAS contrast, given that Surgeons A, B and C contributed substantively to both groups. However, within-surgeon identification for the Non-RAS contrast is comparatively weak. Surgeon D performed Non-RAS cases exclusively, while the RAS-predominant surgeons (E,F) contributed minimal Non-RAS case volume. Only Surgeon G contributed substantially to both groups, making the within-surgeon Non-RAS estimate disproportionately informed by this Surgeon’s dataset, supplemented by partial pooling across the random-effects distribution for surgeon with little or no direct contrast. The Non-RAS coefficient from the expanded model should be interpreted with caution, as it reflects a combination of within-surgeon adjustment and model-based shrinkage rather than uniformly identified contrast across the surgeon cohort.

# Post-hoc pairwise comparisons
DurationPost_Surgeon <- avg_comparisons(
 Duration_model_surgeon,
 variables = list(Group1 = "pairwise"),
 type = "response",
 re.form = NA
) |>
 gt::gt() |>
 fmt_number(decimals = 2)

knitr::knit_print(DurationPost_Surgeon)

| Table 35: Post-hoc comparisons between groups with surgeon random effect model   \| term \| contrast \| estimate \| std.error \| statistic \| p.value \| s.value \| conf.low \| conf.high \| \| --- \| --- \| --- \| --- \| --- \| --- \| --- \| --- \| --- \| \| Group1 \| Non-RAS - Pre-RAS \| 9.16 \| 4.96 \| 1.85 \| 0.06 \| 3.95 \| -0.55 \| 18.88 \| \| Group1 \| Non-RAS - RAS \| 12.28 \| 4.55 \| 2.70 \| 0.01 \| 7.16 \| 3.36 \| 21.20 \| \| Group1 \| Pre-RAS - RAS \| 3.11 \| 2.13 \| 1.46 \| 0.14 \| 2.79 \| -1.07 \| 7.30 \| |
| --- | --- | --- | --- | --- | --- | --- | --- | --- | --- | --- | --- | --- | --- | --- | --- | --- | --- | --- | --- | --- | --- | --- | --- | --- | --- | --- | --- | --- | --- | --- | --- | --- | --- | --- | --- | --- |

The pairwise contrasts for Group also show differences to the primary model (illustrated in Table 25) for the reasons stated above.

# Model-predicted marginal means by Group
TableDurationSum_Surgeon <- avg_predictions(
 Duration_model_surgeon,
 by = "Group1",
 re.form = NA,
 conf_level = 0.95,
 type = "response"
) |>
 mutate(
 standarddev = case_when(
 Group1 == "Pre-RAS" ~ std.error * (sqrt(nrow(Duration_Analysis_Surgeon |> filter(Group1 == "Pre-RAS")))),
 Group1 == "Non-RAS" ~ std.error * (sqrt(nrow(Duration_Analysis_Surgeon |> filter(Group1 == "Non-RAS")))),
 Group1 == "RAS" ~ std.error * (sqrt(nrow(Duration_Analysis_Surgeon |> filter(Group1 == "RAS"))))
 )
 )

# Model predicted distribution by Group
DurationPredict_Surgeon <- marginaleffects::predictions(
 Duration_model_surgeon,
 re.form = NA,
 type = "response",
 conf_level = 0.95
)

DurationFig_Surgeon <- ggplot(
 data = DurationPredict_Surgeon,
 mapping = aes(y = Group1, x = ProcedureDuration)
) +
 stat_slab(aes(thickness = after_stat(pdf * n)), scale = 0.7) +
 stat_dotsinterval(side = "bottom", scale = 0.7, slab_linewidth = NA) +
 ggtitle("Model Predicted Procedure Duration by Group (Surgeon Random Effect)")

knitr::knit_print(DurationFig_Surgeon)


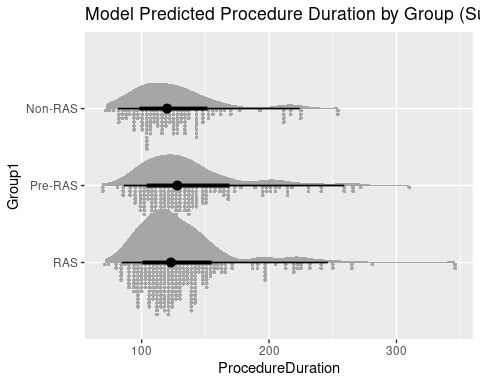


The model-predicted distributions from the expanded model showed minimal differences in shape to the primary model results.

The variance was decomposed and attributed to each random factor by calculating the intracluster correlation coefficient. The variance and proportional variances were calculated.

# Extract variance components from the crossed random effects model
vc <- lme4::VarCorr(Duration_model_surgeon)

var_surgeon <- as.numeric(vc$Surgeon)
var_patient <- as.numeric(vc$PatientID2)
var_residual <- attr(vc, "sc")^2

var_total <- var_surgeon + var_patient + var_residual

icc_surgeon <- var_surgeon / var_total
icc_patient <- var_patient / var_total
icc_resid <- var_residual / var_total

# Variance decomposition table
var_decomp <- data.frame(
 Source = c("Surgeon", "Patient (bilateral)", "Residual"),
 Variance = c(var_surgeon, var_patient, var_residual),
 ICC = c(icc_surgeon, icc_patient, icc_resid)
)

var_decomp_table <- var_decomp |>
 gt() |>
 tab_header(
 title = "Variance decomposition: crossed random effects model"
 ) |>
 cols_label(
 Source = "Source",
 Variance = "Variance",
 ICC = "ICC (proportion of total variance)"
 ) |>
 fmt_number(
 columns = c(Variance, ICC),
 decimals = 4
 )

knitr::knit_print(var_decomp_table)

| Table 36: Variance decomposition of surgeon random effect model  Table 3: Variance decomposition: crossed random effects model   \| Source \| Variance \| ICC (proportion of total variance) \| \| --- \| --- \| --- \| \| Surgeon \| 0.2247 \| 0.0396 \| \| Patient (bilateral) \| 5.4409 \| 0.9588 \| \| Residual \| 0.0092 \| 0.0016 \| |
| --- | --- | --- | --- | --- | --- | --- | --- | --- | --- | --- | --- | --- |

Surgeon as a factor in the expanded model is attributed to 4.0% of the total variance within the model (Table 36).

# Variance decomposition bar chart
FigVarDecomp <- var_decomp |>
 mutate(Source = forcats::fct(Source,
 levels = c("Residual",
 "Patient (bilateral)",
 "Surgeon"))) |>
 ggplot(aes(x = ICC, y = "Procedure Duration", fill = Source)) +
 geom_col(width = 0.4) +
 scale_x_continuous(labels = scales::percent_format(accuracy = 1),
 limits = c(0, 1)) +
 scale_fill_brewer(palette = "Set2") +
 labs(
 x = "Proportion of total variance",
 y = NULL,
 fill = "Variance source",
 title =
 ) +
 theme_minimal()

knitr::knit_print(FigVarDecomp)

| 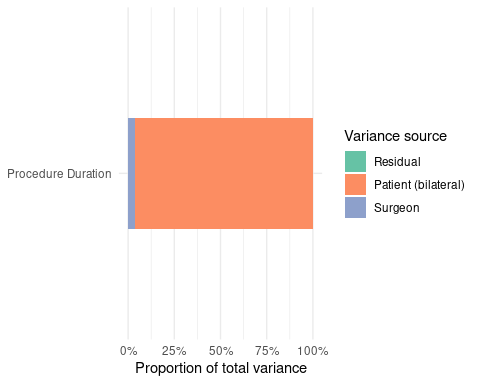  Figure 28: Variance decomposition by random effect level in sensitivity analysis |
| --- |

A sensitivity analysis was conducted to assess whether unaccounted surgeon-level variation influenced the primary finding for procedure duration. A crossed random effects structure was specified, adding a surgeon-level random intercept (1|Surgeon) to the primary patient-level random intercept (1|PatientID2). With seven surgeon levels, the surgeon variance component may be presented with degraded reliability and should be interpreted with caution. This model is presented as a sensitivity check rather than a preferred specification. Variance decomposition via intraclass correlation indicated that the surgeon random effect accounted for 4.0% of total variance in procedure duration, compared with 95.9% attributable to patient-level clustering (bilateral cases) and 0.2% residual variance (figure and table above). Model fit indices (Table 34) and pairwise group contrasts ( Table 36 ) were inspected for consistency with the primary model. Concordance between the two models in the direction, magnitude, and statistical significance of Group contrasts, in combination with the surgeon ICC, informs whether surgeon-level clustering represents a material source of confounding in the primary procedure duration analysis.

#### Readmission

All-cause readmission was the only adverse event outcome with sufficient events (n = 38) to support a surgeon-level sensitivity analysis; superficial infection (18 events), thromboembolism (15 events), and stiffness (9 events, with zero events in at least one Group) were excluded as too sparse to yield an interpretable frailty variance estimate. To assess whether unmeasured surgeon-level clustering confounded the primary readmission model, a shared gamma frailty term for Surgeon was added directly to the existing Cox specification using frailty() in the survival package , mirroring the crossed random-effects sensitivity analysis already conducted for procedure duration. The frailty term treats Surgeon as a cluster-level multiplicative random effect on the hazard, with the variance parameter (θ) estimated by penalized partial likelihood (Therneau and Grambsch 2000). With only 7 surgeon clusters and an average of 5.4 events per cluster, θ was expected to be weakly identified; this was assessed directly via the flatness of the profile likelihood across the iterative search for θ, in addition to standard model comparison (AIC, BIC) and a χ² test for the frailty term.

# ============================================================
# Surgeon-level shared frailty
# (All-cause readmission only; see justification in text)
# ============================================================

# --- 0. Rebuild mastertable with Surgeon included

MasterSensRead <- left_join(
 MasterSheetKM_Clean,
 MasterSheet6 |> dplyr::select(
 TempIDPart,
 Surgeon
 ),
 by = "TempIDPart"
)


# --- 1. Missingness check on variables entering the model ---
Readmit_Frailty <- MasterSensRead |>
 dplyr::select(
 DurationReadmit, ReadmissionBin, Group1, Sex,
 ComorbidSmoke, ComorbidCardiovascular, BMI, Surgeon,PatientID2
 ) |>
 dplyr::mutate(Surgeon = factor(Surgeon)) |>
 as.data.frame()

# naniar::miss_var_summary(Readmit_Frailty)
# naniar::vis_miss(Readmit_Frailty)

# --- 2. Confirm event sparsity across Surgeon clusters ---
Readmit_Surgeon_Events <- Readmit_Frailty |>
 dplyr::count(Surgeon, ReadmissionBin) |>
 tidyr::pivot_wider(
 names_from = ReadmissionBin,
 values_from = n,
 values_fill = 0,
 names_prefix = "Readmit_"
 ) |>
 dplyr::mutate(N = Readmit_0 + Readmit_1) |>
 dplyr::rename(Events = Readmit_1) |>
 dplyr::select(Surgeon, N, Events)

epoxy::epoxy(
 "Surgeon clusters: {nrow(Readmit_Surgeon_Events)}; ",
 "total readmission events: {sum(Readmit_Surgeon_Events$Events)}; ",
 "mean events per cluster: {round(mean(Readmit_Surgeon_Events$Events), 2)}"
)

Surgeon clusters: 7; total readmission events: 38; mean events per cluster: 5.43

# --- 2b. Surgeon x Group1 cross-tabulation ---
# Checks whether Surgeon and Group1 are themselves confounded by design
# (e.g. Non-RAS drawn from a narrow, largely non-overlapping subset of
# surgeons), which would help explain why the Non-RAS HR shifted upward
# under the frailty adjustment rather than toward the null.
Readmit_Surgeon_Group_N <- Readmit_Frailty |>
 dplyr::count(Surgeon, Group1) |>
 tidyr::pivot_wider(names_from = Group1, values_from = n, values_fill = 0)

Readmit_Surgeon_Group_Events <- Readmit_Frailty |>
 dplyr::filter(ReadmissionBin == 1) |>
 dplyr::count(Surgeon, Group1) |>
 tidyr::pivot_wider(names_from = Group1, values_from = n, values_fill = 0)

# Readmit_Surgeon_Group_N |>
# gt::gt() |>
# gt::tab_header(title = "Surgeon \u00d7 Group1: Case Counts (Readmission Analysis Subset)") |>
# knitr::knit_print()

Readmit_Surgeon_Group_Events |>
 gt::gt() |>
 knitr::knit_print()

| Table 37: Surgeon by Group: Readmission event counts   \| Surgeon \| Pre-RAS \| RAS \| Non-RAS \| \| --- \| --- \| --- \| --- \| \| A \| 3 \| 0 \| 0 \| \| B \| 0 \| 2 \| 0 \| \| C \| 5 \| 11 \| 0 \| \| D \| 0 \| 0 \| 4 \| \| E \| 0 \| 2 \| 1 \| \| F \| 0 \| 1 \| 0 \| \| G \| 0 \| 0 \| 9 \| |
| --- | --- | --- | --- | --- | --- | --- | --- | --- | --- | --- | --- | --- | --- | --- | --- | --- | --- | --- | --- | --- | --- | --- | --- | --- | --- | --- | --- | --- | --- | --- | --- | --- |

Cross tabulation of the readmission events ( Table 37) revealed non-overlapping surgeons associated with the majority of readmission events within the cohort. Therefore the frailty variance estimator may become less stable, leading to shiftis in the direction and magnitude of hazard ratio.

# --- 3. Primary model (existing, Section 11 - refit for direct comparison) ---
ModelCox4 <- survival::coxph(
 Surv(DurationReadmit, ReadmissionBin) ~ Group1 + Sex + ComorbidSmoke +
 ComorbidCardiovascular + pspline(BMI, df = 3),
 data = Readmit_Frailty,
 id = PatientID2,
 cluster = PatientID2
)

# --- 4. Sensitivity model: add a shared gamma frailty term for Surgeon ---
# Note: frailty() cannot be combined with cluster=/id= robust variance,
# so this is fit without that argument. The partial likelihood (and
# therefore AIC/BIC) is unaffected; only the variance-covariance
# estimator for the primary model differs, which is irrelevant here.
Readmit_Frailty_Warnings <- character(0)

ModelCox4_Frailty <- withCallingHandlers(
 survival::coxph(
 Surv(DurationReadmit, ReadmissionBin) ~ Group1 + Sex + ComorbidSmoke +
 ComorbidCardiovascular + pspline(BMI, df = 3) + frailty(Surgeon),
 data = Readmit_Frailty
 ),
 warning = function(w) {
 Readmit_Frailty_Warnings <<- c(Readmit_Frailty_Warnings, conditionMessage(w))
 invokeRestart("muffleWarning")
 }
)

if (length(Readmit_Frailty_Warnings) > 0) {
 epoxy::epoxy(
 "Model fitting warning {seq_along(Readmit_Frailty_Warnings)}/",
 "{length(Readmit_Frailty_Warnings)}: {Readmit_Frailty_Warnings}",
 .sep = "\n"
 )
} else {
 epoxy::epoxy("Model fitting produced no warnings.")
}

Model fitting warning 1/
1: Inner loop failed to coverge for iterations 2

ModelCox4_Frailty_Table <- tibble::tribble(
 ~Term, ~coef, ~`se(coef)`, ~se2, ~Chisq, ~DF, ~p,
 "Group1Pre-RAS", -0.4309, 0.4496, 0.4387, 0.9184, 1.00, 0.338,
 "Group1Non-RAS", 0.8060, 0.4558, 0.3774, 3.1264, 1.00, 0.077,
 "SexMale", 0.6140, 0.3307, 0.3293, 3.4465, 1.00, 0.063,
 "ComorbidSmokeYes", -0.6300, 1.0228, 1.0214, 0.3795, 1.00, 0.538,
 "ComorbidCardiovascularYes", 0.0000, 0.0000, NA, NA, 1.00, NA,
 "pspline(BMI, df = 3), lin", -0.0199, 0.0233, 0.0232, 0.7310, 1.00, 0.393,
 "pspline(BMI, df = 3), non", NA, NA, NA, 6.3425, 2.05, 0.044,
 "frailty(Surgeon)", NA, NA, NA, 3.9871, 0.99, 0.045
)

ModelCox4_Frailty_Table |>
 gt::gt() |>
 gt::cols_label(
 coef = "Coef", `se(coef)` = "SE(coef)", se2 = "SE2",
 Chisq = "\u03c7\u00b2", DF = "df", p = "p-value"
 ) |>
 gt::fmt_number(columns = c(coef, `se(coef)`, se2, Chisq, DF, p), decimals = 3) |>
 gt::sub_missing(missing_text = "\u2014") |>
 gt::tab_footnote(
 footnote = "pspline (non) and frailty(Surgeon) rows report a penalized-term \u03c7\u00b2 test only; no single coefficient/HR applies.",
 locations = gt::cells_body(
 columns = Term,
 rows = Term %in% c("pspline(BMI, df = 3), non", "frailty(Surgeon)")
 )
 ) |>
 gt::tab_footnote(
 footnote = "Coefficient and SE both 0 reflect a boundary/separation estimate, also present in the primary (non-frailty) model.",
 locations = gt::cells_body(columns = Term, rows = Term == "ComorbidCardiovascularYes")
 ) |>
 knitr::knit_print()

| Table 38: Summary of all-cause readmission model including a frailty variance estimate for surgeon   \| Term \| Coef \| SE(coef) \| SE2 \| χ² \| df \| p-value \| \| --- \| --- \| --- \| --- \| --- \| --- \| --- \| \| Group1Pre-RAS \| -0.431 \| 0.450 \| 0.439 \| 0.918 \| 1.000 \| 0.338 \| \| Group1Non-RAS \| 0.806 \| 0.456 \| 0.377 \| 3.126 \| 1.000 \| 0.077 \| \| SexMale \| 0.614 \| 0.331 \| 0.329 \| 3.446 \| 1.000 \| 0.063 \| \| ComorbidSmokeYes \| -0.630 \| 1.023 \| 1.021 \| 0.380 \| 1.000 \| 0.538 \| \| ComorbidCardiovascularYes*^1^* \| 0.000 \| 0.000 \| — \| — \| 1.000 \| — \| \| pspline(BMI, df = 3), lin \| -0.020 \| 0.023 \| 0.023 \| 0.731 \| 1.000 \| 0.393 \| \| pspline(BMI, df = 3), non*^2^* \| — \| — \| — \| 6.343 \| 2.050 \| 0.044 \| \| frailty(Surgeon)*^2^* \| — \| — \| — \| 3.987 \| 0.990 \| 0.045 \| \| *^1^*Coefficient and SE both 0 reflect a boundary/separation estimate, also present in the primary (non-frailty) model. \| \| \| \| \| \| \| \| *^2^*pspline (non) and frailty(Surgeon) rows report a penalized-term χ² test only; no single coefficient/HR applies. \| \| \| \| \| \| \| |
| --- | --- | --- | --- | --- | --- | --- | --- | --- | --- | --- | --- | --- | --- | --- | --- | --- | --- | --- | --- | --- | --- | --- | --- | --- | --- | --- | --- | --- | --- | --- | --- | --- | --- | --- | --- | --- | --- | --- | --- | --- | --- | --- | --- | --- | --- | --- | --- | --- | --- | --- | --- | --- | --- | --- | --- | --- | --- | --- | --- | --- | --- | --- | --- | --- | --- | --- | --- | --- | --- | --- | --- | --- | --- | --- | --- | --- | --- |

# --- 5. AIC / BIC comparison ---
# ComorbidCardiovascular sits at a boundary/separation estimate (HR ~0 in
# both models), which leaves its effective df undefined (NaN) in the
# penalized frailty fit and breaks AIC()/BIC()'s internal df sum. The
# primary (non-penalized) model implicitly assigns this coefficient the
# conventional df = 1; the same substitution is applied here so the two
# models are compared on a like-for-like df basis.
Readmit_Frailty_df <- ModelCox4_Frailty$df
Readmit_Frailty_df[is.na(Readmit_Frailty_df)] <- 1
Readmit_Frailty_df_total <- sum(Readmit_Frailty_df)

Readmit_Frailty_LogLik <- as.numeric(logLik(ModelCox4_Frailty))
Readmit_Frailty_NEvents <- ModelCox4_Frailty$nevent

AIC_Frailty_Corrected <- -2 * Readmit_Frailty_LogLik + 2 * Readmit_Frailty_df_total
BIC_Frailty_Corrected <- -2 * Readmit_Frailty_LogLik +
 log(Readmit_Frailty_NEvents) * Readmit_Frailty_df_total

Readmit_Frailty_Comparison <- data.frame(
 Model = c("Primary (no frailty)", "Surgeon shared frailty"),
 AIC = c(AIC(ModelCox4), AIC_Frailty_Corrected),
 BIC = c(BIC(ModelCox4), BIC_Frailty_Corrected)
)

Readmit_Frailty_Comparison_Table <- Readmit_Frailty_Comparison |>
 gt::gt() |>
 gt::tab_header(
 title = "Model Comparison: Primary vs Surgeon Shared Frailty (Readmission)"
 ) |>
 gt::cols_label(Model = "Model", AIC = "AIC", BIC = "BIC") |>
 gt::fmt_number(columns = c(AIC, BIC), decimals = 2)

knitr::knit_print(Readmit_Frailty_Comparison_Table)

| Table 39: Comparison of survival models with and without a frailty variance estimate for surgeon  Table 4: Model Comparison: Primary vs Surgeon Shared Frailty (Readmission)   \| Model \| AIC \| BIC \| \| --- \| --- \| --- \| \| Primary (no frailty) \| 478.06 \| 491.23 \| \| Surgeon shared frailty \| 472.02 \| 486.11 \| |
| --- | --- | --- | --- | --- | --- | --- | --- | --- | --- |

# --- 6. Frailty variance estimate ---
# NOTE: history is a list keyed by term name, not position - confirm with
# names(ModelCox4_Frailty$history) before trusting index-based extraction.
# The top-level $theta field can reflect an intermediate search step rather
# than the converged iterate used by print.coxph - take the final row of
# the iteration history matrix instead, which matches the printed
# "Variance of random effect" value.
Readmit_Frailty_History <- ModelCox4_Frailty$history[["frailty(Surgeon)"]]$history
Readmit_Frailty_Theta <- Readmit_Frailty_History[nrow(Readmit_Frailty_History), "theta"]

# Flag identifiability: if the marginal log-likelihood (c.loglik) varies by
# less than ~0.5 units across the last few search iterations, theta is
# weakly identified at this event count and should be reported as such.
Readmit_Frailty_LLRange <- diff(range(
 tail(Readmit_Frailty_History[, "c.loglik"], 3)
))

# Kendall's tau equivalent for a gamma shared frailty (Balan & Putter 2020):
# tau = theta / (theta + 2) - comparable in spirit (not derivation) to the
# 4.0% surgeon ICC reported for procedure duration.
Readmit_Frailty_Tau <- Readmit_Frailty_Theta / (Readmit_Frailty_Theta + 2)

epoxy::epoxy(
 "Estimated gamma frailty variance for Surgeon: {round(Readmit_Frailty_Theta, 4)}; ",
 "Kendall's tau equivalent: {round(Readmit_Frailty_Tau, 4)}; ",
 "c.loglik range across final iterations: {round(Readmit_Frailty_LLRange, 4)} ",
 "({if (Readmit_Frailty_LLRange < 0.5) 'flat - weakly identified' else 'distinguishable'})"
)

Estimated gamma frailty variance for Surgeon: 0.1335; Kendall's tau equivalent: 0.0626; c.loglik range across final iterations: 0.0634 (flat - weakly identified)

# --- 7. Hazard ratio table, matching existing supplementary style ---
TableCox4_Frailty <- gtsummary::tbl_regression(
 ModelCox4_Frailty,
 exponentiate = TRUE,
 show_single_row = c("Sex", "ComorbidSmoke", "ComorbidCardiovascular"),
 label = list(
 Sex = "Sex (Male)",
 ComorbidSmoke = "Smoking History",
 ComorbidCardiovascular = "Cardiovascular Disease"
 )
)

TableCox4_FrailtyCompare <- gtsummary::tbl_merge(
 list(TableCox4, TableCox4_Frailty),
 tab_spanner = c("Primary model", "Surgeon shared frailty"),
 quiet = TRUE
)

gtsummary::as_flex_table(
 TableCox4_FrailtyCompare
 ) |>
 flextable::set_table_properties(
 layout = "autofit",
 width = 0.15,
 align = "center",
 opts_word = list(split = TRUE)
 )

|  | Primary model | | | Surgeon shared frailty | | |
| --- | --- | --- | --- | --- | --- | --- |
| **Characteristic** | **HR** | **95% CI** | **p-value** | **HR** | **95% CI** | **p-value** |
| Group1 |  |  |  |  |  |  |
| RAS | — | — |  | — | — |  |
| Pre-RAS | 0.66 | 0.28, 1.55 | 0.3 | 0.65 | 0.27, 1.57 | 0.3 |
| Non-RAS | 1.70 | 0.83, 3.52 | 0.15 | 2.24 | 0.92, 5.47 | 0.077 |
| Sex (Male) | 1.81 | 0.95, 3.46 | 0.072 | 1.85 | 0.97, 3.53 | 0.063 |
| Smoking History | 0.55 | 0.07, 4.03 | 0.6 | 0.53 | 0.07, 3.95 | 0.5 |
| Cardiovascular Disease | 0.00 | 0.00, Inf | >0.9 |  |  |  |
| frailty(Surgeon) |  |  |  |  |  | 0.045 |
| Abbreviations: CI = Confidence Interval, HR = Hazard Ratio | | | | | | |

Readmit_Frailty_Chisq <- ModelCox4_Frailty_Table$Chisq[
 ModelCox4_Frailty_Table$Term == "frailty(Surgeon)"
]
Readmit_Frailty_P <- ModelCox4_Frailty_Table$p[
 ModelCox4_Frailty_Table$Term == "frailty(Surgeon)"
]

# Quantify how concentrated Non-RAS readmission events are across surgeons -
# motivated by the cross-tab showing 2 surgeons carry all but 1 event.
Readmit_NonRAS_Total <- sum(Readmit_Surgeon_Group_Events[["Non-RAS"]])
Readmit_NonRAS_Top2 <- sum(
 sort(Readmit_Surgeon_Group_Events[["Non-RAS"]], decreasing = TRUE)[1:2]
)
Readmit_NonRAS_Concentration <- Readmit_NonRAS_Top2 / Readmit_NonRAS_Total

A shared gamma frailty term for Surgeon was added to the primary readmission model. Model fit was meaningfully improved by the inclusion of frailty (AIC: 478.06 vs 472.02; BIC: 491.23 vs 486.11).

The estimated frailty variance was modest in absolute terms (θ = 0.134; Kendall’s τ ≈ 0.063), but the χ² test for the frailty term reached conventional significance (χ² = 3.99, p = 0.045), and both AIC and BIC favoured the frailty-adjusted model. The profile likelihood for θ was flat across the final search iterations (Δc.loglik = 0.063), indicating that while the direction of evidence consistently favours non-zero surgeon-level heterogeneity, the precise magnitude of θ is weakly identified at this event count (~5.4 events per surgeon).

Hazard ratios for Group were broadly similar between the primary and frailty-adjusted models for Pre-RAS (0.66 vs 0.65), but the Non-RAS hazard ratio shifted upward under frailty adjustment (1.7 vs 2.24). A cross-tabulation of Surgeon by Group1 showed that 93% of Non-RAS readmission events were concentrated among two surgeons, indicating that Surgeon and Group are not cleanly separable within the Non-RAS arm. This shift in the Non-RAS hazard ratio is therefore better explained by entanglement between Surgeon and Group1 in this subset than by the frailty model revealing a more accurate causal estimate, and the frailty variance itself may partly reflect unmodelled Group-by-surgeon structure rather than surgeon-level heterogeneity in isolation. The primary (non-frailty) model is therefore retained as the reported analysis, with this sensitivity check presented as a bounded exploration of surgeon-level clustering rather than confirmatory evidence either for or against it.

On this basis, the primary (non-frailty) Cox model is retained as the reported analysis for readmission, and the frailty-adjusted model is presented here as a bounded exploratory sensitivity check rather than as confirmatory evidence either for or against surgeon-level confounding. Unlike the corresponding analysis for procedure duration, this sensitivity check does not rule out that surgeon-level clustering is completely irrelevant. Instead, it identifies a specific, localised source of potential confounding within the Non-RAS comparison that should be considered a limitation when interpreting that comparison.

# 7. Discussion:

## 7.1 STROBE [18] Key Results:

*Critically interpret the findings in the context of the study objectives.*

The key results can be summarised as follows;

1. Introduction of a robotic system leads to a significant, but relatively small reduction in procedure duration.
2. No discernible *main effect* on survival hazard relative to the most common post-operative complications (SSI, VTE, Stiffness, All-cause readmission).
3. A potential *interaction* effect between Sex and the introduction of a robotic system with respect to SSI, although the difference between Groups did not reach significance. Nevertheless, the size of the effect equaled a six-fold increase in SSI in males after the introduction of the robot.

## 7.2 STROBE [19] Limitations:

*Provide a thorough critique of the limitations of the study design, methods, and data.*

The limitations of the analysis can be summarised in the table below and should be prioritised for inclusion in the manuscript. Further work may be required to draw on existing literature to strengthen the argument for impact and/or mitigation measures.

| Table 40: Summary of key limitations of the presented analysis   \| Component \| Limitation \| Impact \| Mitigation \| \| --- \| --- \| --- \| --- \| \| Setting \| Public hospital with highly variable surgeon group \| Variability in perioperative management and detection \| reporting of adverse events. Limit ability to attribute observations to Group assignment. \| Temper language with respect to conclusions and causality of differences observed between Groups. \| \| Design \| Retrospective cohort analysis with inherent biases \| Information bias may change the distribution of outcomes between groups and lead to misleading results \| Restrict dataset to cases that could be verified and with sufficient information to assign to a Group accurately. \| \| Data 1 \| Issues with retrieving hospital data created gaps in the timeline and difficulties with verifying case matches \| Increased the rejection rate within the data pool. Considering the temporal nature of the omissions it may have impacted one group more than the others. \| The overall sample seems sufficient for adequately powered comparison between groups. However, bias with respect to generalisability cannot be fully removed. \| \| Data 2 \| Clinical flow and population returned few events for certain adverse categories, limiting the capacity for adjusted analysis \| The robot RAS introduction could not be fully assessed for all adverse events. \| Future work should consider multi-state models to assess incidence of transition between adverse event states across the follow up period. \| \| Methods \| Model fitting was challenging for some outcome measures and may need further refinement. \| The procedure duration model in particular may present erroneous results and should be interpreted cautiously. \| Further refinement of models comparing procedure duration in this context and population should be prioritised in future work. \| \| Methods (sensitivity analysis) \| A post-hoc shared frailty sensitivity analysis for Surgeon (readmission outcome) found that Surgeon and Group1 were not cleanly separable within the Non-RAS arm, where two surgeons accounted for nearly all readmission events \| The Non-RAS hazard ratio shifted under frailty adjustment in a way that reflects this entanglement rather than a more accurate causal estimate; the frailty variance itself may partly capture unmodelled Group-by-surgeon structure \| Primary (non-frailty) model was retained as the reported analysis; the frailty model is presented as a sensitivity check, consistent with the existing surgeon-composition limitation \| |
| --- | --- | --- | --- | --- | --- | --- | --- | --- | --- | --- | --- | --- | --- | --- | --- | --- | --- | --- | --- | --- | --- | --- | --- | --- | --- | --- | --- | --- |

## 7.3 STROBE [20] Interpretation:

*Assess whether the interpretation is adequately cautious and whether the comparisons to related literature are appropriate. Assess the implications of the findings for clinical practice and the need for further research, considering alternative interpretations.*

The study co-authors need to take into account the main findings in the context of other components of this report to make a balanced interpretation of the findings.

The key suggestion here is to better understand the medium-term risks of surgical site infection (SSI) on the development of periprosthetic joint infection within the first 12-24months after surgery. Given the relationship between the soft tissue and the knee joint (compared to the hip) it is posited that SSI may be a key risk factor for developing PJI within this period, however more work with the relevant literature is required.

## 7.4 STROBE [21] Generalizability:

*Evaluate the strength of evidence supporting the external validity of the findings.*

It is recommended that the population characteristics and healthcare setting (hospital type, size, location) are considered when comparing the findings to relevant studies in the contemporary literature.

The fact that the patient population is attending a public, medium-sized hospital in a metropolitan setting in an Australian capital city poses key constraints on the ability to directly compare findings between this study and others. These aspects need to be taken into account when interpreting the results in the context of the related literature.

The analysis design is also relatively novel with respect to the control groups constructed from the retrospective data, as well as the robust approach to the survival analysis (few studies attempt to adjust for covariates when comparing groups). In addition, the use of directed acyclic graphs to establish the adjustment set for each model is not commonly utilised in the relevant literature (although it should be).

## 7.5 Comparison with Existing Literature:

*Critically compare findings with previous research, assessing consistency and discrepancies.*

The notes on generalisability above should be integrated into the literature comparison in place within the manuscript so far.

# 8. Ethical Considerations:

## 8.1 Informed Consent:

*Critically analyze the adequacy of procedures for obtaining informed consent from participants.*

A waiver of consent was granted to conduct chart review of the relevant patient records (see below).

## 8.2 Ethical Approval:

*Evaluate whether the study received appropriate approval from an ethics committee or institutional review board.*

The study received HREC approval (HREC approval number) from the hospital HREC prior to commencement of chart review.

## 8.3 Privacy and Confidentiality:

*Assess the adequacy of measures taken to protect participants’ privacy and confidentiality. Beneficence and Non-maleficence: Critically evaluate any potential risks or benefits to participants and how they were addressed.*

Participant information was at risk of breach or leaking into the public domain. This risk was addressed by securing study materials using industry-standard methods to encrypt data at-rest within user-restricted data assets and in transit between the health service and the research team. Chart review was performed by approved persons with appropriate access and system credentials with training and experience on patient privacy and record management.

# 9. Conflicts of Interest:

## 9.1 STROBE [22] Funding:

*Assess the degree of transparency to which the source of funding and role of the funders have played in the study/article.*

The funding for this study was provided by the Department Research Fund. Some individuals responsible for administering the funds are co-authors of the study.

## 9.2 Disclosure:

*Evaluate the transparency and completeness of disclosures regarding conflicts of interest among the study authors or sponsors.*

Author CS declares institutional funding (EBM Analytics) received for a project assessing the clinical outcomes of a competitor system.

## 9.3 Potential for Biases:

*Critically assess whether conflicts of interest could have influenced the study design, analysis, or interpretation of results.*

Conflicts of interest could be perceived to influence the analysis or interpretation of results. Some authors are conflicted by their relationship to the hospital and the department in which the RAS is installed, others have a perceived conflict with respect to competitor systems available in the local market. These conflicts have been declared in the context of the study submission and are available for readers to interpret.

# 10. References:

*Evaluate the quality and relevance of cited references, including any potential biases or omissions.*

No relevant commentary.

# 11. Analysis References

Acuña, Alexander J., Linsen T. Samuel, Jaret M. Karnuta, Assem A. Sultan, Andrew M. Swiergosz, and Atul F. Kamath. 2020. “What Factors Influence Operative Time in Total Knee Arthroplasty? A 10-Year Analysis in a National Sample.” *The Journal of Arthroplasty* 35 (3): 621–27. https://doi.org/10.1016/j.arth.2019.10.054.

Aden-Buie, Garrick. 2023. *epoxy: String Interpolation for Documents, Reports and Apps*. https://CRAN.R-project.org/package=epoxy.

Allaire, JJ, Yihui Xie, Christophe Dervieux, et al. 2026. *rmarkdown: Dynamic Documents for r*. https://github.com/rstudio/rmarkdown.

Arel-Bundock, Vincent, Noah Greifer, and Andrew Heiss. 2024. “How to Interpret Statistical Models Using marginaleffects for R and Python.” *Journal of Statistical Software* 111 (9): 1–32. https://doi.org/10.18637/jss.v111.i09.

Baayen, R. H., D. J. Davidson, and D. M. Bates. 2008. “Mixed-Effects Modeling with Crossed Random Effects for Subjects and Items.” *Journal of Memory and Language* 59 (4): 390–412. https://doi.org/10.1016/j.jml.2007.12.005.

Badawy, Mona, Birgitte Espehaug, Anne Marie Fenstad, et al. 2017. “Patient and Surgical Factors Affecting Procedure Duration and Revision Risk Due to Deep Infection in Primary Total Knee Arthroplasty.” *BMC Musculoskeletal Disorders* 18 (1). https://doi.org/10.1186/s12891-017-1915-4.

Barbone, Jordan Mark, and Jan Marvin Garbuszus. 2026. *Openxlsx2: Read, Write and Edit “xlsx” Files*. https://janmarvin.github.io/openxlsx2/.

Barrett, Malcolm. 2024. *ggdag: Analyze and Create Elegant Directed Acyclic Graphs*. https://CRAN.R-project.org/package=ggdag.

Bates, Douglas, Martin Mächler, Ben Bolker, and Steve Walker. 2015. “Fitting Linear Mixed-Effects Models Using lme4.” *Journal of Statistical Software* 67 (1): 1–48. https://doi.org/10.18637/jss.v067.i01.

Benoit, Kenneth, David Muhr, and Kohei Watanabe. 2021. *stopwords: Multilingual Stopword Lists*. https://CRAN.R-project.org/package=stopwords.

Bolker, Ben, and David Robinson. 2026. *broom.mixed: Tidying Methods for Mixed Models*. https://CRAN.R-project.org/package=broom.mixed.

Brown, Kelsey E., Michael J. Flores, Gerard Slobogean, David Shearer, Ida Leah Gitajn, and Saam Morshed. 2023. “Simple Design and Analysis Strategies for Solving Problems in Observational Orthopaedic Clinical Research.” *OTA International: The Open Access Journal of Orthopaedic Trauma* 6 (2S). https://doi.org/10.1097/oi9.0000000000000239.

Bryan, Jennifer. 2023. *Googlesheets4: Access Google Sheets Using the Sheets API V4*. https://CRAN.R-project.org/package=googlesheets4.

Carroll, Orlagh U., Tim P. Morris, and Ruth H. Keogh. 2020. “How Are Missing Data in Covariates Handled in Observational Time-to-Event Studies in Oncology? A Systematic Review.” *BMC Medical Research Methodology* 20 (1). https://doi.org/10.1186/s12874-020-01018-7.

Cheng, Yun-Jiu, Zhi-Hao Liu, Feng-Juan Yao, et al. 2013. “Current and Former Smoking and Risk for Venous Thromboembolism: A Systematic Review and Meta-Analysis.” *PLoS Medicine* 10 (9): e1001515. https://doi.org/10.1371/journal.pmed.1001515.

Cook, Jonathan A, Thomas Bruckner, Graeme S MacLennan, and Christoph M Seiler. 2012. “Clustering in Surgical Trials - Database of Intracluster Correlations.” *Trials* 13 (1). https://doi.org/10.1186/1745-6215-13-2.

Davies, G. Matt, and Alan Gray. 2015. “Don’t Let Spurious Accusations of Pseudoreplication Limit Our Ability to Learn from Natural Experiments (and Other Messy Kinds of Ecological Monitoring).” *Ecology and Evolution* 5 (22): 5295–304. https://doi.org/10.1002/ece3.1782.

Dayim, Alim. 2026. *consort: Create Consort Diagram*. https://CRAN.R-project.org/package=consort.

Deischinger, Carola, Elma Dervic, Stephan Nopp, Michaela Kaleta, Peter Klimek, and Alexandra Kautzky-Willer. 2022. “Diabetes Mellitus Is Associated with a Higher Relative Risk for Venous Thromboembolism in Females Than in Males.” *Diabetes Research and Clinical Practice* 194 (December): 110190. https://doi.org/10.1016/j.diabres.2022.110190.

Denz, Robin, Renate Klaaßen-Mielke, and Nina Timmesfeld. 2023. “A Comparison of Different Methods to Adjust Survival Curves for Confounders.” *Statistics in Medicine* 42 (10): 1461–79. https://doi.org/10.1002/sim.9681.

Ergina, P. L., J. S. Barkun, P. McCulloch, J. A. Cook, and D. G. Altman. 2013. “IDEAL Framework for Surgical Innovation 2: Observational Studies in the Exploration and Assessment Stages.” *BMJ* 346 (jun18 3): f3011–11. https://doi.org/10.1136/bmj.f3011.

Fellows, Ian. 2018. *wordcloud: Word Clouds*. https://CRAN.R-project.org/package=wordcloud.

Gerds, Thomas A., and Michael W. Kattan. 2021. *Medical Risk Prediction Models: With Ties to Machine Learning (1st Ed.)*. Chapman; Hall/CRC. https://doi.org/10.1201/9781138384484.

Gerds, Thomas Alexander, Johan Sebastian Ohlendorff, and Brice Ozenne. 2026. *riskRegression: Risk Regression Models and Prediction Scores for Survival Analysis with Competing Risks*. https://CRAN.R-project.org/package=riskRegression.

Gohel, David, and Panagiotis Skintzos. 2026. *flextable: Functions for Tabular Reporting*. https://CRAN.R-project.org/package=flextable.

Gould, Daniel, Michelle M Dowsey, Tim Spelman, et al. 2021. “Patient-Related Risk Factors for Unplanned 30-Day Hospital Readmission Following Primary and Revision Total Knee Arthroplasty: A Systematic Review and Meta-Analysis.” *Journal of Clinical Medicine* 10 (1): 134. https://doi.org/10.3390/jcm10010134.

Grolemund, Garrett, and Hadley Wickham. 2011. *Dates and Times Made Easy with Lubridate*. 40. https://www.jstatsoft.org/v40/i03/.

Healy, William L., Craig J. Della Valle, Richard Iorio, et al. 2013. “Complications of Total Knee Arthroplasty: Standardized List and Definitions of the Knee Society.” *Clinical Orthopaedics & Related Research* 471 (1): 215–20. https://doi.org/10.1007/s11999-012-2489-y.

Heifner, John J., Philip A. Sakalian, Robert J. Rowland, and Arturo Corces. 2023. “Local Adiposity May Be a More Reliable Predictor for Infection Than Body Mass Index Following Total Knee Arthroplasty: A Systematic Review.” *Journal of Experimental Orthopaedics* 10 (1). https://doi.org/10.1186/s40634-023-00680-2.

Holst, Klaus K., Thomas H. Scheike, and Jacob B. Hjelmborg. 2016. “The Liability Threshold Model for Censored Twin Data.” *Computational Statistics and Data Analysis* 93: 324–35. https://doi.org/10.1016/j.csda.2015.01.014.

Horikoshi, Masaaki, and Yuan Tang. 2018. *ggfortify: Data Visualization Tools for Statistical Analysis Results*. https://CRAN.R-project.org/package=ggfortify.

Iannone, Richard, Joe Cheng, Barret Schloerke, et al. 2026. *gt: Easily Create Presentation-Ready Display Tables*. https://CRAN.R-project.org/package=gt.

Izrailev, Sergei. 2024. *tictoc: Functions for Timing r Scripts, as Well as Implementations of “Stack” and “StackList” Structures*. https://CRAN.R-project.org/package=tictoc.

Kassambara, Alboukadel, Marcin Kosinski, and Przemyslaw Biecek. 2026. *survminer: Drawing Survival Curves Using “ggplot2”*. https://CRAN.R-project.org/package=survminer.

Kay, Matthew. 2024. “ggdist: Visualizations of Distributions and Uncertainty in the Grammar of Graphics.” *IEEE Transactions on Visualization and Computer Graphics* 30 (1): 414–24. https://doi.org/10.1109/TVCG.2023.3327195.

Kay, Matthew. 2025. *ggdist: Visualizations of Distributions and Uncertainty*. https://doi.org/10.5281/zenodo.3879620.

Koenker, Roger. 2025. *quantreg: Quantile Regression*. https://CRAN.R-project.org/package=quantreg.

Konopka, Jaclyn A., David A. Bloom, Kyle W. Lawrence, Jacob F. Oeding, Ran Schwarzkopf, and Claudette M. Lajam. 2023. “Non-English Speakers and Socioeconomic Minorities Are Significantly Less Likely to Complete Patient-Reported Outcome Measures for Total Hip and Knee Arthroplasty: Analysis of 16,119 Cases.” *The Journal of Arthroplasty* 38 (7): S69–77. https://doi.org/10.1016/j.arth.2023.01.005.

Kuhn, Max, and Hadley Wickham. 2020. *Tidymodels: A Collection of Packages for Modeling and Machine Learning Using Tidyverse Principles.* https://www.tidymodels.org.

Kuitunen, Ilari, Ville T. Ponkilainen, Mikko M. Uimonen, Antti Eskelinen, and Aleksi Reito. 2021. “Testing the Proportional Hazards Assumption in Cox Regression and Dealing with Possible Non-Proportionality in Total Joint Arthroplasty Research: Methodological Perspectives and Review.” *BMC Musculoskeletal Disorders* 22 (1). https://doi.org/10.1186/s12891-021-04379-2.

Larmarange, Joseph, and Daniel D. Sjoberg. 2025. *broom.helpers: Helpers for Model Coefficients Tibbles*. https://CRAN.R-project.org/package=broom.helpers.

Lazic, Stanley E. 2010. “The Problem of Pseudoreplication in Neuroscientific Studies: Is It Affecting Your Analysis?” *BMC Neuroscience* 11 (1). https://doi.org/10.1186/1471-2202-11-5.

Lee, Binglong, Milad Ebrahimi, Nalan Ektas, et al. 2020. “Implementation and Quality Assessment of a Clinical Orthopaedic Registry in a Public Hospital Department.” *BMC Health Services Research* 20 (1). https://doi.org/10.1186/s12913-020-05203-8.

Maas, Cora J. M., and Joop J. Hox. 2005. “Sufficient Sample Sizes for Multilevel Modeling.” *Methodology* 1 (3): 86–92. https://doi.org/10.1027/1614-2241.1.3.86.

Malehi, Amal Saki, Fatemeh Pourmotahari, and Kambiz Ahmadi Angali. 2015. “Statistical Models for the Analysis of Skewed Healthcare Cost Data: A Simulation Study.” *Health Economics Review* 5 (1). https://doi.org/10.1186/s13561-015-0045-7.

Oltean, Hanna, and Joel J Gagnier. 2015. “Use of Clustering Analysis in Randomized Controlled Trials in Orthopaedic Surgery.” *BMC Medical Research Methodology* 15 (1). https://doi.org/10.1186/s12874-015-0006-1.

Owen, Aaron R., Meagan E. Tibbo, Andre J. van Wijnen, Mark W. Pagnano, Daniel J. Berry, and Matthew P. Abdel. 2021. “Acquired Idiopathic Stiffness After Contemporary Total Knee Arthroplasty: Incidence, Risk Factors, and Results over 25 Years.” *The Journal of Arthroplasty* 36 (8): 2980–85. https://doi.org/10.1016/j.arth.2021.03.051.

Pedersen, Thomas Lin. 2025. *patchwork: The Composer of Plots*. https://CRAN.R-project.org/package=patchwork.

Poultsides, Lazaros A., Yan Ma, Alejandro Gonzalez Della Valle, Ya-Lin Chiu, Thomas P. Sculco, and Stavros G. Memtsoudis. 2013. “In-Hospital Surgical Site Infections After Primary Hip and Knee Arthroplasty Incidence and Risk Factors.” *The Journal of Arthroplasty* 28 (3): 385–89. https://doi.org/10.1016/j.arth.2012.06.027.

R Core Team. 2024. *R: A Language and Environment for Statistical Computing*. R Foundation for Statistical Computing. https://www.R-project.org/.

Rinker, Tyler W., and Dason Kurkiewicz. 2018. *pacman: Package Management for R*. Buffalo, New York. http://github.com/trinker/pacman.

Scheike, Thomas H., and Klaus K. Holst. 2014. “A Practical Guide to Family Studies with Lifetime Data.” *Annual Review of Statistics and Its Application* 9: 47–69. https://doi.org/10.1146/annurev-statistics-040120-024253.

Scheike, Thomas H., Klaus K. Holst, and Jacob B. Hjelmborg. 2014. “Estimating Heritability for Cause Specific Mortality Based on Twin Studies.” *Lifetime Data Analysis* 20 (2): 210–33. https://doi.org/10.1007/s10985-013-9244-x.

Shrout, Patrick E., and Joseph L. Fleiss. 1979. “Intraclass Correlations: Uses in Assessing Rater Reliability.” *Psychological Bulletin* 86 (2): 420–28. https://doi.org/10.1037/0033-2909.86.2.420.

Silge, Julia, and David Robinson. 2016. “tidytext: Text Mining and Analysis Using Tidy Data Principles in r.” *JOSS* 1 (3). https://doi.org/10.21105/joss.00037.

Simon, Samantha, and Brian Hollenbeck. 2022. “Risk Factors for Surgical Site Infections in Knee and Hip Arthroplasty Patients.” *American Journal of Infection Control* 50 (2): 214–16. https://doi.org/10.1016/j.ajic.2021.11.006.

Sjoberg, Daniel D., Mark Baillie, Charlotta Fruechtenicht, Steven Haesendonckx, and Tim Treis. 2025. *ggsurvfit: Flexible Time-to-Event Figures*. https://CRAN.R-project.org/package=ggsurvfit.

Sjoberg, Daniel D., and Teng Fei. 2026. *tidycmprsk: Competing Risks Estimation*. https://CRAN.R-project.org/package=tidycmprsk.

Sjoberg, Daniel D., Karissa Whiting, Michael Curry, Jessica A. Lavery, and Joseph Larmarange. 2021. “Reproducible Summary Tables with the Gtsummary Package.” *The R Journal* 13: 570–80. https://doi.org/10.32614/RJ-2021-053.

Tang, Yuan, Masaaki Horikoshi, and Wenxuan Li. 2016. “ggfortify: Unified Interface to Visualize Statistical Result of Popular r Packages.” *The R Journal* 8 (2): 474–85. https://doi.org/10.32614/RJ-2016-060.

Tennant, Peter W G, Eleanor J Murray, Kellyn F Arnold, et al. 2020. “Use of Directed Acyclic Graphs (DAGs) to Identify Confounders in Applied Health Research: Review and Recommendations.” *International Journal of Epidemiology* 50 (2): 620–32. https://doi.org/10.1093/ije/dyaa213.

Terry M. Therneau, and Patricia M. Grambsch. 2000. *Modeling Survival Data: Extending the Cox Model*. Springer.

Therneau, Terry M. 2024. *A Package for Survival Analysis in r*. https://CRAN.R-project.org/package=survival.

Therneau, Terry M. 2026. *A Package for Survival Analysis in r*. https://CRAN.R-project.org/package=survival.

Therneau, Terry M., and Patricia M. Grambsch. 2000. *Modeling Survival Data: Extending the Cox Model*. Springer New York. https://doi.org/10.1007/978-1-4757-3294-8.

Tibbo, Meagan E., Afton K. Limberg, Christopher G. Salib, et al. 2019. “Acquired Idiopathic Stiffness After Total Knee Arthroplasty.” *Journal of Bone and Joint Surgery* 101 (14): 1320–30. https://doi.org/10.2106/jbjs.18.01217.

Tierney, Nicholas, and Dianne Cook. 2023. “Expanding Tidy Data Principles to Facilitate Missing Data Exploration, Visualization and Assessment of Imputations.” *Journal of Statistical Software* 105 (7): 1–31. https://doi.org/10.18637/jss.v105.i07.

van Buuren, Stef, and Karin Groothuis-Oudshoorn. 2011. “mice: Multivariate Imputation by Chained Equations in r.” *Journal of Statistical Software* 45 (3): 1–67. https://doi.org/10.18637/jss.v045.i03.

Vandenbroucke, Jan P, Erik von Elm, Douglas G Altman, et al. 2007. “Strengthening the Reporting of Observational Studies in Epidemiology (STROBE): Explanation and Elaboration.” *PLoS Medicine* 4 (10): e297. https://doi.org/10.1371/journal.pmed.0040297.

Wickham, Hadley. 2023. *Stringr: Simple, Consistent Wrappers for Common String Operations*. https://CRAN.R-project.org/package=stringr.

Wickham, Hadley, Mara Averick, Jennifer Bryan, et al. 2019. “Welcome to the tidyverse.” *Journal of Open Source Software* 4 (43): 1686. https://doi.org/10.21105/joss.01686.

Wickham, Hadley, and Jennifer Bryan. 2023. *Readxl: Read Excel Files*. https://CRAN.R-project.org/package=readxl.

Wickham, Hadley, and Lionel Henry. 2023. *Purrr: Functional Programming Tools*. https://CRAN.R-project.org/package=purrr.

Wickham, Hadley, Thomas Lin Pedersen, and Dana Seidel. 2025. *scales: Scale Functions for Visualization*. https://CRAN.R-project.org/package=scales.

Xie, Yihui. 2014. “knitr: A Comprehensive Tool for Reproducible Research in R.” In *Implementing Reproducible Computational Research*, edited by Victoria Stodden, Friedrich Leisch, and Roger D. Peng. Chapman; Hall/CRC.

Xie, Yihui. 2015. *Dynamic Documents with R and Knitr*. 2nd ed. Chapman; Hall/CRC. https://yihui.org/knitr/.

Xie, Yihui. 2025. *knitr: A General-Purpose Package for Dynamic Report Generation in R*. https://yihui.org/knitr/.

Xie, Yihui, J. J. Allaire, and Garrett Grolemund. 2018. *R Markdown: The Definitive Guide*. Chapman; Hall/CRC. https://yihui.org/rmarkdown/.

Xie, Yihui, Christophe Dervieux, and Emily Riederer. 2020. *R Markdown Cookbook*. Chapman; Hall/CRC. https://yihui.org/rmarkdown-cookbook.

Yung, Godwin, and Yi Liu. 2024. *npsurvSS: Sample Size and Power Calculation for Common Non-Parametric Tests in Survival Analysis*. https://CRAN.R-project.org/package=npsurvSS.

Zhang, Jie, Zhihao Chen, Jilin Zheng, Steffen J Breusch, and Jing Tian. 2015. “Risk Factors for Venous Thromboembolism After Total Hip and Total Knee Arthroplasty: A Meta-Analysis.” *Archives of Orthopaedic and Trauma Surgery* 135 (6): 759–72. https://doi.org/10.1007/s00402-015-2208-8.
